# mRNA transcription profile of potato (*Solanum tuberosum* L.) exposed to ultrasound during different stages of in vitro plantlet development

**DOI:** 10.1007/s11103-019-00876-0

**Published:** 2019-04-29

**Authors:** Judit Dobránszki, Norbert Hidvégi, Andrea Gulyás, Jaime A. Teixeira da Silva

**Affiliations:** Research Institute of Nyíregyháza, IAREF, University of Debrecen, P.O. Box 12, Nyíregyháza, 4400 Hungary

**Keywords:** Abiotic stress, Antioxidant, DEG, Enzyme, Plant growth, Ultrasonication, Wounding

## Abstract

**Key message:**

In response to an ultrasound pulse, several hundred DEGs, including in response to stress, were up- or down-regulated in in vitro potato plantlets. Despite this abiotic stress, plantlets survived.

**Abstract:**

Ultrasound (US) can influence plant growth and development. To better understand the genetic mechanism underlying the physiological response of potato to US, single-node segments of four-week-old in vitro plantlets were subjected to US at 35 kHz for 20 min. Following mRNA purification, 10 cDNA libraries were assessed by RNA-seq. Significantly differentially expressed genes (DEGs) were categorized by gene ontology or Kyoto Encyclopedia of Genes and Genomes identifiers. The expression intensity of 40,430 genes was studied. Several hundred DEGs associated with biosynthesis, carbohydrate metabolism and catabolism, cellular protein modification, and response to stress, and which were expressed mainly in the extracellular region, nucleus, and plasma membrane, were either up- or down-regulated in response to US. RT-qPCR was used to validate RNA-seq data of 10 highly up- or down-regulated DEGs, and both Spearman and Pearson correlations between SeqMonk LFC and RT-qPCR LFC were highly positive (0.97). This study examines how some processes evolved over time (0 h, 24 h, 48 h, 1 week and 4 weeks) after an abiotic stress (US) was imposed on in vitro potato explants, and provides clues to the temporal dynamics in DEG-based enzyme functions in response to this stress. Despite this abiotic stress, plantlets survived.

**Electronic supplementary material:**

The online version of this article (10.1007/s11103-019-00876-0) contains supplementary material, which is available to authorized users.

## Introduction

Plants, being sessile organisms, are constantly exposed to environmental stresses that they cannot avoid and which have physiological and developmental effects. Both sound and ultrasound (US) constitute a form of mechanical stress to plants in the form of pressure waves. The ability of plants to adapt to environmental stresses would thus be important to ensure their evolutionary continuity. Sound or US waves, which are one form of information transfer from the environment, are faster and have a lower energy investment than chemical stimuli (Gagliano 2013; Telewski 2006).

Sound and US, which plants perceive as an external mechanical force or as an abiotic stressor, can modify growth and development as a way to adjust to their environment, including the effect of US with low frequency (≤ 60 kHz) in seed germination (Creath and Schwartz 2004), seedling and plant growth (Collins and Foreman 2001; Qi et al. 2010). Plantlets that are irradiated in vitro by US waves may experience increased growth, or organogenesis may be triggered, but the response depends on the plant species as well as the frequency and period of exposure to US (Teixeira da Silva and Dobránszki 2014a).

Ultrasonication, which involves treatment with or the application of US, causes changes in the permeability, elasticity and viscosity of cell membranes, transport through membranes, as well as the conformation and activity of membrane-bound enzymes and enzymes involved in hormonal regulation and in stress response by triggering the antioxidant defence system (Rokhina et al. 2009; Wang et al. 2001, 2004, 2009; Wei et al. 2012), including in potato (*Solanum tuberosum* L.) (Dobránszki et al. 2017).

There is evidence of the effect of sound on gene expression, even if studies on the effect of sonication on changes in mRNA transcription in plants are limited. Generally, treatment with acoustic sound is referred to as sonication whereas treatment with US is referred to as ultrasonication. In chrysanthemum (*Dendranthema grandiflorum* Ramat.), even though sonication at 1 kHz and 100 dB did not induce changes in DNA content, there was a significant increase in the RNA and protein content (Shao et al. 2008; Wang et al. 2003), which indicates altered gene expression in response to sound waves. When hybrid *Cymbidium* Twilight Moon ‘Day Light’ protocorm-like bodies (PLBs) were irradiated by sound waves (60 Hz, for 5 and 10 min), there was an increase in transient endopolyploidy while PLB growth and the conversion to plantlets improved (Teixeira da Silva and Dobránszki 2014b). When Jeong et al. (2008) used a sound-treated subtractive library and mRNA expression analyses (Northern blots and/or RT-PCR) to assess rice (*Oryza sativa* L. ‘Donggin’), they detected four genes, including the *rbcS* (ribulose-1,5-bisphosphate carboxylase/oxygenase small subunit) and *ald* (aldolase) genes, both of which were responsive to sound. *Ald* was subsequently selected to investigate how this gene responds to different sound frequencies. At 125 Hz, 250 Hz, and 1 kHz, *Ald* was up-regulated, i.e., its mRNA expression increased significantly, but at 50 Hz, it was down-regulated (Jeong et al. 2008). When *Arabidopsis thaliana* L. was sonicated at five frequencies, global RNA and protein profiling revealed alterations in the plant’s ability to scavenge reactive oxygen species (ROS), as well as changes to primary and phytohormonal metabolism (Ghosh et al. 2016).

Potato is the most important tuber crop for which a wealth of genetic studies, molecular breeding and genomic information is available (Watanabe 2015). At the start of this decade, the potato genome was sequenced (The Potato Genome Sequencing Consortium 2011). One of the more powerful technologies that emerged after this feat that has allowed a deeper understanding of the potato genome is next generation sequencing, such as RNA-seq, to carry out differential gene expression analysis (Bykova et al. 2017). RNA-seq has been used to better understand the transcriptomics of compatible and incompatible interactions between potato blight (*Phytophthora infestans*) and potato (Ali et al. 2014). RNA-seq was also used to appreciate differences in expression levels of 39 nitrogen-responsive genes in three potato cultivars (Gálvez et al. 2016). This technique was also used to assess the differential expression of genes in different organs and tissues, including during tuber development (Lakhotia et al. 2014). More importantly, and somewhat related to our ongoing research on abiotic stress in potato, RNA-seq was used to understand the expression profile of genes involved in drought and water stimulus (Gong et al. 2015). For these reasons, RNA-seq served as the transcriptomic method of choice for this study.

In a recent study by our group (Dobránszki et al. 2017), we indicated that treatment of single-node in vitro potato explants with US waves (35 kHz, at 70 W for 20 min) changed the ascorbate–glutathione pathway and the redox state of the plant material, i.e., single-node explants and developing in vitro shoots. Ascorbate peroxidase and glutathione reductase activity and the concentration of low molecular weight antioxidants such as glutathione, ascorbic acid and tocopherol increased significantly immediately (0 h) or 24 h after ultrasonication, which is an expected response to abiotic stress. An after-effect of that US treatment was an increase in shoot growth of in vitro plantlets by the end of the 4 week (w) subculture period, i.e., the application of 35 kHz for 20 min appears to constitute only a mild initial stress, and then further appears to serve as a positive growth stimulant from 24 h after US application until the fourth week of growth. In this study, we investigated the changes in gene expression (mRNA transcription), using significantly annotated differentially expressed genes (DEGs) corresponding to specific enzyme functions, at different phases of in vitro growth of potato plantlets derived from single-node segments that had been ultrasonicated. The primary objective was to better appreciate the effect of US on plant biochemical pathways.

## Materials and methods

### Plant material, ultrasonication conditions and sample collection

Single-node segments with a single leaf were cut from 4-w-old in vitro potato plantlets (cv. Desirée) and used for the experiment. The culture conditions, medium and vessels used were the same as in our earlier study on the effects of ultrasonication on the antioxidant defence system and subsequent growth and development of in vitro potato (Dobránszki et al. 2017). Immediately after placing explants horizontally onto medium, they were ultrasonicated in an ultrasonication unit at 35 kHz (US) and at 70 W for 20 min, as described in Dobránszki et al. (2017). Ultrasonicated and non-ultrasonicated (treatment control) explants were placed on shoot regeneration medium (Murashige and Skoog 1962) medium without plant growth regulators and subcultured for 4 w. Both ultrasonicated and non-ultrasonicated plant material was sampled at 0 h, 24 h, 48 h, 1 w and 4 w after ultrasonication. Samples (explants at 0, 24 and 48 h; leaves + stems + roots at 1 w and 4 w) were stored immediately at − 80 °C until further analysis.

### Total RNA isolation and removal of rRNA

Total RNA was purified from the 10 potato samples as three biological replicates each that were pooled using the Direct-zol™ kit (Zymo Research, Irvine, CA, USA) with TRIzol reagent based on the manufacturer’s protocol. After purifying total RNA, three quality control (QC) methods were applied: (1) microcapillary electrophoresis with an Implen n50 nanophotometer (Implen, Munich, Germany) for preliminary quantification; (2) agarose gel electrophoresis to assess RNA degradation and potential contamination; (3) Agilent Bioanalyzer 2100 system (Agilent, Santa Clara, CA, USA). After the three-step QC, mRNA capturing, cDNA library preparation and sequencing were performed by Novogene (Beijing, China), a genome sequencing company. mRNA from the ten potato samples was enriched using oligo(dT) beads. For long non-coding libraries, rRNA was removed using the Plant Ribo-Zero rRNA removal kit (Illumina, San Diego, CA, USA) using the manufacturer’s protocol, resulting in mRNA (rRNA-depleted RNA-seq).

### mRNA library construction for next generation sequencing

Using the Illumina TruSeq Stranded mRNA kit, mRNA was first randomly fragmented by adding fragmentation buffer, then cDNA was synthesized with an mRNA template and a random hexamer primer, after which a custom second-strand synthesis buffer, dNTPs, RNase H and DNA polymerase I were added to initiate second-strand synthesis. The second step involved terminal repair, A ligation and sequencing adaptor ligation, after which the double-stranded cDNA library was completed through size selection and PCR enrichment. The quality of the cDNA library was assessed in three ways: (1) Qubit 2.0 fluorometric quantitation (Thermo Fischer Scientific, Waltham, MA, USA), mainly to assess the library concentration; (2) Agilent Bioanalyzer 2100 (Agilent) to test insert size; (3) AriaMX qPCR (Agilent) to quantify precisely the effective library concentration. The 30 mRNA libraries (10 treatments × three biological replicates) derived from the three biological replicates were pooled into two qualified libraries.

### Sequencing

The ten qualified libraries were fed into the HiSeq 2500 sequencer (Illumina) after pooling according to its effective concentration and expected data volume. For deep sequencing, the ten libraries were sequenced with 150 bp paired-end reads and the expected data volume was 100 M reads/sample. For each qualified library, three biological replicates were sequenced and pooled into one library.

### Bioinformatic processing of RNA-seq datasets

We downloaded the *Solanum tuberosum* annotated whole genome from the Ensembl Plant database (SolTub 3.0: https://plants.ensembl.org/Solanum_tuberosum/Info/Index) which we used as the reference genome for the RNA-seq datasets. Trimming was performed to remove both poor-quality reads and adapter sequences using TrimGalore v0.5.0 (https://github.com/FelixKrueger/TrimGalore) with default parameters. In addition, 10 bp were removed from the 5′ end of the reads to avoid sequence biases which cause oligo(dT) and random hexamer primers (Hansen et al. 2010). Trimmed reads were aligned to the reference genome with HISAT2 v2.1.0 (Kim et al. 2015) using the paired-end mode. Known splice sites were specified from the splice sites file built from GTF annotation files which were downloaded from the SolTub 3.0 Ensembl Plant database using the HISAT2 python script.

### Analysis of RNA-seq datasets

Alignments from HISAT2 were imported into the SeqMonk v1.42.0 program (https://github.com/s-andrews/SeqMonk), specifying a minimum mapping quality of 60 to select only uniquely aligned reads. The RNA-seq quantitation pipeline was used to quantitate the number of reads in the cDNA library at the gene level by counting the number of reads which fall into exons of each gene and correcting for the total number of reads in the samples. The final quantitated values were reads per million reads of input and were log2 transformed. To filter the list of quantitated genes for significant DEGs, the intensity difference filter in SeqMonk, which is based on the χ^2^ probe, was used to measure differences in gene expression intensity between any two pairs of control (non-ultrasonicated) and ultrasonicated material at five sampling times (0 h; 24 h; 48 h; 1 w; 4 w). Throughout this paper, we refer to significantly over- and under-expressed DEGs as up- and down-regulated, respectively.

### Functional annotation of significant DEGs

Significantly up- and down-regulated transcribed fragments (transfrags) were selected and blasted against the NCBI database using BlastX-fast in Blast2GO v5.2 (Götz et al. 2008). BlastX-fast was performed against the NCBI nucleotide database (based on the Viridiplanteae) with the minimum E-value score set to 1.0E−06. To assign gene ontology (GO) terms to each annotated sequence, successful blast hits were mapped and annotated using Blast2GO for the significantly up- and down-regulated transfrags with the annotation cut-off threshold set to 55 and the GO level weighting set to 5. After GO mapping, InterProScan was performed and merged to the annotation based on 14 databases (Gene3D, SFLD, SuperFamily, Coils, MobiDBLite, CDD, HAMAP, HMMPanther, HMMPfam, FprintScan, BlastProDom, ProfileScan, HMMTigr, PatternScan) using Blast2GO. KEGG maps (Kanehisa Laboratories; https://www.kegg.jp/kegg/kegg1.html; Kanehisa et al. 2017) were used to search and identify the enzymes and pathways related to DEGs.

### Total RNA isolation for RT-qPCR

Total RNA was purified from the 10 potato samples as three biological replicates each using the Direct-zol™ kit (Zymo Research, Irvine, CA, USA) with TRIzol reagent based on the manufacturer’s protocol. After purifying total RNA, three quality control (QC) methods were applied: (1) microcapillary electrophoresis with an Implen n50 nanophotometer (Implen, Munich, Germany) for preliminary quantification; (2) agarose gel electrophoresis to assess RNA degradation and potential contamination; (3) Agilent Bioanalyzer 2100 system (Agilent Technologies Inc., Santa Clara, CA, USA). For the RT-qPCR analysis, we chose 10 DEGs from the RNA-seq datasets (PGSC0003DMG400038200, PGSC0003DMG400030182, PGSC0003DMG400005112, PGSC0003DMG400015772, PGSC0003DMG400002471, PGSC0003DMG400020069, PGSC0003DMG400027633, PGSC0003DMG401031237, PGSC0003DMG400010170 and PGSC0003DMG402012985) based on the most negative and positive changes in intensity in the SeqMonk logarithmic fold changes (LFC) values. We developed the RT-qPCR primers (Supplementary Table 5) for the chosen DEGs and the two normalising (reference) genes (*GAPDH*, *Actin*) with the CLC Main Workbench 7.9.2 (Qiagen, Hilden, Germany).

### Validation of DEGs by RT-qPCR

Total RNA (120 ng) was reverse transcribed to cDNA using the FIREScript RT cDNA Synthesis MIX (Solis BioDyne, Tartu, Estonia). qPCR was performed with the 5 × HOT FIREPol EvaGreen qPCR Supermix (Solis BioDyne) on the ABI 7300 real-time PCR system (Thermo Fisher Scientific, USA). For RT-qPCR, six biological replicates each from the control and treated samples were used. Both RNA-seq and RT-qPCR showed the same direction (up- or down-regulation) of differential expression and differential expression logarithmic fold change, as estimated by RT-qPCR, and was either > 0.33 or < 1.24. Spearman and Pearson correlation coefficients were calculated using Excel in Microsoft Office 2018 (Microsoft, Redmond, WA, USA).

### Protein analysis by two-dimensional (2-D) gel electrophoresis

Total protein was extracted from ten potato samples (control and treated samples) using the Plant Total Protein Extractor Kit (Sigma-Aldrich, St. Louis, MI, USA). The extracted total plant protein was analyzed by 2-D gel electrophoresis using the Agilent Protein 230 Kit (Agilent, Santa Clara, CA, USA) on an Agilent Bioanalyzer 2100 (Agilent). For the analysis, three biological replicates were used for each sample. We analyzed the total protein samples under reducing (dithiothreitol) and non-reducing (distilled water) conditions.

### Note about methods

The entire methodology (between “Total RNA isolation and removal of rRNA” and “Protein analysis by two-dimensional (2-D) gel electrophoresis”), save for minor differences, has been reported *verbatim* in a paper that recently examined the effect of cutting on explant transcriptomic profile (Teixeira da Silva et al. 2019).

## Results

### Global changes in RNA expression profiles in response to ultrasound-induced stress

**Fig. 1:**
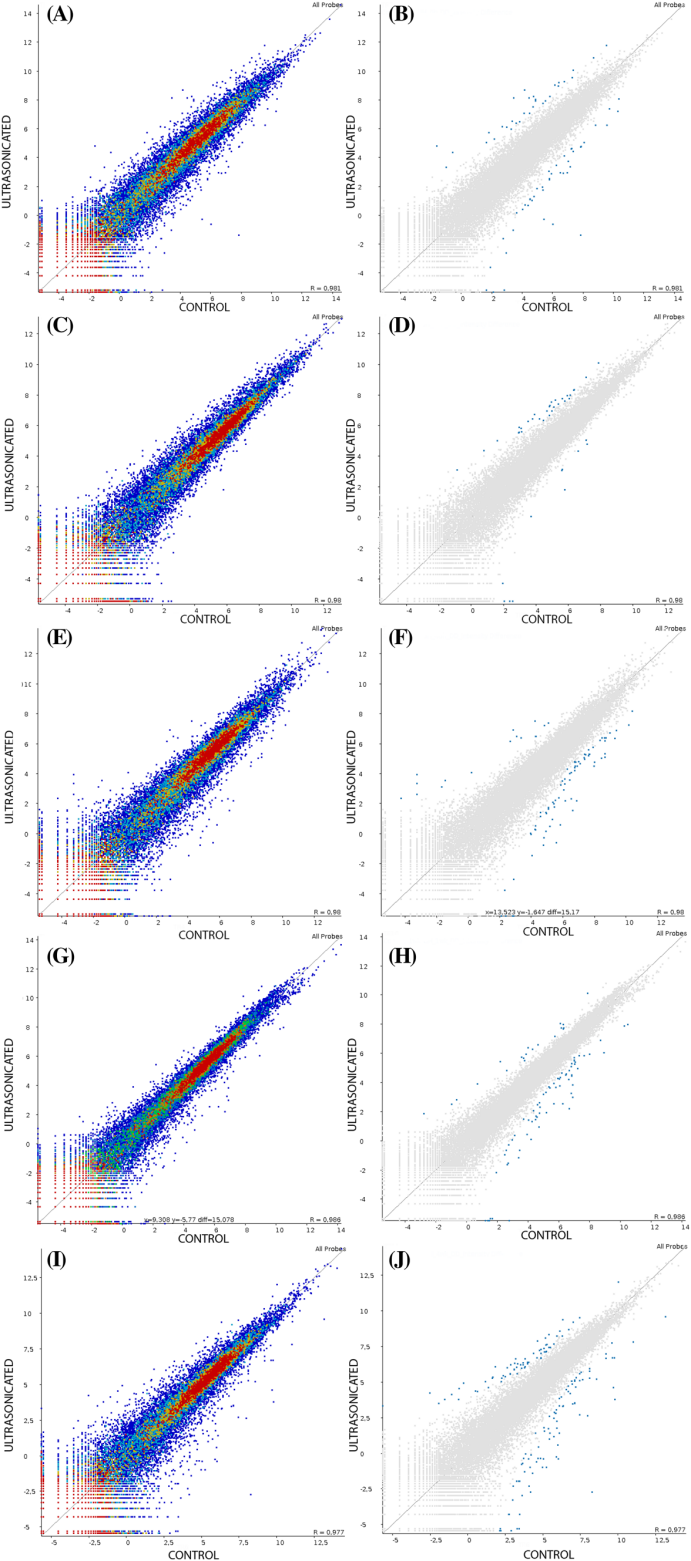
Total number of expressed genes at 0 h (**A**), 24 h (**C**), 48 h (**E**), 1 w (**G**) and 4 w (**I**) and total number of significantly differentially expressed genes at 0 h (**B**), 24 h (**D**), 48 h (**F**), 1 w (**H**) and 4 w (**J**). Scatter plots generated by SeqMonk

When the ultrasonicated explant developed from 0 h to 4 w, 40,430 genes were expressed (Supplementary Table 1) at 0 h, 24 h, 48 h, 1 w and 4 w (Fig. 1A, C, E, G, and I, respectively). There was a wide range (11–70) in the number of significantly up- and down-regulated genes (Table 1; 0 h: Fig. 1B; 24 h: Fig. 1D; 48 h: Fig. 1F; 1 w: Fig. 1H; 4 w: Fig. 1J). Heat maps show the expression intensity of DEGs for the five comparisons between non-ultrasonicated and ultrasonicated material (Supplementary Fig. 1).

**Table 1:** Number of significantly up- and down-regulated genes (based on GO annotation in Blast2GO) in control versus stress (ultrasound (US) for 20 min) treatments at five time intervals after the abiotic stress was applied

Treatment comparisons	# Up-regulated genes	# Down-regulated genes
0 h (control vs. US)	29	34
24 h (control vs. US)	31	11
48 h (control vs. US)	15	64
1 w (control vs. US)	16	70
4 w (control vs. US)	68	69

### RNA-seq/DEG analysis of biological, cellular and molecular processes

When comparing the significantly up- and down-regulated biological, cellular and molecular processes (Supplementary Fig. 2; Supplementary Table 2), a cut-off value of ≥ 50% of DEGs was generally applied in the comparison across all treatments so as to focus on the most important (weighted) processes. Among the 25 biological processes, the vast majority of significantly up- and down-regulated DEGs were limited to five categories (biosynthesis, carbohydrate metabolism, catabolism, cellular protein modification, and response to stress), the latter process displaying significantly up- and down-regulated DEGs in 75% of the comparisons (Supplementary Table 2; Supplementary Results). Among the 16 cellular locations, the vast majority of significantly up- and down-regulated DEGs were limited to three locations (extracellular region, nucleus, and plasma membrane), the extracellular region displaying significantly up- and down-regulated DEGs in 75% of the comparisons (Supplementary Table 2; Supplementary Results). Significantly up- and down-regulated DEGs related to hydrolase activity were observed in 100% of the comparisons (Supplementary Table 2; Supplementary Results).

### What metabolic and cellular functions were up- or down-regulated after US stress?

When comparing control (non-ultrasonicated) and ultrasonicated treatments, the focus was only on significantly up- or down-regulated DEGs related to amino acids, carbohydrates, fatty acids, vitamins, nucleotides, as well as growth, development, and stress.

Compared to the unstressed control at 0 h, US-stressed explants at the same time point showed down-regulation of amino sugar, nucleotide sugar, starch and sucrose metabolism, as well as carotenoid and phenylpropanoid biosynthesis. Purine and thiamine metabolism as well as fatty acid elongation were up-regulated (Supplementary Table 3; Supplementary Fig. 3).

At 24 h, US-stressed explants showed, when compared to unstressed explants in the same time group (Supplementary Table 3; Supplementary Fig. 3), down-regulated alanine, aspartate, glutamate, purine, thiamine and butanoate metabolism. Different genes related to phenylpropanoid biosynthesis were both up- and down-regulated. The metabolism of amino sugar, nucleotide sugar, ascorbate and aldarate, pentose and glucuronate interconversions, as well as flavonoid biosynthesis, were up-regulated.

At 48 h after US (Supplementary Table 3; Supplementary Fig. 3), glycine, serine, threonine, tyrosine and α-linolenic acid metabolism, naphthalene and fatty acid degradation, phenylpropanoid biosynthesis and processes related to carbohydrate metabolism (amino sugar, nucleotide sugar, starch and sucrose metabolism, pentose and glucuronate interconversions) were down-regulated. Only three processes were up-regulated: glycerolipid and riboflavin metabolism, and aminobenzoate degradation.

One week after explants were exposed to US, nine processes each were up-regulated while 10 processes were down-regulated (Supplementary Table 3; Supplementary Fig. 3). The down-regulated processes included pyruvate and amino acid (glycine, serine, threonine, valine, leucine and isoleucine) metabolism, carbohydrate (amino sugar, nucleotide sugar, galactose, starch and sucrose) metabolism, aminobenzoate degradation, and indole alkaloid and carotenoid biosynthesis. The 10 up-regulated processes included amino acid (tryptophan), carbohydrate (galactose, starch and sucrose) and vitamin (ascorbate, aldarate) metabolism, arachidonic acid, inositol phosphate and linoleic acid metabolism, fatty acid and aminobenzoate degradation, and phenylpropanoid biosynthesis.

Four w after US was applied (Supplementary Table 3; Supplementary Fig. 3), five amino acid-related processes were down-regulated (alanine, aspartate, glutamate, glycine, serine, threonine, phenylalanine, tryptophan and tyrosine metabolism) while two were up-regulated (cysteine, methionine and tryptophan metabolism). Different sequences related to purine metabolism were up- and down-regulated. Related to carbohydrate metabolism, pentose and glucuronate interconversions were down-regulated while two processes each related to starch and sucrose metabolism were up- and down-regulated. Fatty acid degradation and glycerolipid metabolism were down-regulated. There was considerable activity related to vitamins: aminobenzoate degradation and ascorbate, aldarate, riboflavin and thiamine metabolism were down-regulated while different sequences related to aminobenzoate degradation and thiamine metabolism were up-regulated. Inositol phosphate metabolism, naphthalene degradation, phenylpropanoid biosynthesis, and taurine and hypotaurine metabolism were down-regulated while two separate sequences for phenylpropanoid biosynthesis were up-regulated.

Greater detail about these processes, including the patterns of expression of enzymes, as well as common sequences in stressed versus unstressed explants over time, may be found in the Supplementary Results.

### Behavior of transcription factors in response to ultrasound-induced stress

When whole 4-w-old plants were ultrasonicated, nine transcription factors (TFs) were significantly up- or down-regulated, including different types of ethylene-responsive TFs, and four probable WRKY TFs (Supplementary Table 4). Only one TF, heat stress TF C-1, was significantly down-regulated immediately (0 h) after ultrasonication. In contrast, 24 h after US treatment, ethylene-responsive TF ERF017 was up-regulated. In 1-w-old plantlets, the ethylene-responsive TF 1B and two different probable WRKY TFs 51 and 70 were down-regulated and no TFs were up-regulated. At the end of the subculture, in 4-w-old plantlets, two ethylene-responsive TFs (4-like and ERF017), as well as probable WRKY TF 31 and TF FAMA, were up-regulated, while the expression intensity of the probable WRKY TF 40 was down-regulated.

### Validity of DEG analysis by RT-qPCR

For RNA-seq data validation, RT-qPCR was used. Using RT-qPCR, false or true positive DEGs could be detected. In the latter case, all of the chosen DEGs were detected as true positive up- or down-regulated DEGs (Supplementary Fig. 4; Supplementary Table 6). The Spearman and Pearson correlation coefficients between SeqMonk LFC and RT-qPCR LFC were both 0.97 (Supplementary Table 6). These high correlation coefficients indicate a high positive correlation between SeqMonk LFC and RT-qPCR LFC.

### Total protein analysis

Total protein 2-D gel electrophoresis also revealed differences between the control and treated samples (Supplementary Fig. 5; Supplementary Table 7).

## Discussion

### Does an ultrasound pulse constitute an abiotic stressor to in vitro potato plantlets?

The purpose of this study was to assess what effect, if any, abiotic stress caused by exposing an in vitro explant to US, would have on the mRNA expression profile (DEG-based enzyme functions) of developing in vitro potato plantlets. To assist us, we used RNA-seq analysis of DEGs that were significantly differentially expressed immediately after exposure to US (0 h), or 24 h, 48 h, 1 w or 4 w after exposure to US. The model plant, potato, was selected since an earlier study (Dobránszki et al. 2017) had already indicated that US induced changes in the protein profile of the in vitro explants and plantlets within a 4-w-long subculture. For example, the activity of ascorbate peroxidase, an enzyme involved in ROS scavenging, increased significantly at 0 h or 24 h after US (Dobránszki et al. 2017). The same enzyme was also up-regulated in response to sound vibration at 500 Hz or more (with a maximum of 3 kHz) for 1 h in *A. thaliana*, showing that high sound frequencies are negative abiotic stressors (Ghosh et al. 2016). However, unlike that study, in our study on potato, cutting the explant and the application of US (35 kHz for 20 min) appeared to constitute only a minor or mild abiotic stress initially (first 24 h), but not during subsequent in vitro growth, even though the sound frequency was at least 10-fold higher than that applied to *A. thaliana* potted plants in the Ghosh et al. (2016) study.

The potentially mild stress-related effect of 35 kHz on potato in vitro growth was previously confirmed by: (1) 100% survival and normal growth of plantlets after a 4-w subculture; (2) a significant increase in shoot length and biomass in 4-w old plantlets relative to control explants (Dobránszki et al. 2017). The fact that the application of this US does not appear to constitute a stress to in vitro potato plants, or might constitute only a mild abiotic stress, is fortified by three important findings in this study: (1) hydrogen peroxide (H_2_O_2_) catabolic-related DEGs were up-regulated from 0 h until 4 w, but genes coding for H_2_O_2_-detoxifying antioxidant systems (APX, POX, CAT and SOD) were not differentially expressed even though they were previously significantly activated (Dobránszki et al. 2017); (2) no DEGs related to the lipid peroxidation product, malondialdehyde, were up- or down-regulated; (3) few DEGs related to calcium signalling were up- or down-regulated (Supplementary Table 1; Supplementary Results), suggesting that some stress may have been induced as a result of explants’ exposure to US, which is typically accompanied by calcium signalling (Zhu 2016).

It is important to note that wounding caused by the application of a pulse of US was not fatal to the plantlets, as demonstrated by the successful regeneration of US-stressed explants into plantlets within 4 w. However, ROS, which are synthesized by NADPH oxidases and peroxidases, and cause an oxidative burst that depletes the pool of antioxidants (Demidchik 2015), were formed in response to wounding in potato in vitro plantlets, activating several low molecular weight antioxidants and increasing the activities of three stress-related enzymes, SOD, GR and APX (Dobránszki et al. 2017). Similarly, in salt-stressed maize plants, H_2_O_2_-detoxifying antioxidants were also activated (Zhang et al. 2017).

### Global DEG profiles in US-stressed potato plantlets

**Fig. 2:**
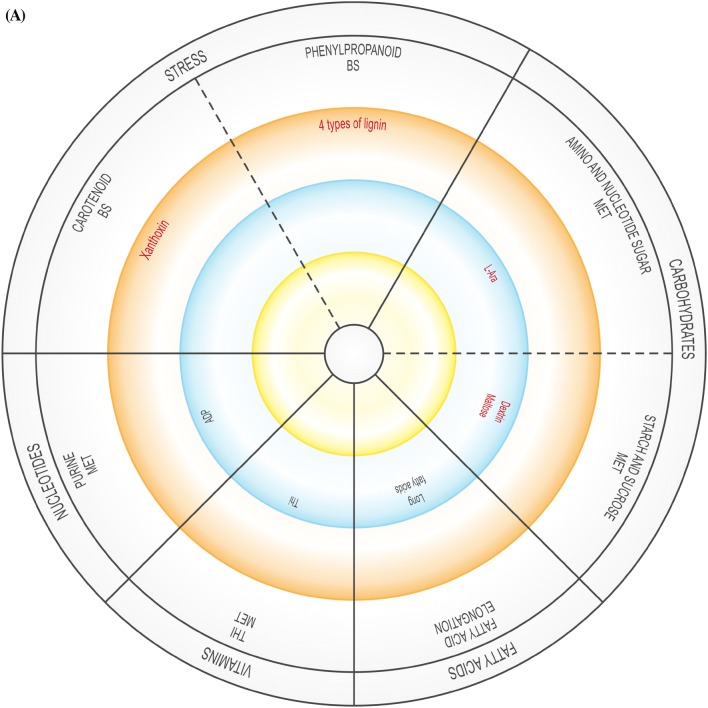

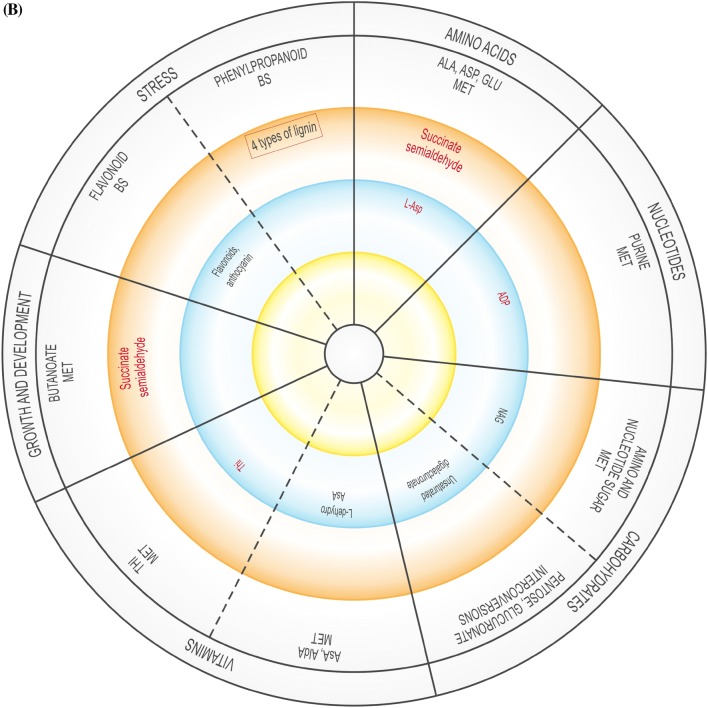

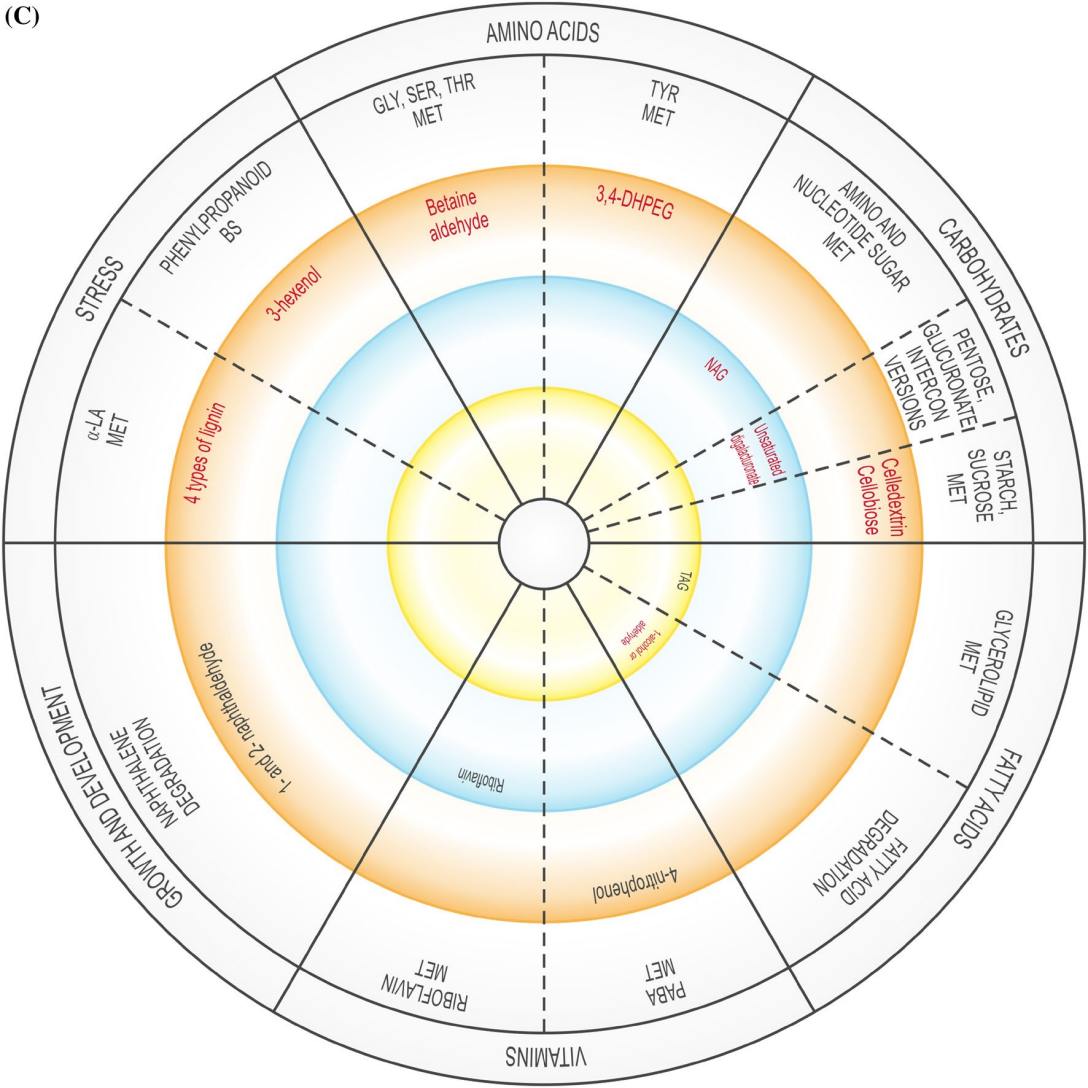

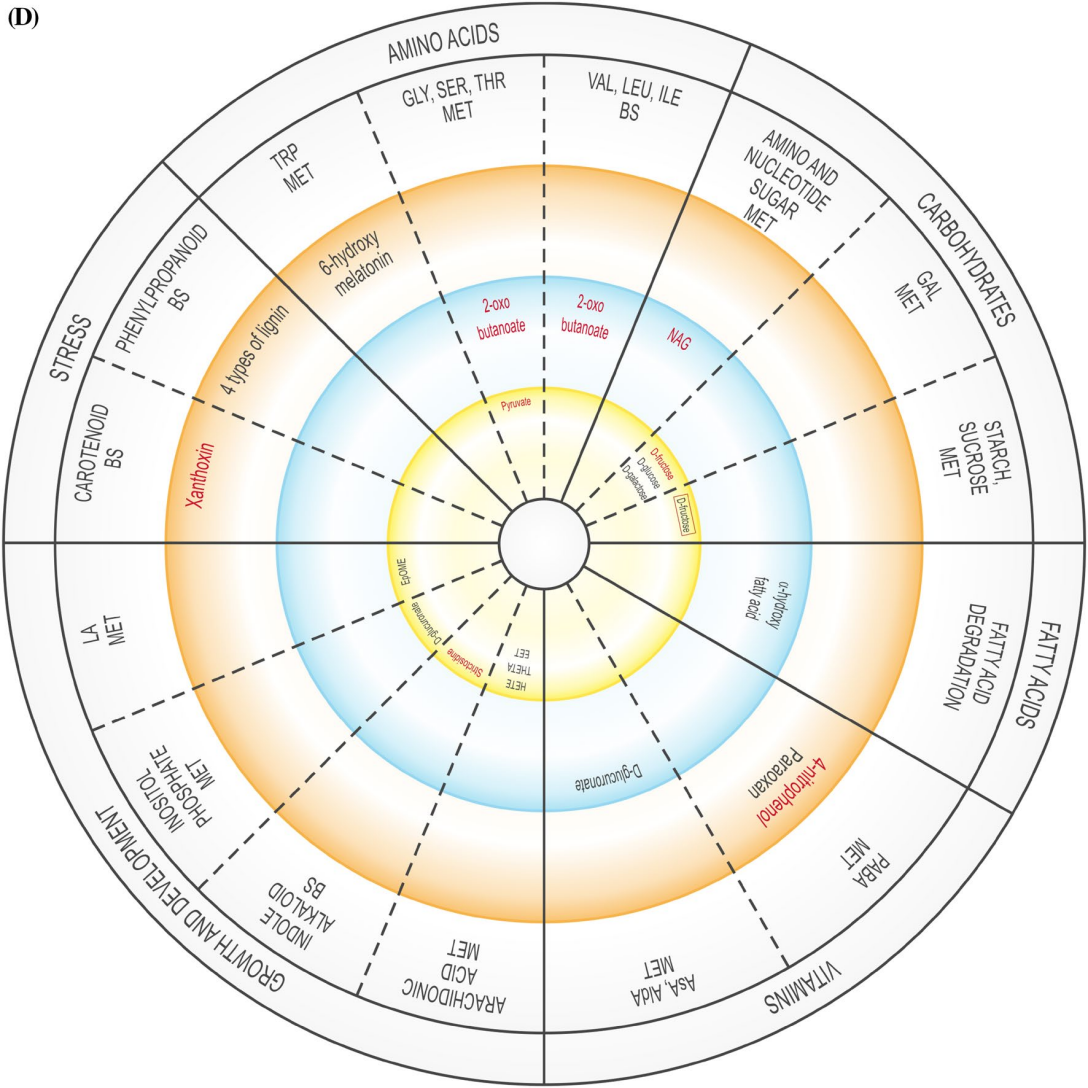

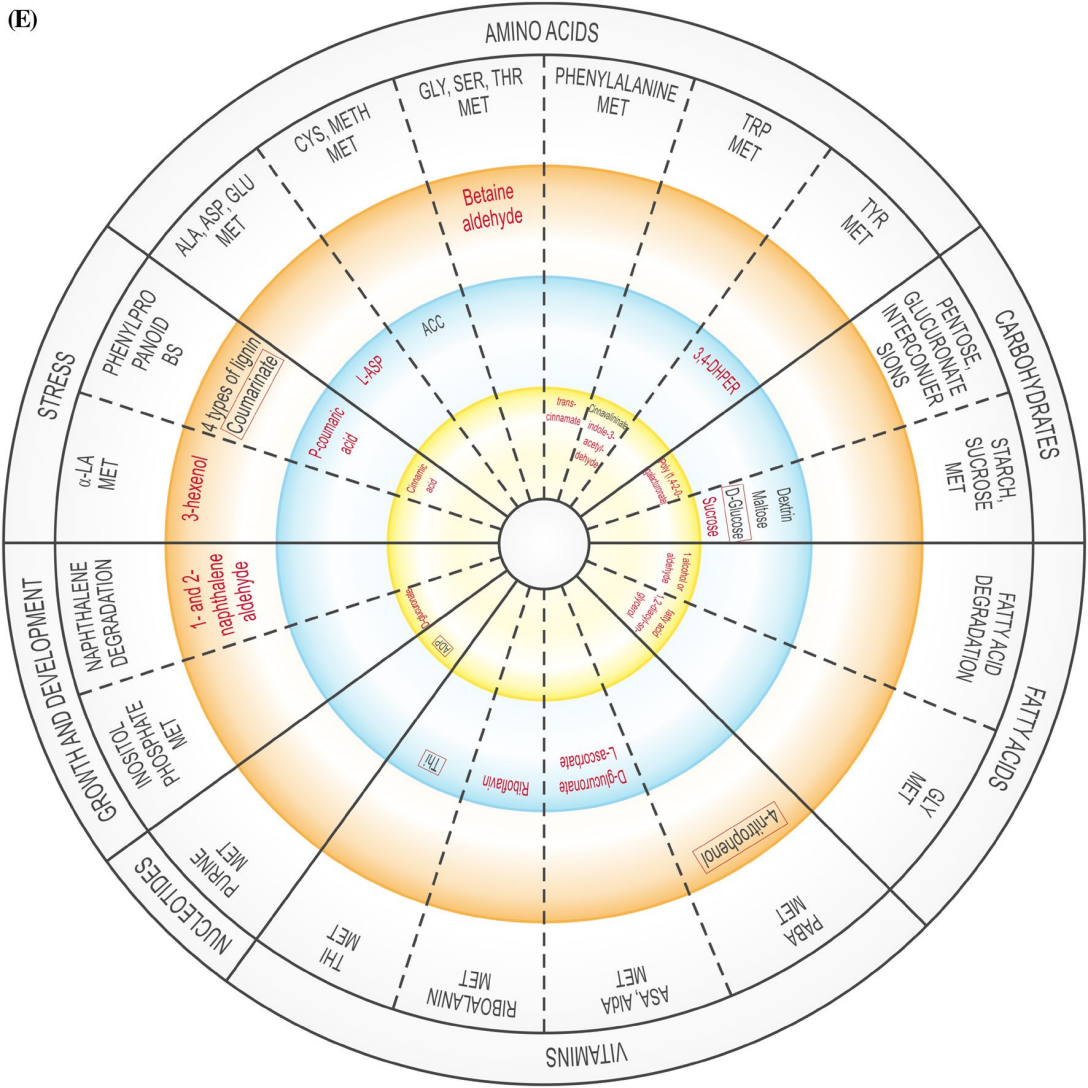
Significantly up- or down-regulated DEGs related to amino acids, carbohydrates, fatty acids, vitamins, nucleotides, as well as growth and development, and stress at 0 h (**A**), 24 h (**B**), 48 h (**C**), 1 w (**D**) and 4 w (**E**). *ACC* 1-aminocyclopropane-1-carboxylic acid, *ADP* adenosine diphosphate, *ALA* alanine, *AldA* aldarate, *l-Asp*l-asparagine, *l-Ara*l-arabinose, *AsA* ascorbate, *ASP* aspartate, *BS* biosynthesis, *CYS* cysteine, *3,4-DHPEG* 3,4-dihydroxiphenylethylene glycol, *EpOME cis* epoxide of linoleic acid, *GAL* galactose, *GLU* glutamate, *GLY* glycine, *ILE* isoleucine, *α-LA* α-linoleic acid, *LEU* leucine, *MET* metabolism, *NAG N*-acetylglucosamine, *PABA* 4-aminobenzoate, *SER* serine, *TAG* triacylglyceride, *THI* thiamine, *THR* threonine, *TRP* tryptophan, *TYR* tyrosine, *VAL* valine. Inner circle (green), process related to growth; middle circle (blue), process related to growth and stress; outer circle (orange), process related to stress. Red text = significantly down-regulated; black text = significantly up-regulated. In **B** and **D**, red box indicates up- and down-regulation

The global DEG profiles derived from RNA-seq analyses provide clues as to whether US is truly a stress, or if it activates stress-related genes. As the plantlets exposed to US developed in vitro, their gene expression profile changed over time, possibly affecting enzymatic, physiological, developmental and stress-related pathways, and several DEGs related to amino acids, carbohydrates, fatty acids, lipid and vitamin metabolism were up- and down-regulated (Supplementary Table 3). The validity of RNA-seq results were validated by RT-qPCR, showing a high correlation coefficient of 0.97 (Supplementary Table 6). These pathways are generally involved in or connected to the plant abiotic stress response or growth. In addition, up- and down-regulated DEGs were related to signalling, regulation, growth and differentiation, as well as to specific stress-related pathways (Supplementary Table 3). Figure 2 is a representative diagram of metabolic processes where DEGS were up- or down-regulated.

### Changes in lipid metabolism and signaling

While butanoate metabolism was down-regulated 48 h after US had ceased to be applied, 1 w after ultrasonication, linoleic acid, arachidonic acid and inositol phosphate metabolism were up-regulated (Supplementary Table 3). Arachidonic acid, which is mobilized by plants in response to oxidative stress and is a lipid messenger that participates in the regulation of signaling enzymes, promotes the expression of stress-responsive genes, and modulates jasmonic acid expression (Fugate et al. 2017; Savchenko et al. 2010). Inositol phosphates, which are related to the inositol signaling pathway (Williams et al. 2015), were up-regulated in 1-w-old potato plantlets in response to US treatment, but not after 4 w. α-Linolenic acid metabolism was down-regulated at 48 h and 1 w after ultrasonication. The release of α-linolenic acid from membrane lipids, as a result of the activity of lipase, modifies membrane fluidity in response to stress (Upchurch 2008). Lipid-transfer protein, which transports phospholipids between cell membranes, was up-regulated at 48 h and 1 w (Supplementary Table 3). Plastid-lipid-associated protein, which is necessary for the development of the plastoglobule, which is rich in lipophilic antioxidants and is involved in resistance to multiple stresses (Singh et al. 2010), was down-regulated at 1 and 4 w.

### Changes in carbohydrate metabolism and/or sink-source

Sugars, the products of photosynthesis that serve as a source for polysaccharide production and energy, also serve as important signals for both plant growth development, or in gene expression, in particular when a plant is exposed to an abiotic stress because they regulate carbohydrate metabolism, although the metabolic event that is affected will depend on the level of the sugar source and sink (Gupta and Kaur 2005). In in vitro potato, carbohydrate metabolism, amino sugar and nucleotide sugar metabolism were down-regulated but galactose metabolism was up-regulated when the US stress had just been applied. At 48 h after US stress, carbohydrate-related processes, amino sugar, nucleotide sugar, starch and sucrose metabolism, pentose metabolism and glucuronate interconversions, were down-regulated while two processes each related to starch and sucrose metabolism were up- and down-regulated. At 1 w, amino sugar, nucleotide sugar, galactose, starch and sucrose metabolism were down-regulated, but galactose, starch and sucrose was up-regulated (Supplementary Table 3). These fluctuations may reveal differences in sinks and/or sources of carbohydrates over the 4-w period, although it is assumed that the exogenously supplied carbohydrate, 3% sucrose (i.e., photomixotrophic culture) in our case, was not a limiting factor because 100% of in vitro control and US-stressed potato plantlets survived on shoot regeneration medium.

### Amino acid metabolism

Amino acids, apart from being building-blocks for proteins, form an integral part of the stress response and developmental regulation in plants. Free amino acids participate directly in the abiotic stress response, such as proline as an osmotic regulator, or GABA, as a signaling molecule (Zeier 2013). Amino acids also have other roles, including the stimulation of some metabolic pathways related to stress response, and adaptation to environmental stimuli and energy production (Zeier 2013). In this study, US increased the production of aspartate after 24 h and 4 w (Supplementary Table 3). Down-regulation of glycine, serine and threonine metabolism after 48 h, 1 w and 4 w might cause lower glycine and threonine production. An increase in phenypropanoid biosynthesis via increased production of 4-hydroxy phenylpyruvate would activate the secondary antioxidant system (Yoshikawa et al. 2018). At 4 w, the up-regulation of cysteine and methionine metabolism may be connected to a change in the redox state of ultrasonicated plantlets compared to non-treated plantlets, as described earlier (Dobránszki et al. 2017). However, the up-regulation of aminocyclopropane carboxylic acid synthase would likely increase ethylene production in the culture vessel, which in our study was not aerated, at the end of the 4-w subculture.

### Vitamin metabolism

Vitamin B degradation frequently occurs in response to abiotic stress (Hanson et al. 2016). Aminobenzoate degradation, ascorbate and aldarate, and thiamine metabolism were up-regulated in response to US at different sampling times (Supplementary Table 3; Fig. 2). Up-regulation of thiamine metabolism might cause the production of Vitamin B1 (thiamine) after 0 h and 24 h (Supplementary Table 3; Fig. 2). The production of thiamine phosphate increased as a result of the up-regulation of nucleoside-triphosphate phosphatase at 0 h and 24 h while up-regulation of nitrophenyl phosphatase may have resulted in an increase in the level of 4-nitrophenol at 48 h, 1 w and 4 w, which would lead to the degradation of benzoate. At 0 h, US caused the up-regulation of nucleoside-triphosphate phosphatase during thiamine metabolism but was down-regulated 24 h after US treatment (Supplementary Table 3). l-Ascorbate oxidase was up-regulated in response to US 24 h after treatment application, which might be connected to the ascorbate–glutathione pathway activated by stress (Hanson et al. 2016). In in vitro potato, change in the ascorbate–glutathione cycle took place 24 h after ultrasonication (Dobránszki et al. 2017). Monodehydroascorbate reductase was stimulated in response to sound vibration in *A. thaliana* (Ghosh et al. 2016). The up-regulation of *myo*-inositol oxygenase 1 w after US increased the production of glucoronate in the ascorbate and aldarate metabolism pathway, but it was down-regulated at 4 w. *Myo*-inositol oxygenase catalyzes the oxidation of D-glucuronate-free inositol while regulation of *myo*-inositol establishes an osmotic balance in stressed plants (Zhang et al. 2017). Riboflavin, which was up-regulated at 48 h and down-regulated 4 w, can enhance drought tolerance, but this depends on the level of ROS (Deng et al. 2014).

### Other stress-related pathways

Carotenoid biosynthesis was down-regulated 48 h and 1 w after ultrasonication, similar to the down-regulation of naphthalene degradation and indole alkaloid biosynthesis. The up-regulation of flavonoid biosynthesis, which might be due to the inactivation of some antioxidant enzymes (Fini et al. 2011), was observed in potato stem explants 24 h after ultrasonication.

### How did transcription factors respond to ultrasound in potato?

WRKY TFs, which were down-regulated at 1 w and 4 w and up-regulated at 4 w, are involved in both stress and development (Phukan et al. 2016; Zhang et al. 2017). Ghosh et al. (2016) found that from 13 TFs that were up-regulated in response to 0.5–3 kHz, six were responsive to mechanostimulus. Immediately after ultrasonication (at 0 h) of single-node explants, the over-regulation of stress-related TFs was not observed, but the heat stress TF C-1 was down-regulated (Supplementary Table 4). In general, heat stress TFs are involved in response to various abiotic stresses and regulate the expression of different stress-responsive genes (Guo et al. 2016). One ERF-type ethylene-responsive TF was overexpressed 24 h after ultrasonication. ERF-type TFs are integrators in hormonal and stress signalling, as well as redox regulation (Müller and Munné-Bosch 2015). Two probable WRKY-type TFs and one ethylene-responsive TF were down-regulated at 1 w after ultrasonication. Two ethylene-responsive (4-like and ERF017) TFs were up-regulated, and this may be connected to an increased level of ethylene in the in vitro growth environment (i.e., in the culture vessel), possibly due to more intensive growth after ultrasonication, as was described earlier (Dobránszki et al. 2017).

## Conclusions

This study attempted to better understand the DEG-based enzyme profiles of in vitro tissue-cultured potato plants that had been exposed to an abiotic stress, namely the application of a US pulse. Many plant abiotic stress-related papers tend to look at a single stressor, such as heat or drought stress, and assess the genomic or transcriptomic expression profile as a binary and static function, noting only whether DEGs are up- or down-regulated. Our experiment offers a third dynamic dimension, namely expression over time, of a unique binary comparison (stressed vs unstressed) at five time intervals (0 h, 24 h, 48 h, 1 w and 4 w after the stress was imposed). If any one of these time points were to be observed in isolation, an interpretation could be offered for each time interval, but collectively, the expression profile over time is neither easy nor simple to interpret. Despite this, we discovered several DEG-based enzyme profiles that matched trends in conventional theoretical plant abiotic stress, but also several unique patterns or even possible exceptions. We conclude that even though several abiotic stress-related DEGs showed increased expression, the US pulse applied in this study did not constitute a fatal stress since in vitro plantlets survived and grew well at 4 w of age, even though variation in growth-related parameters were previously observed (Dobránszki et al. 2017). Additional molecular analyses using RT-qPCR of 10 highly up- or down-regulate DEGs fortified these conclusions by validating the RNA-seq analyses. The application of the same analyses to other plant systems would allow for an understanding if these patterns exist in other plant species. A recent study (Teixeira da Silva et al. 2019) that essentially employed identical methodology as was used in this study, found that, as in this study, several stress-related DEGs were both up- and down-regulated in cut potato explants relative to intact 4 w plantlets.

## Electronic supplementary material

Below is the link to the electronic supplementary material.

Supplementary material 1 (DOCX 51 kb)

Suppl. Figure 1 Heat maps showing expression intensity of significantly up- and down-regulated DEGs. US, ultrasonicated. Heat maps generated by SeqMonk (based on a per-probe normalization). Supplementary material 2 (TIFF 2519 kb)

Suppl. Figure 2 Significantly up- and down-regulated processes (biological, cellular, molecular). Graphs and pie-charts generated by Blast2Go. Supplementary material 3 (TIFF 2398 kb)

Suppl. Figure 3 KEGG maps representing the up- and down-regulated processes outlined in Suppl. Table 3. Copyright permission to use KEGG maps kindly provided by Kanehisa Laboratories. Supplementary material 4 (PDF 1481 kb)

Suppl. Figure 4 Comparisons of logarithmic fold changes (LFC), estimated by RT-qPCR, with LFC, estimated by SeqMonk. Supplementary material 5 (JPEG 93 kb)

Suppl. Figure 5 2-D gel electrophoresis image, created by Agilent Bioanalyzer 2100 (Agilent), for the control and treated samples. Green lines: lower molecular weight marker; purple lines: larger molecular weight marker; blue line: protein fragment. DTT (dithiothreitol): reducing condition; H_2_O (distilled water): non-reducing condition. Supplementary material 6 (JPEG 97 kb)

Suppl. Table 1 Annotation of significantly differentially over- and under-expressed genes (DEGs) based on Blast2Go and SeqMonk at different sampling times during a comparison between non-ultrasonicated and sonicated (35 kHz, 70 W, 20 min) in vitro potato explants.. Supplementary material 7 (XLSX 125 kb)

Suppl. Table 2 Significantly up- and down-regulated processes (biological, cellular, molecular) that were up- or down-regulated in response to ultrasound at five time intervals. Additional details may be found in Suppl. Figure 2. Supplementary material 8 (XLSX 14 kb)

Suppl. Table 3 Significantly up- and down-regulated processes related to amino acids, carbohydrates, fatty acids, vitamins, nucleotides, as well as growth, development, and stress that were up- or down-regulated in response to ultrasound at five time intervals. Additional details may be found in Suppl. Figure 3. Supplementary material 9 (XLSX 11 kb)

Suppl. Table 4 Significantly up- and down-regulated transcription factors that were up- or down-regulated in response to ultrasound at five time intervals. Supplementary material 10 (XLSX 9 kb)

Suppl. Table 5 List of primers used for RT-qPCR. Supplementary material 11 (XLS 24 kb)

Suppl. Table 6 Results of RT-qPCR validation of 10 DEGs. Supplementary material 12 (XLS 45 kb)

Suppl. Table 7 2-D gel electrophoresis results (values in kDa). DTT (dithiothreitol): reducing condition; H_2_O (distilled water): non-reducing condition. Supplementary material 13 (XLS 42 kb)

## Data Availability

The raw Illumina mRNA-seq were submitted to the NCBI and the processed data were deposited under GEO ID GSE123176, BioProject ID PRJNA507769, and SRA ID SRP171630 for the 10 samples: GSM3498049, GSM3498050, GSM3498051, GSM3498052, GSM3498053, GSM3498054, GSM3498055, GSM3498056, GSM3498057, GSM3498058.
