# Supplementary material for: mRNA transcription profile of potato (Solanum tuberosum L.) exposed to ultrasound during different stages of in vitro plantlet development
Source: Plant Mol Biol. 2019 Apr 29;100(4):511–25. doi: 10.1007/s11103-019-00876-0 (PMC6586710; doi:10.1007/s11103-019-00876-0)
Supplement: Supplementary file 1 — Supplementary material 1 (DOCX 51 kb) [file 11103_2019_876_MOESM1_ESM.docx]

**mRNA transcription in potato (*Solanum tuberosum* L.) triggered by ultrasound during different stages of *in vitro* plantlet development**

**Judit Dobránszki^*^ · Norbert Hidvégi · Andrea Gulyás · Jaime A. Teixeira da Silva^*^**

Research Institute of Nyíregyháza, IAREF, University of Debrecen, Nyíregyháza, P.O. Box 12, H-4400, Hungary; * Co-corresponding authors: [jaimetex@yahoo.com](mailto:jaimetex@yahoo.com); [dobranszki@freemail.hu](mailto:dobranszki@freemail.hu)

RNA-seq/DEG analysis of biological, cellular and molecular processes

At 0 h, 8 and 9 biological processes were up- and down-regulated, respectively. Similarly, at 24 h, 8 and 5 processes, at 48 h, 7 and 8 processes, at 1 w, 6 and 8 processes, and at 4 w, 6 and 5 processes were up- and down-regulated, respectively. Four significantly up- and down-regulated DEGs accounted for a high relative percentage value (≥ 30%), namely down-regulated cellular processes at 24 h, up-regulated catabolism at 1 w, down-regulated protein metabolism at 4 w, and up-regulated biosynthetic processes at 4 w (Suppl. Table 2).

When considering the cellular processes, similar to the biological processes at 0 h, 8 and 6 cellular processes were up- and down-regulated, respectively. Similarly, at 24 h, 6 and 3 processes, at 48 h, 5 and 5 processes, at 1 w, 3 and 6 processes, and at 4 w, 3 and 4 processes were up- and down-regulated, respectively. Only one other time interval (1 w after US stress) showed 6 down-regulated cellular processes. Raising the percentage of significance substantially, relative to the biological processes, from ≥ 30% to ≥ 50%, 3 locations had a significantly high proportion of up- and down-regulated DEGs, namely down-regulation in the membrane at 24 and 4 w, and up-regulation in the nucleus at 4 w (Suppl. Table 2).

Among the molecular processes, unlike the biological and cellular processes, at 0 h, only 7 molecular processes were up-regulated while 5 processes were down-regulated. At 24 h, 48 h, 1 w and 4 w after stress was applied, 7, 6, 5 and 7 processes were up-regulated, respectively, while 5, 4, 4 and 7 processes, respectively were down-regulated When considering the same relative level as biological processes (i.e., ≥ 30%), hydrolase activity was significantly down-regulated at all times (0 h, 24 h, 48 h, 1 w, 4 w) but significantly up-regulated at 0 h. No other molecular processes accounted for more than 30% of relative significant expression level (up- or down-regulation) (Suppl. Table 2).

RNA-seq/DEG analysis of metabolic and cellular functions

In this section, a more detailed description is provided about the broad trends observed in Suppl. Table 3, especially with relation to the enzymes.

At 0 h, at least related to purine metabolism, two enzymes, adenylpyrophosphatase (E.C. 3.6.1.3) and phosphatase (E.C. 3.6.1.15), were up-regulated. The same phosphatase was also involved in thiamine metabolism. In phenylpropanoid biosynthesis, lactoperoxidase (E.C. 1.11.1.7) was down-regulated. Dioxygenase (E.C. 1.13.11.51), involved in carotenoid biosynthesis, was down-regulated.

At 24 h, adenylpyrophosphatase and phosphatase in purine and thiamine metabolism were the same as 0 h, but were coded for by different sequences, and were down-regulated. Transaminase (E.C. 2.6.1.96) was down-regulated, affecting butanoate and alanine, aspartate and glutamate metabolism. Lactoperoxidase, encoded for by a different sequence, was down-regulated, similar to at 0 h. Chitinase (ChiC) (E.C. 3.2.1.14) was up-regulated in amino sugar and nucleotide sugar metabolism. Oxidase (E.C. 1.10.3.3) was up-regulated in ascorbate and aldarate metabolism at 24 h.

At 48 h, Lyase (E.C. 4.2.2.2), which was up-regulated at 24 h, was down-regulated during pentose and glucuronate interconversions. Lactoperoxidase, encoded for by two different sequences, was down-regulated, similar to at 0 h and 24 h. Nitrophenyl phosphatase (E.C. 3.1.3.41) was up-regulated during aminobenzoate degradation. Phosphatase was up-regulated in riboflavin metabolism, but was encoded for sequences that differed from all previous time-based comparisons. Sucrose synthase (E.C. 2.4.1.13) was down-regulated in starch and sucrose metabolism. Dehydrogenase (E.C. 1.1.1.1) was down-regulated in and participates in at least seven processes. A different ChiC sequence was down-regulated in amino sugar and nucleotide sugar metabolism, unlike at 24 h, where it was up-regulated.

At 1 w, Monooxygenase (E.C. 1.14.14.1) participated in and was up-regulated during aminobenzoate and fatty acid degradation, as well as in the metabolism of linoleic acid, arachidonic acid and tryptophan. Lactoperoxidase in phenylpropanoid biosynthesis was up-regulated. Nitrophenyl phosphatase was down-regulated during aminobenzoate degradation. Dioxygenase was down-regulated during carotenoid biosynthesis. ChiC, encoded for by a different sequence relative to all other time-based comparisons, was down-regulated in amino sugar and nucleotide sugar metabolism.

At 4 w, lactoperoxidase was up-regulated during phenylpropanoid biosynthesis, where gentobiase (E.C. 3.2.1.21) participated in and was both up- and down-regulated (it was also up- and down-regulated in starch and sucrose metabolism). Ammonia lyase (E.C. 4.3.1.24, 4.3.1.25) was down-regulated in phenylpropanoid biosynthesis but its sequence was different to that of the same enzyme that was down-regulated in glycine, serine and threonine metabolism, and valine, leucine and isoleucine biosynthesis at 1 w. Nitrophenyl phosphatase was both up- and down-regulated during aminobenzoate degradation. Saccharogen amylase (E.C. 3.2.1.2) was up-regulated during starch and sucrose metabolism, and down-regulated at 0 h, but encoded for by a different sequence. Phosphatase (E.C. 3.6.1.15), encoded for by different sequences than all other previous comparisons, was up-regulated during thiamine and purine metabolism, but in the latter group, one of the four sequences coding for phosphatase also encoded adenylpyrophosphatase (E.C. 3.6.1.3). Equilase (E.C. 1.11.1.6), involved in tryptophan, glyoxylate and decarboxylate metabolism, was up-regulated. In purine and thiamine metabolism, one of the three sequences coding for a phosphatase was down-regulated. Oxygenase (E.C. 1.13.99.1) was down-regulated in inositol phosphate, ascorbate and aldarate metabolism. An oxidase (E.C. 1.1.3.8) was down-regulated in ascorbate and aldarate metabolism, but another oxidase (E.C. 1.10.3.3) encoded for by a different sequence was up-regulated at 24 h. The dehydrogenase (E.C. 1.1.1.1) down-regulated in tyrosine, α-linolenic acid, glycine, serine and threonine metabolism, as well as in fatty acid degradation, was encoded for by the same sequence as the dehydrogenase at 48 h. Phosphatase (E.C. 3.1.3.2) in riboflavin metabolism was down-regulated, but up-regulated at 48 h, but the latter phosphatase was encoded for by a separate sequence. A lipase (E.C. 3.1.1.3) was down-regulated in glycerolipid metabolism. Synthase (E.C. 6.3.5.4; glutamine-hydrolyzing) in alanine, aspartate and glutamate metabolism was down-regulated, as occurred at 24 h.

Common sequences in stressed versus unstressed explants over time

When control and US-stressed explants were compared at 0 h and 24 h, two sequences in common were found: replication protein A 70 kDa DNA-binding subunit B-like isoform X1 (up-regulated at 0 h and down-regulated at 24 h), and cysteine protease inhibitor 8-like (up-regulated at 0 and 24 h). When stressed and control explants were compared at 24 and 48 h, some common sequences were up-regulated at 24 h then down-regulated at 48 h (replication protein A 70 kDa DNA-binding subunit B-like isoform X1, TTS protein) while others were first down-regulated at 24 h then up-regulated at 48 h (predicted extensin-1, expansin precursor, probable pectate lyase P18, probable xyloglucan endotransglucosylase/hydrolase protein 16, miraculin-like, L-ascorbate oxidase homolog, 14 kDa proline-rich protein DC2.15-like). When stressed and control explants were compared at 48 h and 1 w, one common sequence was up-regulated over both time intervals (non-specific lipid-transfer protein 2-like), one was initially down-regulated at 48 h and then up-regulated at 1 w (glycine-rich cell wall structural protein 1.8-like), while protein SPIRAL1-like 5 was down-regulated at 48 h and 1 w. When 1 w and 4 w explants were compared (control vs stressed), universal stress protein A-like protein isoform X2 and putative mitochondrial protein were up-regulated at 1 w and then down-regulated at 4 w, BURP domain-containing protein 16-like was up-regulated at both 1 and 4 w, while 11 sequences were down-regulated at both time intervals: two with unknown function, predicted uncharacterized proteins LOC107063386 and LOC104234657, probable protein phosphatase 2C 51, probable plastid-lipid-associated protein 14, chloroplastic, Kunitz-type protease inhibitor precursor, cysteine protease inhibitor 8-like, proteinase inhibitor 1, expansin-like B1, and cysteine proteinase 3.

Hydrogen peroxide (H_2_O_2_) catabolic-related DEGs were activated at all stages of development (2 at 0 h; 1 at 24 h; 3 at 48 h; 2 at 1 w; 1 at 4 w; Suppl. Table 1), no DEGs coding for H_2_O_2_-detoxifying antioxidant systems (ascorbate peroxidase (APX), dehydroascorbate reductase, glutathione peroxidase, glutathione reductase (GR), glutathione-*S*-transferase, monodehydro-ascorbate reductase) were up- or down-regulated, except for a slight expression of a single catalase sequence at 4 w, and a single superoxide dismutase (SOD) sequence encoding SOD chaperone at 0 h (Suppl. Table 1). No DEGs related to the lipid peroxidation product, malondialdehyde, were up- or down-regulated. Some DEGs related to calcium signalling were up- or down-regulated: zero at 0 h, and one each in all the remaining stages of development (Suppl. Table 1). The application of US stress to potato explants resulted in the up- or down-expression of three sequences related to calmodulin while no calmodulin-related DEGs were up- or down-regulated in all subsequent comparisons (Suppl. Table 1).

Specific findings in metabolic pathways

Several detailed findings were observed in specific metabolic pathways, derived from data in Suppl. Table 1 and Suppl. Table 3, which have provided support for the broad summary conclusions drawn in Fig. 2.

Sugar, starch and carbohydrates: At 1 w, an invertase (E.C. 3.2.1.26; β-fructofuranosidase) was up-regulated during starch and sucrose metabolism, and also during galactose metabolism (Suppl. Table 1). When KEGG and non-KEGG functions were searched, no hexokinase was either up- or down-regulated during any stage of development following US treatment, suggesting that a hexose-independent pathway (Gupta and Kaur, 2005) may have been controlling carbohydrate metabolism during this period. In contrast, in this extended search (Suppl. Table 1), several invertases were up- or down-regulated during the 4-w *in vitro* growth period.

Lipids and fatty acids: Phospholipases, which mediate the degradation of phospholipids that serve as signaling molecules during abiotic stress (Agarwal and Zhu, 2005), were observed in US-stressed potato plants^[[1]](#footnote-1)^, specifically after the application of US when one phospholipase was up-regulated at 24 h and 1 w and two phospholipases were down-regulated at 4 w.

Amino acids: Ultrasonication caused down-regulation of alanine, aspartate and glutamate metabolism 24 h after the US treatment and at the end of the subculture, i.e., 4 w, while in later phases (48 h, 1 w or 4 w after US treatment, the metabolism of glycine, serine, threonine, phenylalanine, tryptophan, tyrosine, valine, leucine and isoleucine were down-regulated (Suppl. Table 3).

Vitamins: Up-regulation of nucleoside-triphosphate phosphatase in the aminobenzoate degradation pathway occurred 48 h after ultrasonication, but it was down-regulated after 1 w (Suppl. Table 3).

Other observations

In our study, several DEGs were found in common between time-based treatment comparisons. Replication protein A (RPA) 70 kDa DNA-binding subunit B-like isoform X1, which is involved in DNA recombination, repair and replication (Ishibashi et al. 2001, 2005), was up-regulated at 0 h and down-regulated at 24 h in the nucleus. Ishibashi et al. (2001) found that RPA was regulated in actively dividing tissues of rice such as root tips and young leaves. An expansin precursor was up-regulated at 0 h but down-regulated at 24 h while expansin-like B1 was down-regulated at 1 w and 4 w. Expansin, which is important in plant development and is related to cell wall loosening and to cell extension and other suberization processes that assist in postharvest survival (Lulai, 2007), was up-regulated at 24 h and down-regulated at 48 h. Pectate lyase, which is involved in the soft rotting of plant tissue, was up-regulated at 24 h and down-regulated at 48 h. Protein phosphatase 2C 51 was down-regulated at 1 and 4 w. L-ascorbate oxidase, involved in ascorbate metabolism, was down-regulated at 24 h and up-regulated at 48 h. Glycine-rich cell wall structural protein, involved in cell elongation, was down-regulated at 48 h and up-regulated at 1 w.

Several proteases or proteinases (cysteine protease inhibitor 8-like, proteinase inhibitor 1, cysteine proteinase 3), as well as a Kunitz-type protease inhibitor precursor, were down-regulated at 1 w but up-regulated at 4 w (Suppl. Table 3). Potato possesses a unique group of cysteine proteinase inhibitors (PIs), the PCPIs (Gruden et al., 1997; Fischer et al., 2015). PI activity is activated in response to biotic and abiotic stresses (Mosolov and Valueva, 2005). PCPIs suppressed a form of leaf cysteine proteinase in potato ‘Désirée’ that was found in shoot tips and leaves of *in vitro* plants, localized to cell walls, cytoplasm and vacuoles, suggesting their participation and role in *in vitro* plant growth and development, since it was found that a decrease in the concentration of this cysteine proteinase resulted in shoot stunting and a reduced number of leaves and secondary roots (Pompe-Novak et al., 2002).

Xyloglucan endotransglucosylase/hydrolase (XTH) loosens cell walls (Miedes et al., 2013). XTHs are related to root growth (Vissenberg et al., 2005) and one is even encoded for by a touch gene (*TCH4*) in *A. thaliana* (Braam, 2005). XTH was up-regulated at 24 h but down-regulated at 48 h in US-stressed potato (Suppl. Table 3). More than 10% of *A. thaliana* genes encoding TFs and kinases were up-regulated when stimulated by touch (Lee et al., 2005).

When KEGG functions were searched, only lactoperoxidase was identified, but a search in other database annotations (Suppl. Table 1) revealed that peroxidase (Prx) was up- or down-regulated in all time zones: 1 at 0 h and 24 h; 3 at 48 h and 1 w; 6 at 4 w). Cell wall-based Prxs that are dependent on pH generate H_2_O_2_ (Bolwell and Woftastek, 1997; Cheeseman, 2007) and are associated both with cell elongation as well as restricted growth (Csiszár et al., 2012; Francoz et al., 2015). ROS are controlled by class III Prxs (Karkonen and Kuchitsu, 2015; Minibayeva et al., 2015), which may explain their relatively high numbers as plants grew and elongated between 1 w and 4 w. In potato, one day after tuber wounding, *StPrx* was up-regulated about 240-fold, but the expression of this Prx gene decreased to about 40-fold within one week and reached almost wild-type levels within 2 w onwards after wound induction (Lulai and Neubauer, 2014).

**References**

Agarwal, M., Zhu, J-K. (2005) Integration of abiotic stress signaling pathways. In: Jenks, M.A., Hasegawa, P.M. (eds) Plant Abiotic Stress. Blackwell Publishing, Oxford, UK, pp. 215-247

Bolwell, G.P., Woftastek, P. (1997) Mechanism for the generation of reactive oxygen species in plant defense-broad perspective, Physiol. Mol. Plant Pathol. 51: 347-349.

Braam, J. (2005) In touch: plant responses to mechanical stimuli. New Phytologist 165:373–389

Cheeseman, J.M. (2007) Hydrogen peroxide and plant stress: a challenging relationship. Plant Stress 1: 4-15.

Csiszár, J., Gallé, Á., Horváth, E., Dancsó, P., Gombos, M., Váry, Z., Erdei, L., Györgyey, J., Tari, I. (2012) Different peroxidase activities and expression of abiotic stress-related peroxidases in apical root segments of wheat genotypes with different drought stress tolerance under osmotic stress. Plant Physiology and Biochemistry 52: 119-129

Fischer, M., Kuckenberg, M., Kastilan, R., Muth, J., Gebhardt, C. (2015) Novel *in vitro* inhibitory functions of potato tuber proteinaceous inhibitors. Mol Genet Genomics 290:387–398

Francoz, E., Ranocha, P., Nguyen-Kim, H., Jamet, E., Burlat, V., Dunand, C. (2015) Roles of cell wall peroxidases in plant development. Phytochemistry 112: 15-21

Gruden, K., Štrukelj, B., Ravnikar, M., Poljšak-Prijatelj, M., Mavrič, I., Brzin, J., Pungerčar J., Kregar, I. (1997) Potato cysteine proteinase inhibitor gene family: molecular cloning, characterisation and immunocytochemical localisation studies. *Plant Molecular Biology* 34: 317–323

Gupta AK, Kaur N. 2005. Sugar signalling and gene expression in relation to carbohydrate metabolism under abiotic stresses in plants. Journal of Biosciences 30(5):761–776

Ishibashi, T., Koga, A., Yamamoto, T., Uchiyama, Y., Mori, Y., Hashimoto, J., Kimura, S., Sakaguchi, K. (2005) Two types of replication protein A in seed plants. Characterization of their functions *in vitro* and *in vivo*. FEBS Journal 272: 3270–3281

Ishibashi, T., Kimura, S., Furukawa, T., Hatanaka, M., Hashimoto, J., Sakaguchi, K. (2001) Two types of replication protein A 70 kDa subunit in rice, *Oryza sativa*: molecular cloning, characterization, and cellular & tissue distribution. Gene 272: 335–343

Karkonen, A., Kuchitsu, K. (2015) Reactive oxygen species in cell wall metabolism and development in plants. Phytochemistry 112: 22-32

Lee D, Polisensky DH, Braam J. (2005). Genome wide identification of touch- and darkness-regulated Arabidopsis genes: a focus on calmodulin-like and *XTH* genes. *New Phytologist* 165:429-444

Lulai, E.C. (2007) Skin-set, wound healing, and related defects. In: Vreugdenhil, D. (ed) Potato Biology and Biotechnology: Advances and Perspectives, Elsevier B.V., The Netherlands, pp. 471-500

Lulai, E.C., Neubauer, J.D. (2014) Wound-induced suberization genes are differentially expressed, spatially and temporally, during closing layer and wound periderm formation. Postharvest Biology and Technology 90: 24–33

Miedes, E., Suslov, D., Vandenbussche, F., Kenobi, K., Ivakov, A., Van Der Straeten, D., Lorences, E.P., Mellerowicz, E.J., Verbelen, J-P., Vissenberg, K. (2013) Xyloglucan endotransglucosylase/hydrolase (XTH) overexpression affects growth and cell wall mechanics in etiolated *Arabidopsis* hypocotyls. *Journal of Experimental Botany* 64(8): 2481–2497

Minibayeva, F., Beckett, R.P., Kranner, I. (2015) Roles of apoplastic peroxidases in plant response to wounding. Phytochemistry 112: 122-129

Mosolov VV, Valueva TA (2005) Proteinase inhibitors and their function in plants: a review. Appl Biochem Microbiol 41:227–246

Pompe-Novak, M., Poljsak-Prijatelj M., Popovic T., Strukelj B., Ravnikar M, (2002) The impact of potato cysteine proteinases in plant growth and development. *Physiol. Mol. Plant Pathol.*, 60(1): 71–78.

Vissenberg K, Oyama M, Osato Y, Yokoyama R, Verbelen J-P, Nishitani K. 2005. Differential expression of *AtXTH17*, *AtXTH18*, *AtXTH19* and *AtXTH20* genes in *Arabidopsis* roots. Physiological roles in specification in cell wall construction. *Plant and Cell Physiology* 46:192–200.

1. Unlike all other classifications, where KEGG descriptions were used, in this case, the Interproname classification was used. [↑](#footnote-ref-1)
