# Supplementary figures and images for: mRNA transcription profile of potato (Solanum tuberosum L.) exposed to ultrasound during different stages of in vitro plantlet development
Source: Plant Mol Biol. 2019 Apr 29;100(4):511–25. doi: 10.1007/s11103-019-00876-0 (PMC6586710; doi:10.1007/s11103-019-00876-0)

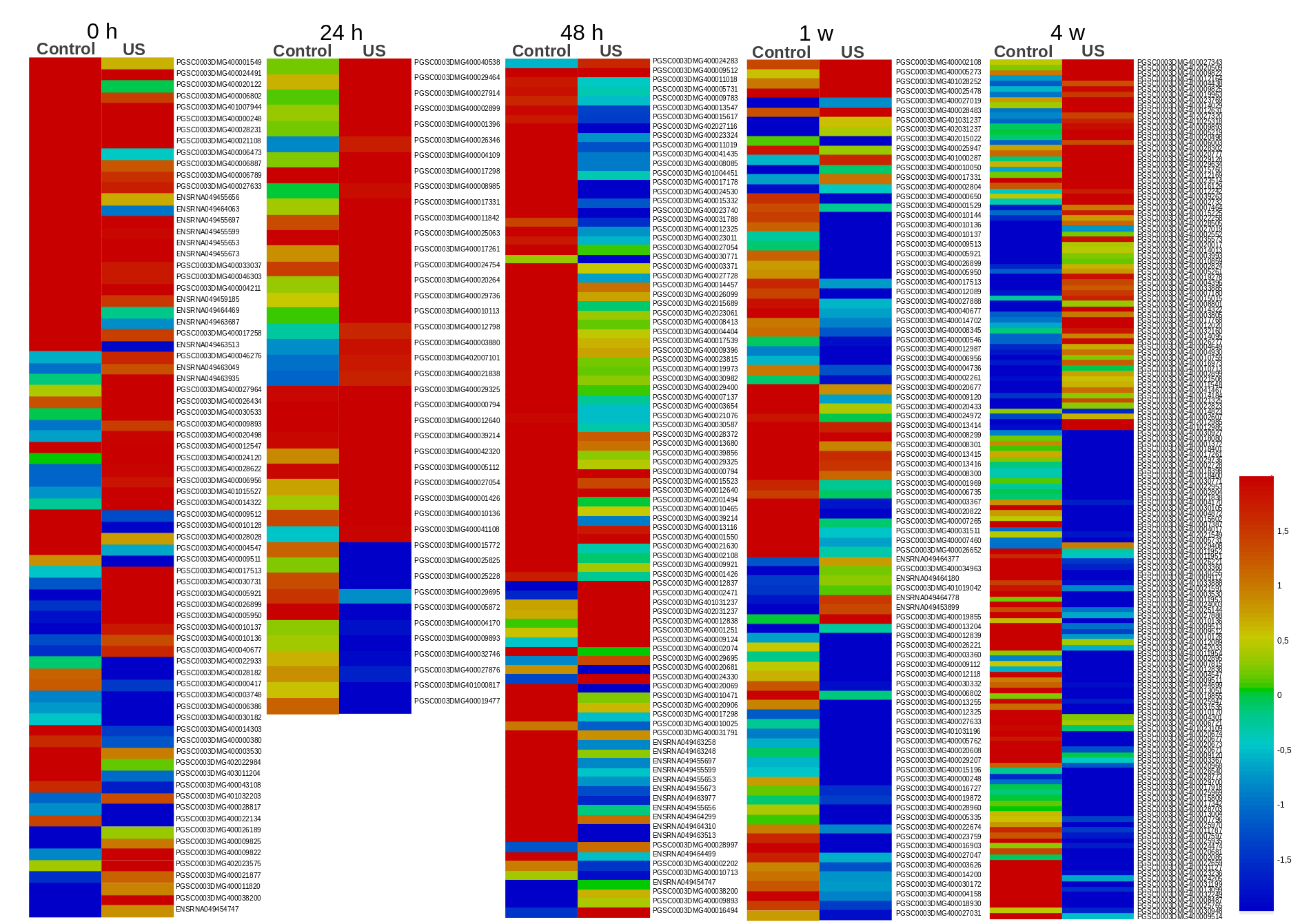

Supplement: Supplementary file 2 — Suppl. Figure 1 Heat maps showing expression intensity of significantly up- and down-regulated DEGs. US, ultrasonicated. Heat maps generated by SeqMonk (based on a per-probe normalization). Supplementary material 2 (TIFF 2519 kb) [file 11103_2019_876_MOESM2_ESM.tif]

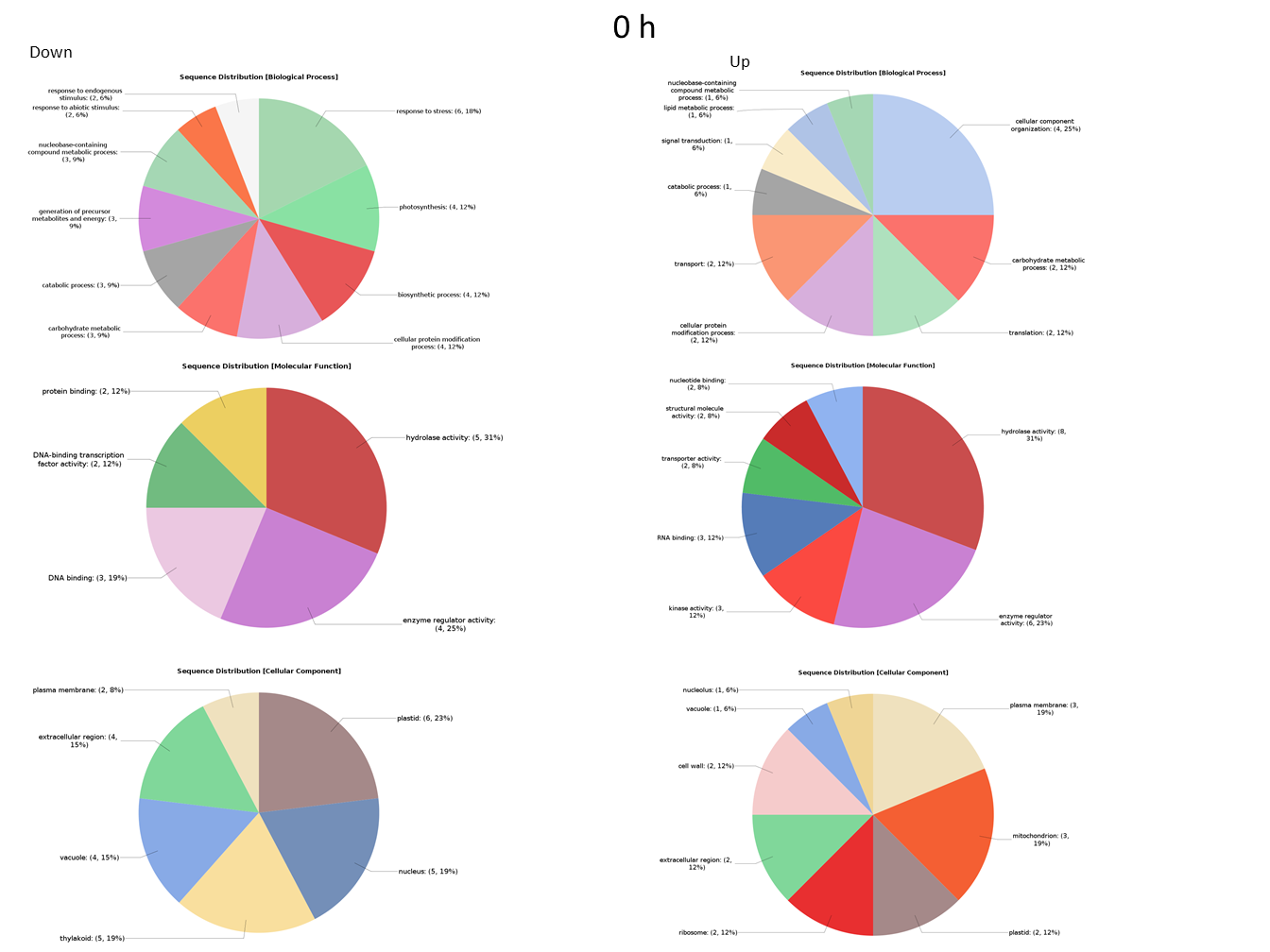

Supplement: Supplementary file 3 — Suppl. Figure 2 Significantly up- and down-regulated processes (biological, cellular, molecular). Graphs and pie-charts generated by Blast2Go. Supplementary material 3 (TIFF 2398 kb) [file 11103_2019_876_MOESM3_ESM.tiff]

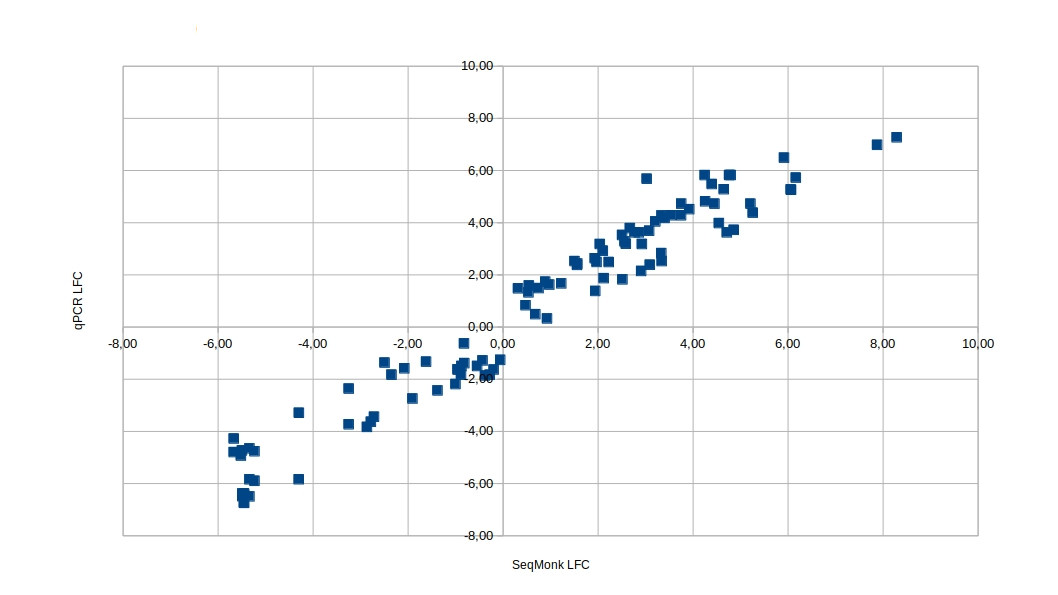

Supplement: Supplementary file 5 — Suppl. Figure 4 Comparisons of logarithmic fold changes (LFC), estimated by RT-qPCR, with LFC, estimated by SeqMonk. Supplementary material 5 (JPEG 93 kb) [file 11103_2019_876_MOESM5_ESM.jpg]

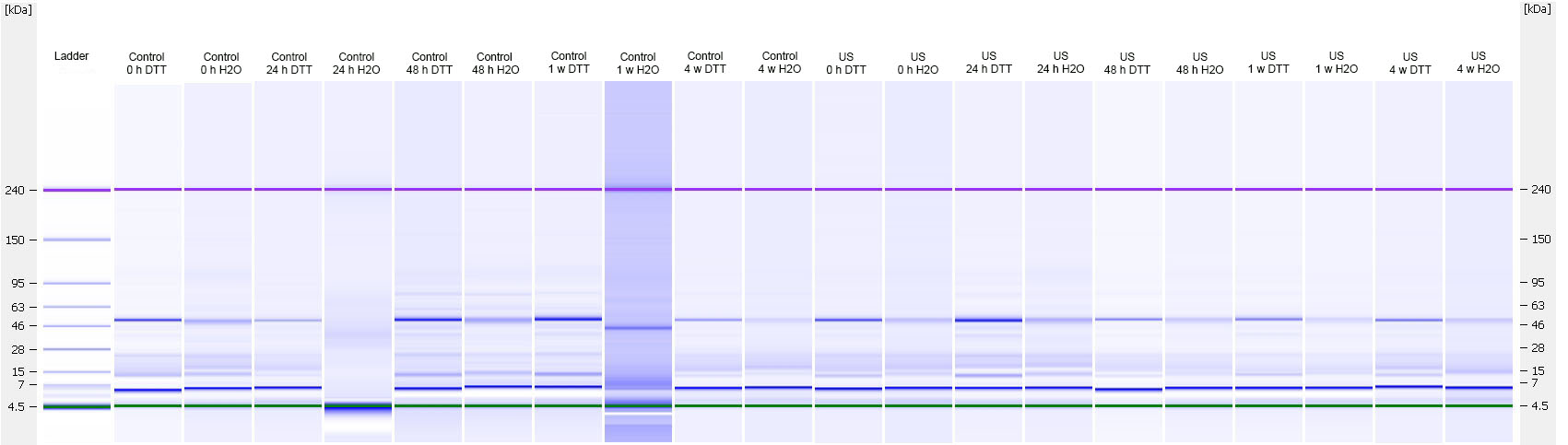

Supplement: Supplementary file 6 — Suppl. Figure 5 2-D gel electrophoresis image, created by Agilent Bioanalyzer 2100 (Agilent), for the control and treated samples. Green lines: lower molecular weight marker; purple lines: larger molecular weight marker; blue line: protein fragment. DTT (dithiothreitol): reducing condition; H2O (distilled water): non-reducing condition. Supplementary material 6 (JPEG 97 kb) [file 11103_2019_876_MOESM6_ESM.jpg]
