# Supplementary material for: mRNA transcription profile of potato (Solanum tuberosum L.) exposed to ultrasound during different stages of in vitro plantlet development
Source: Plant Mol Biol. 2019 Apr 29;100(4):511–25. doi: 10.1007/s11103-019-00876-0 (PMC6586710; doi:10.1007/s11103-019-00876-0)
Supplement: Supplementary file 4 — Suppl. Figure 3 KEGG maps representing the up- and down-regulated processes outlined in Suppl. Table 3. Copyright permission to use KEGG maps kindly provided by Kanehisa Laboratories. Supplementary material 4 (PDF 1481 kb) [file 11103_2019_876_MOESM4_ESM.pdf]

A

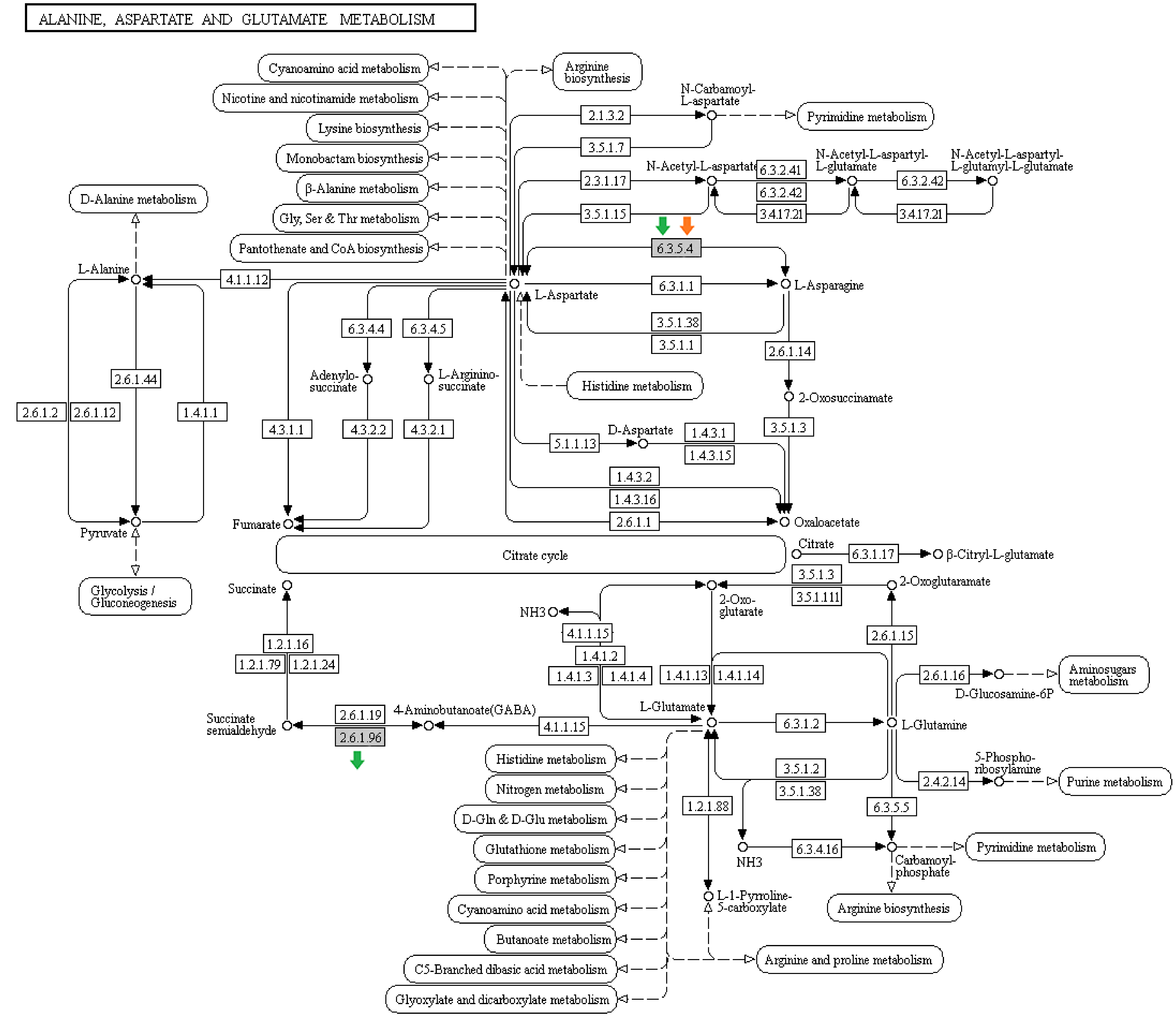

00250 4/9/18
(c) Kanehisa Laboratories

B

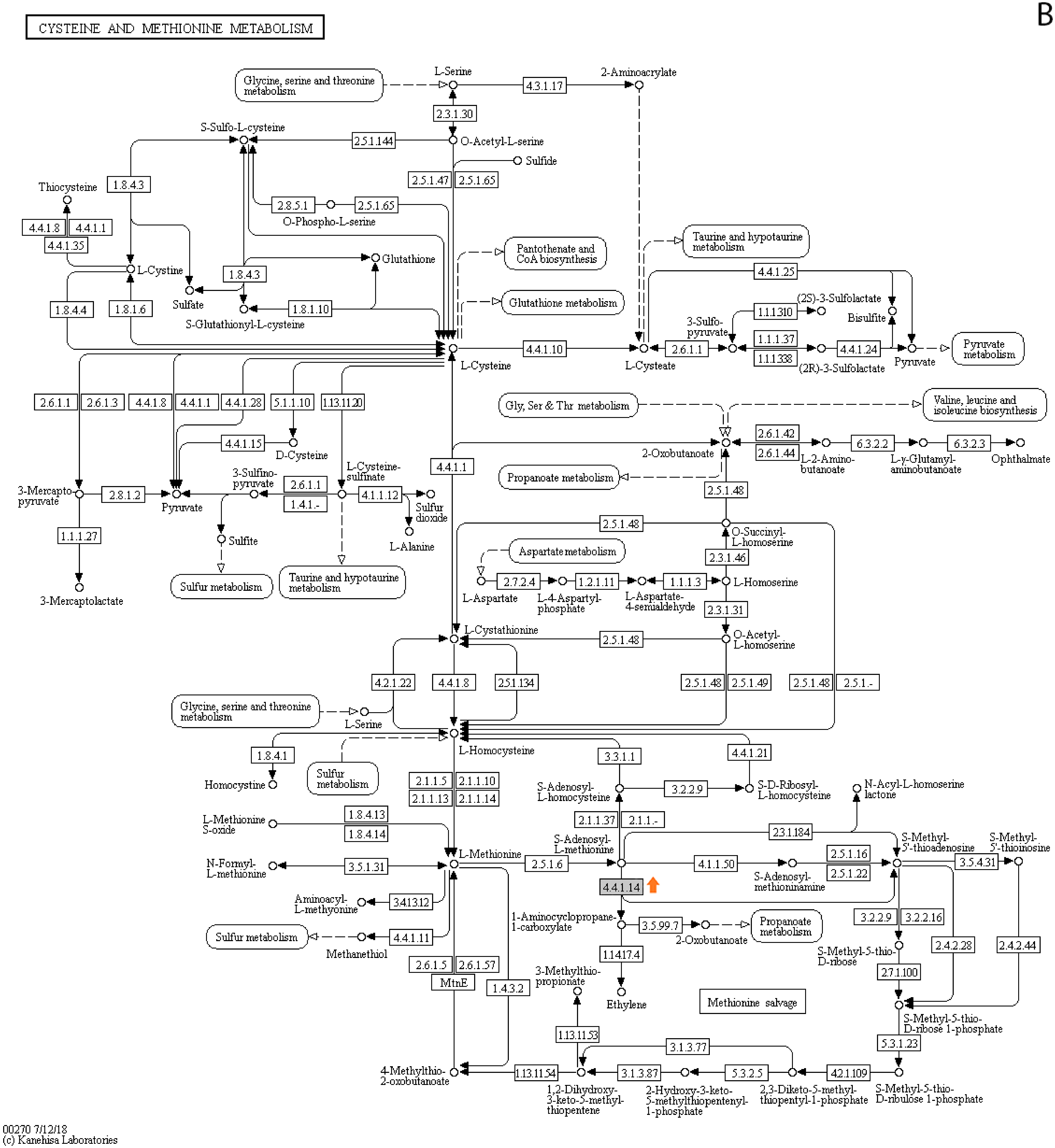
CYSTEINE AND METHIONINE METABOLISM
Glycine, serine and threonine metabolism
L-Serine
4.3.1.17
2-Aminoacrylate
2.3.1.30
S-Sulfo-L-cysteine
2.5.1.144
O-Acetyl-L-serine
Sulfide
2.5.1.47
2.5.1.65
1.8.4.3
Thiocysteine
2.8.5.1
2.5.1.65
O-Phospho-L-serine
4.4.1.8
4.4.1.1
4.4.1.35
Pantothenate and CoA biosynthesis
Taurine and hypotaurine metabolism
Glutathione
L-Cystine
1.8.4.3
4.4.1.25
Sulfate
Glutathione metabolism
1.8.4.4
1.8.1.6
1.8.1.10
S-Glutathionyl-L-cysteine
(2S)-3-Sulfolactate
1.1.1.310
Bisulfite
3-Sulfo-pyruvate
1.1.1.37
4.4.1.10
2.6.1.1
4.4.1.24
Pyruvate
Pyruvate metabolism
1.1.1.338
L-Cysteine
L-Cysteate
(2R)-3-Sulfolactate
2.6.1.1
2.6.1.3
4.4.1.8
4.4.1.1
4.4.1.28
5.1.1.10
1.13.11.20
Gly, Ser & Thr metabolism
Valine, leucine and isoleucine biosynthesis
2.6.1.42
6.3.2.2
6.3.2.3
4.4.1.15
D-Cysteine
2-Oxobutanoate
2.6.1.44
L-2-Amino-butanoate
L-γ-Glutamyl-aminobutanoate
Ophthalmate
4.4.1.1
3-Sulfino-pyruvate
L-Cysteine-sulfinate
Propanoate metabolism
3-Mercapto-pyruvate
2.8.1.2
Pyruvate
2.6.1.1
1.4.1.-
4.1.1.12
Sulfur dioxide
2.5.1.48
1.1.1.27
Sulfite
L-Alanine
2.5.1.48
O-Succinyl-L-homoserine
Aspartate metabolism
2.3.1.46
Sulfur metabolism
Taurine and hypotaurine metabolism
2.7.2.4
1.2.1.11
1.1.1.3
L-Homoserine
3-Mercaptolactate
L-Aspartate
L-4-Aspartyl-phosphate
L-Aspartate-4-semialdehyde
2.3.1.31
L-Cystathionine
2.5.1.48
O-Acetyl-L-homoserine
4.2.1.22
4.4.1.8
2.5.1.134
2.5.1.48
2.5.1.49
2.5.1.48
2.5.1.-
Glycine, serine and threonine metabolism
L-Serine
L-Homocysteine
1.8.4.1
3.3.1.1
4.4.1.21
Sulfur metabolism
Homocystine
2.1.1.5
2.1.1.10
2.1.1.13
2.1.1.14
S-Adenosyl-L-homocysteine
3.2.2.9
S-D-Ribosyl-L-homocysteine
N-Acyl-L-homoserine lactone
L-Methionine S-oxide
1.8.4.13
1.8.4.14
2.1.1.37
2.1.1.-
2.3.1.184
S-Adenosyl-L-methionine
S-Methyl-5'-thioadenosine
S-Methyl-5'-thioinosine
L-Methionine
N-Formyl-L-methionine
3.5.1.31
2.5.1.6
4.1.1.50
2.5.1.16
3.5.4.31
2.5.1.22
S-Adenosyl-methioninamine
4.4.1.14
Aminoacyl-L-methyonine
3.4.13.12
1-Aminocyclopropane-1-carboxylate
3.5.99.7
Propanoate metabolism
3.2.2.9
3.2.2.16
2-Oxobutanoate
Sulfur metabolism
4.4.1.11
Methanethiol
1.14.17.4
S-Methyl-5-thio-D-ribose
2.4.2.28
2.4.2.44
2.6.1.5
2.6.1.57
2.7.1.100
MtnE
1.4.3.2
3-Methylthio-propionate
Ethylene
Methionine salvage
S-Methyl-5-thio-D-ribose 1-phosphate
1.13.11.53
3.1.3.77
5.3.1.23
4-Methylthio-2-oxobutanoate
1.13.11.54
3.1.3.87
5.3.2.5
4.2.1.109
1,2-Dihydroxy-3-keto-5-methyl-thiopentene
2-Hydroxy-3-keto-5-methylthiopentenyl-1-phosphate
2,3-Diketo-5-methyl-thiopentyl-1-phosphate
S-Methyl-5-thio-D-ribulose 1-phosphate
00270 7/12/18
(c) Kanehisa Laboratories

C

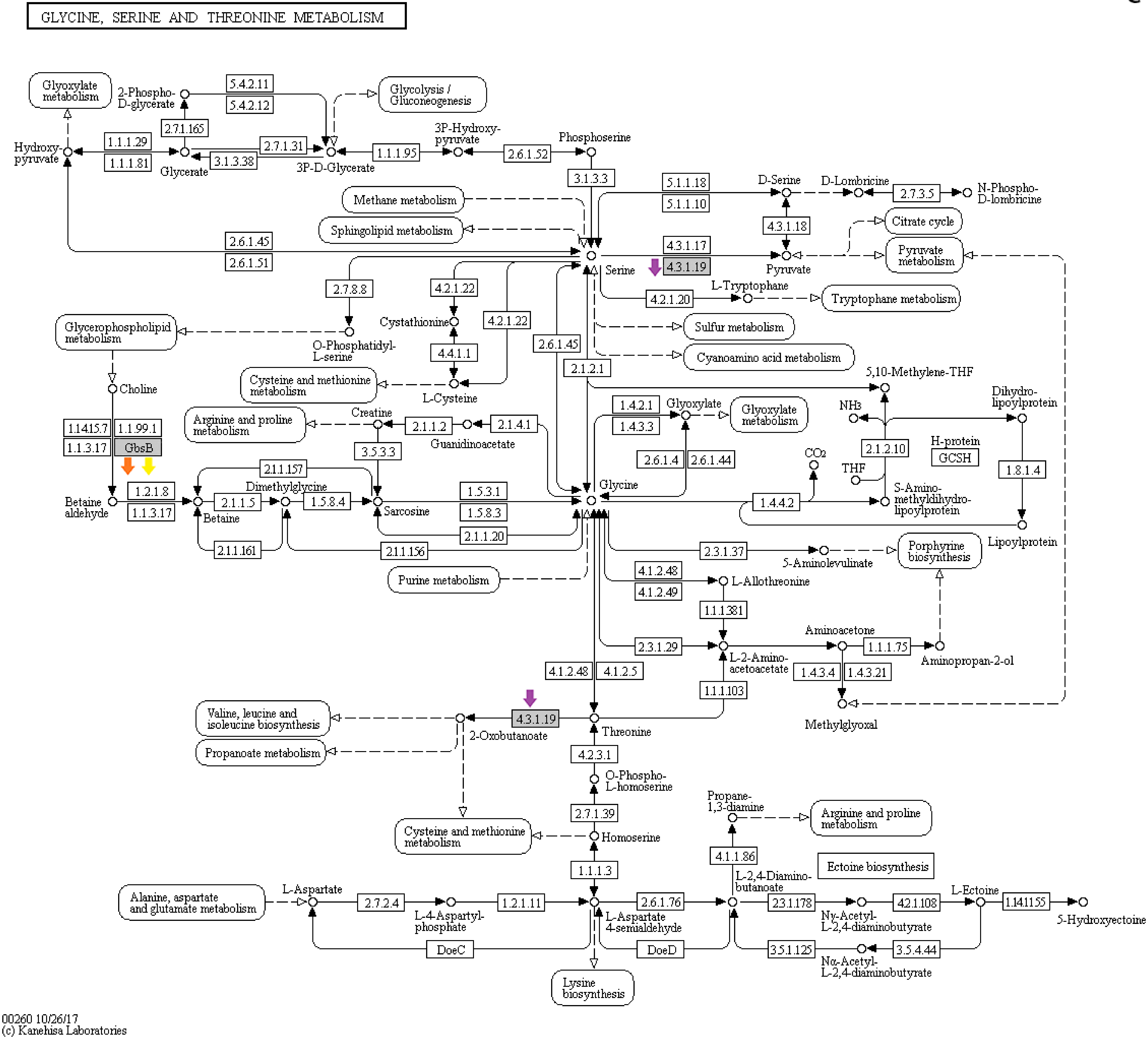
GLYCINE, SERINE AND THREONINE METABOLISM
Glyoxylate metabolism
2-Phospho-D-glycerate
5.4.2.11
5.4.2.12
Glycolysis / Gluconeogenesis
2.7.1.165
Hydroxy-pyruvate
1.1.1.29
1.1.1.81
Glycerate
2.7.1.31
3.1.3.38
3P-D-Glycerate
1.1.1.95
3P-Hydroxy-pyruvate
2.6.1.52
Phosphoserine
3.1.3.3
Methane metabolism
Sphingolipid metabolism
5.1.1.18
5.1.1.10
D-Serine
D-Lombricine
2.7.3.5
N-Phospho-D-lombricine
4.3.1.18
Citrate cycle
2.6.1.45
2.6.1.51
Serine
4.3.1.17
4.3.1.19
Pyruvate
Pyruvate metabolism
2.7.8.8
4.2.1.22
4.2.1.20
L-Tryptophane
Tryptophane metabolism
Glycerophospholipid metabolism
O-Phosphatidyl-L-serine
Cystathionine
4.2.1.22
2.6.1.45
Sulfur metabolism
4.4.1.1
Cyanoamino acid metabolism
2.1.2.1
Cysteine and methionine metabolism
L-Cysteine
5,10-Methylene-THF
Choline
Arginine and proline metabolism
Creatine
2.1.1.2
2.1.4.1
1.4.2.1
Glyoxylate
Glyoxylate metabolism
NH3
Dihydro-lipoylprotein
1.14.15.7
1.1.99.1
Guanidinoacetate
1.4.3.3
1.1.3.17
GbsB
3.5.3.3
2.6.1.4
2.6.1.44
CO2
2.1.2.10
H-protein
GCSH
THF
2.1.1.157
1.8.1.4
1.2.1.8
Dimethylglycine
Glycine
Betaine aldehyde
2.1.1.5
1.5.8.4
1.5.3.1
1.4.4.2
S-Amino-methyldihydro-lipoylprotein
1.1.3.17
Betaine
Sarcosine
1.5.8.3
2.1.1.20
Lipoylprotein
2.1.1.161
2.1.1.156
2.3.1.37
5-Aminolevulinate
Porphyrine biosynthesis
Purine metabolism
4.1.2.48
4.1.2.49
L-Allothreonine
1.1.1.381
Aminoacetone
2.3.1.29
1.1.1.75
L-2-Amino-acetoacetate
Aminopropan-2-ol
4.1.2.48
4.1.2.5
1.4.3.4
1.4.3.21
1.1.1.103
Valine, leucine and isoleucine biosynthesis
4.3.1.19
Methylglyoxal
2-Oxobutanoate
Threonine
Propanoate metabolism
4.2.3.1
O-Phospho-L-homoserine
Propane-1,3-diamine
2.7.1.39
Arginine and proline metabolism
Cysteine and methionine metabolism
Homoserine
4.1.1.86
Ectoine biosynthesis
1.1.1.3
L-2,4-Diamino-butanoate
L-Aspartate
L-Ectoine
Alanine, aspartate and glutamate metabolism
2.7.2.4
1.2.1.11
2.6.1.76
2.3.1.178
4.2.1.108
1.14.11.55
L-4-Aspartyl-phosphate
L-Aspartate 4-semialdehyde
Nγ-Acetyl-L-2,4-diaminobutyrate
5-Hydroxyectoine
DoeC
DoeD
3.5.1.125
3.5.4.44
Nα-Acetyl-L-2,4-diaminobutyrate
Lysine biosynthesis
00260 10/26/17
(c) Kanehisa Laboratories

D

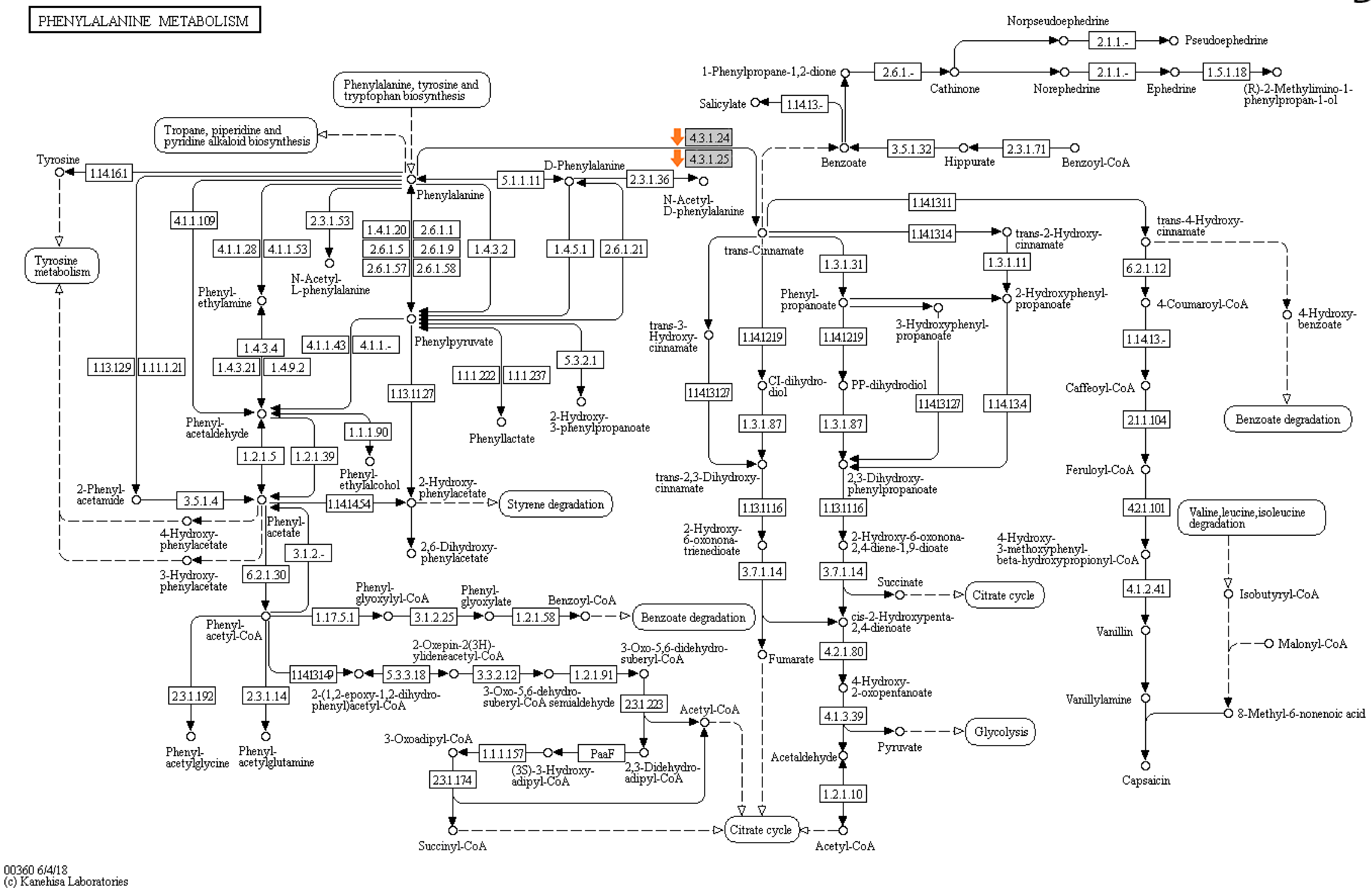
PHENYLALANINE METABOLISM
Phenylalanine, tyrosine and tryptophan biosynthesis
Tropane, piperidine and pyridine alkaloid biosynthesis
Tyrosine
1.14.16.1
Tyrosine metabolism
Phenylalanine
D-Phenylalanine
5.1.1.11
2.3.1.36
N-Acetyl-D-phenylalanine
4.3.1.24
4.3.1.25
4.1.1.109
4.1.1.28
4.1.1.53
2.3.1.53
N-Acetyl-L-phenylalanine
1.4.1.20
2.6.1.1
2.6.1.5
2.6.1.9
2.6.1.57
2.6.1.58
1.4.3.2
1.4.5.1
2.6.1.21
Phenylethylamine
Phenylpyruvate
1.4.3.4
4.1.1.43
4.1.1.-
1.13.12.9
1.11.1.21
1.4.3.21
1.4.9.2
1.13.11.27
1.1.1.222
1.1.1.237
5.3.2.1
2-Hydroxy-3-phenylpropanoate
Phenyllactate
Phenylacetaldehyde
1.1.1.90
Phenylethylalcohol
1.2.1.5
1.2.1.39
2-Phenylacetamide
3.5.1.4
Phenylacetate
1.14.14.54
2-Hydroxyphenylacetate
Styrene degradation
2,6-Dihydroxyphenylacetate
4-Hydroxyphenylacetate
3-Hydroxyphenylacetate
3.1.2.-
6.2.1.30
Phenylacetyl-CoA
1.17.5.1
Phenylglyoxylyl-CoA
3.1.2.25
Phenylglyoxylate
1.2.1.58
Benzoyl-CoA
Benzoate degradation
1.14.13.149
5.3.3.18
2-(1,2-epoxy-1,2-dihydrophenyl)acetyl-CoA
2-Oxepin-2(3H)-ylideneacetyl-CoA
3.3.2.12
3-Oxo-5,6-dehydrosuberyl-CoA semialdehyde
1.2.1.91
3-Oxo-5,6-didehydrosuberyl-CoA
2.3.1.223
Acetyl-CoA
2.3.1.192
2.3.1.14
Phenylacetylglycine
Phenylacetylglutamine
3-Oxoadipyl-CoA
1.1.1.157
(3S)-3-Hydroxyadipyl-CoA
PaaF
2,3-Didehydroadipyl-CoA
2.3.1.174
Succinyl-CoA
Citrate cycle
Acetyl-CoA
1-Phenylpropane-1,2-dione
2.6.1.-
Cathinone
Norpseudoephedrine
2.1.1.-
Pseudoephedrine
Norephedrine
2.1.1.-
Ephedrine
1.5.1.18
(R)-2-Methylimino-1-phenylpropan-1-ol
Salicylate
1.14.13.-
Benzoate
3.5.1.32
Hippurate
2.3.1.71
Benzoyl-CoA
trans-Cinnamate
1.14.13.11
1.14.13.14
trans-2-Hydroxycinnamate
trans-4-Hydroxycinnamate
1.3.1.31
1.3.1.11
6.2.1.12
Phenylpropanoate
2-Hydroxyphenylpropanoate
3-Hydroxyphenylpropanoate
4-Coumaroyl-CoA
4-Hydroxybenzoate
trans-3-Hydroxycinnamate
1.14.12.19
1.14.12.19
1.14.13.-
CI-dihydrodiol
PP-dihydrodiol
1.14.13.127
1.14.13.127
1.14.13.4
Caffeoyl-CoA
Benzoate degradation
1.3.1.87
1.3.1.87
2.1.1.104
trans-2,3-Dihydroxycinnamate
2,3-Dihydroxyphenylpropanoate
Feruloyl-CoA
1.13.11.16
1.13.11.16
4.2.1.101
Valine, leucine, isoleucine degradation
2-Hydroxy-6-oxonona-trienedioate
2-Hydroxy-6-oxonona-2,4-diene-1,9-dioate
4-Hydroxy-3-methoxyphenyl-beta-hydroxypropionyl-CoA
3.7.1.14
3.7.1.14
Succinate
Citrate cycle
4.1.2.41
Isobutyryl-CoA
cis-2-Hydroxypenta-2,4-dienoate
Fumarate
Vanillin
Malonyl-CoA
4.2.1.80
4-Hydroxy-2-oxopentanoate
4.1.3.39
Vanillylamine
8-Methyl-6-nonenoic acid
Pyruvate
Glycolysis
Acetaldehyde
Capsaicin
1.2.1.10
Acetyl-CoA
00360 6/4/18
(c) Kanehisa Laboratories

E

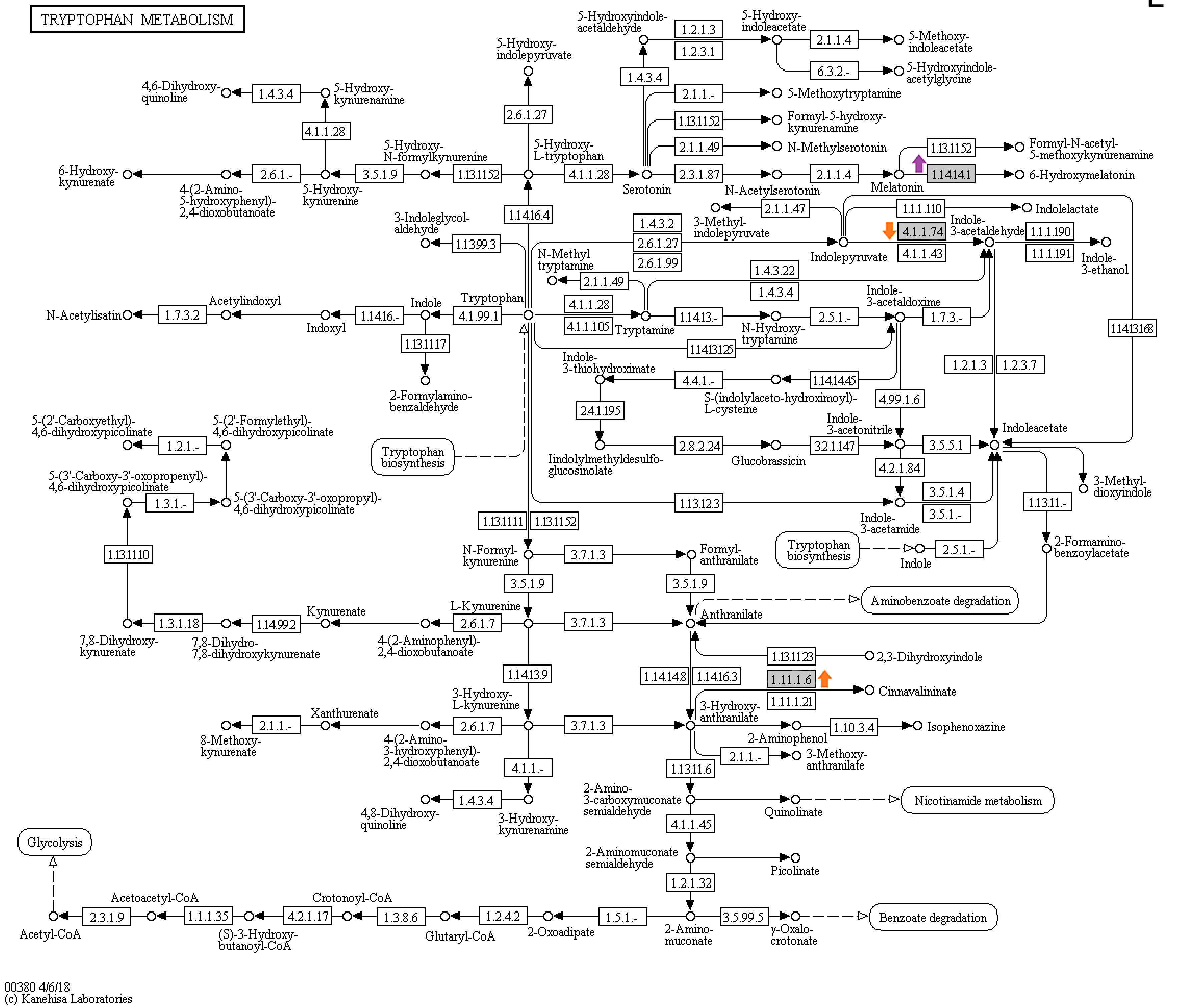

TRYPTOPHAN METABOLISM
5-Hydroxyindole-acetaldehyde
1.2.1.3
1.2.3.1
5-Hydroxy-indoleacetate
2.1.1.4
5-Methoxy-indoleacetate
6.3.2.-
5-Hydroxyindole-acetylglycine
5-Hydroxy-indolepyruvate
1.4.3.4
2.1.1.-
5-Methoxytryptamine
1.13.11.52
Formyl-5-hydroxy-kynurenamine
2.1.1.49
N-Methylserotonin
2.3.1.87
N-Acetylserotonin
Melatonin
Formyl-N-acetyl-5-methoxykynurenamine
1.14.14.1
6-Hydroxymelatonin
4,6-Dihydroxy-quinoline
5-Hydroxy-kynurenamine
4.1.1.28
2.6.1.27
6-Hydroxy-kynurenate
2.6.1.-
4-(2-Amino-5-hydroxyphenyl)-2,4-dioxobutanoate
5-Hydroxy-kynurenine
3.5.1.9
5-Hydroxy-N-formylkynurenine
5-Hydroxy-L-tryptophan
Serotonin
1.14.16.4
3-Indoleglycol-aldehyde
1.13.99.3
1.4.3.2
2.6.1.27
2.6.1.99
3-Methyl-indolepyruvate
2.1.1.47
Indolepyruvate
1.1.1.110
Indolelactate
4.1.1.74
4.1.1.43
Indole-3-acetaldehyde
1.1.1.190
1.1.1.191
Indole-3-ethanol
N-Methyl tryptamine
1.4.3.22
1.4.3.4
N-Acetylisatin
1.7.3.2
Acetylindoxyl
Indoxyl
1.14.16.-
Indole
Tryptophan
4.1.99.1
4.1.1.105
Tryptamine
1.14.13.-
N-Hydroxy-tryptamine
2.5.1.-
Indole-3-acetaldoxime
1.7.3.-
1.14.13.168
1.13.11.17
1.14.13.125
2-Formylamino-benzaldehyde
Indole-3-thiohydroximate
4.4.1.-
1.14.14.45
S-(indolylaceto-hydroximoyl)-L-cysteine
1.2.1.3
1.2.3.7
2.4.1.195
4.99.1.6
5-(2'-Carboxyethyl)-4,6-dihydroxypicolinate
1.2.1.-
5-(2'-Formylethyl)-4,6-dihydroxypicolinate
Tryptophan biosynthesis
Indolylmethyldesulfo-glucosinolate
2.8.2.24
Glucobrassicin
3.2.1.147
Indole-3-acetonitrile
3.5.5.1
Indoleacetate
4.2.1.84
3-Methyl-dioxyindole
5-(3'-Carboxy-3'-oxopropenyl)-4,6-dihydroxypicolinate
1.3.1.-
5-(3'-Carboxy-3'-oxopropyl)-4,6-dihydroxypicolinate
1.13.12.3
3.5.1.4
3.5.1.-
Indole-3-acetamide
1.13.11.-
1.13.11.11
1.13.11.52
1.13.11.10
N-Formyl-kynurenine
3.7.1.3
Formyl-anthranilate
2-Formamino-benzoylacetate
3.5.1.9
Aminobenzoate degradation
Anthranilate
7,8-Dihydroxy-kynurenate
1.3.1.18
7,8-Dihydro-7,8-dihydroxykynurenate
1.14.99.2
Kynurenate
4-(2-Aminophenyl)-2,4-dioxobutanoate
2.6.1.7
L-Kynurenine
1.13.11.23
2,3-Dihydroxyindole
1.14.13.9
1.14.14.8
1.14.16.3
1.11.1.6
Cinnavalininate
1.11.1.21
8-Methoxy-kynurenate
2.1.1.-
Xanthurenate
4-(2-Amino-3-hydroxyphenyl)-2,4-dioxobutanoate
3-Hydroxy-L-kynurenine
3-Hydroxy-anthranilate
2-Aminophenol
1.10.3.4
Isophenoxazine
3-Methoxy-anthranilate
4.1.1.-
1.13.11.6
4,8-Dihydroxy-quinoline
3-Hydroxy-kynurenamine
2-Amino-3-carboxymuconate semialdehyde
Quinolinate
Nicotinamide metabolism
4.1.1.45
2-Aminomuconate semialdehyde
Picolinate
1.2.1.32
Glycolysis
Acetyl-CoA
2.3.1.9
Acetoacetyl-CoA
1.1.1.35
(S)-3-Hydroxy-butanoyl-CoA
4.2.1.17
Crotonoyl-CoA
1.3.8.6
Glutaryl-CoA
1.2.4.2
2-Oxoadipate
1.5.1.-
2-Amino-muconate
3.5.99.5
γ-Oxalo-crotonate
Benzoate degradation
00380 4/6/18
(c) Kanehisa Laboratories

F

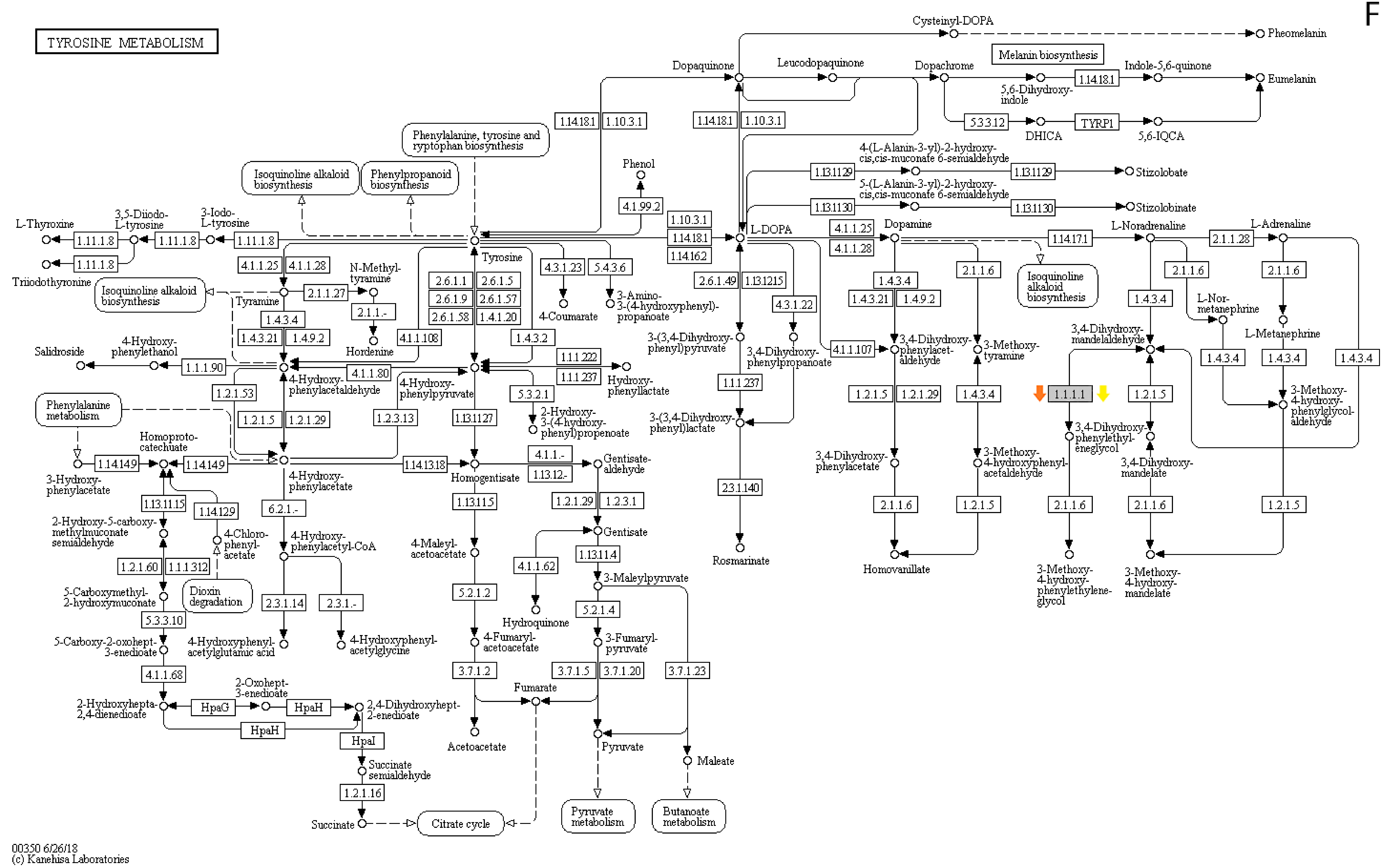
TYROSINE METABOLISM
Cysteinyl-DOPA
Pheomelanin
Melanin biosynthesis
Dopaquinone
Leucodopaquinone
Dopachrome
Indole-5,6-quinone
Eumelanin
5,6-Dihydroxy-indole
1.14.18.1
5.3.3.12
DHICA
TYRP1
5,6-IQCA
1.14.18.1
1.10.3.1
Phenylalanine, tyrosine and ryptophan biosynthesis
Isoquinoline alkaloid biosynthesis
Phenylpropanoid biosynthesis
Phenol
4.1.99.2
4-(L-Alanin-3-yl)-2-hydroxy-cis,cis-muconate 6-semialdehyde
1.13.11.29
Stizolobate
5-(L-Alanin-3-yl)-2-hydroxy-cis,cis-muconate 6-semialdehyde
1.13.11.30
Stizolobinate
L-Thyroxine
3,5-Diiodo-L-tyrosine
3-Iodo-L-tyrosine
1.11.1.8
Triiodothyronine
Tyrosine
1.10.3.1
1.14.18.1
1.14.16.2
L-DOPA
4.1.1.25
4.1.1.28
Dopamine
1.14.17.1
L-Noradrenaline
2.1.1.28
L-Adrenaline
N-Methyl-tyramine
Tyramine
2.1.1.27
2.1.1.-
Hordenine
2.6.1.1
2.6.1.5
2.6.1.9
2.6.1.57
2.6.1.58
1.4.1.20
4.3.1.23
5.4.3.6
4-Coumarate
3-Amino-3-(4-hydroxyphenyl)-propanoate
2.6.1.49
1.13.12.15
4.3.1.22
1.4.3.4
1.4.3.21
1.4.9.2
2.1.1.6
Isoquinoline alkaloid biosynthesis
L-Nor-metanephrine
L-Metanephrine
3,4-Dihydroxy-mandelaldehyde
4-Hydroxy-phenylethanol
Salidroside
1.1.1.90
4.1.1.108
1.4.3.2
1.1.1.222
1.1.1.237
Hydroxy-phenyllactate
3-(3,4-Dihydroxy-phenyl)pyruvate
3,4-Dihydroxy-phenylpropanoate
4.1.1.107
3,4-Dihydroxy-phenylacet-aldehyde
3-Methoxy-tyramine
4-Hydroxy-phenylacetaldehyde
4.1.1.80
4-Hydroxy-phenylpyruvate
1.2.1.53
5.3.2.1
2-Hydroxy-3-(4-hydroxy-phenyl)propenoate
1.2.1.5
1.2.1.29
1.2.3.13
1.13.11.27
1.1.1.1
3-Methoxy-4-hydroxy-phenylglycol-aldehyde
Phenylalanine metabolism
3,4-Dihydroxy-phenylethyl-eneglycol
3,4-Dihydroxy-mandelate
3-(3,4-Dihydroxy-phenyl)lactate
3,4-Dihydroxy-phenylacetate
3-Methoxy-4-hydroxyphenyl-acetaldehyde
Homoproto-catechuate
1.14.14.9
3-Hydroxy-phenylacetate
4-Hydroxy-phenylacetate
1.14.13.18
Homogentisate
4.1.1.-
1.13.12.-
Gentisate-aldehyde
2.3.1.140
1.13.11.15
1.14.12.9
6.2.1.-
1.13.11.5
1.2.1.29
1.2.3.1
2-Hydroxy-5-carboxy-methylmuconate semialdehyde
4-Chloro-phenyl-acetate
4-Hydroxy-phenylacetyl-CoA
4-Maleyl-acetoacetate
Gentisate
Rosmarinate
Homovanillate
3-Methoxy-4-hydroxy-phenylethylene-glycol
3-Methoxy-4-hydroxy-mandelate
1.2.1.60
1.1.1.312
Dioxin degradation
4.1.1.62
1.13.11.4
5-Carboxymethyl-2-hydroxymuconate
2.3.1.14
2.3.1.-
5.2.1.2
3-Maleylpyruvate
Hydroquinone
5.3.3.10
5.2.1.4
5-Carboxy-2-oxohept-3-enedioate
4-Hydroxyphenyl-acetylglutamic acid
4-Hydroxyphenyl-acetylglycine
4-Fumaryl-acetoacetate
3-Fumaryl-pyruvate
4.1.1.68
3.7.1.2
3.7.1.5
3.7.1.20
3.7.1.23
2-Oxohept-3-enedioate
Fumarate
2-Hydroxyhepta-2,4-dienedioate
HpaG
HpaH
2,4-Dihydroxyhept-2-enedioate
HpaH
HpaI
Acetoacetate
Pyruvate
Maleate
Succinate semialdehyde
1.2.1.16
Succinate
Citrate cycle
Pyruvate metabolism
Butanoate metabolism
00350 6/26/18
(c) Kanehisa Laboratories

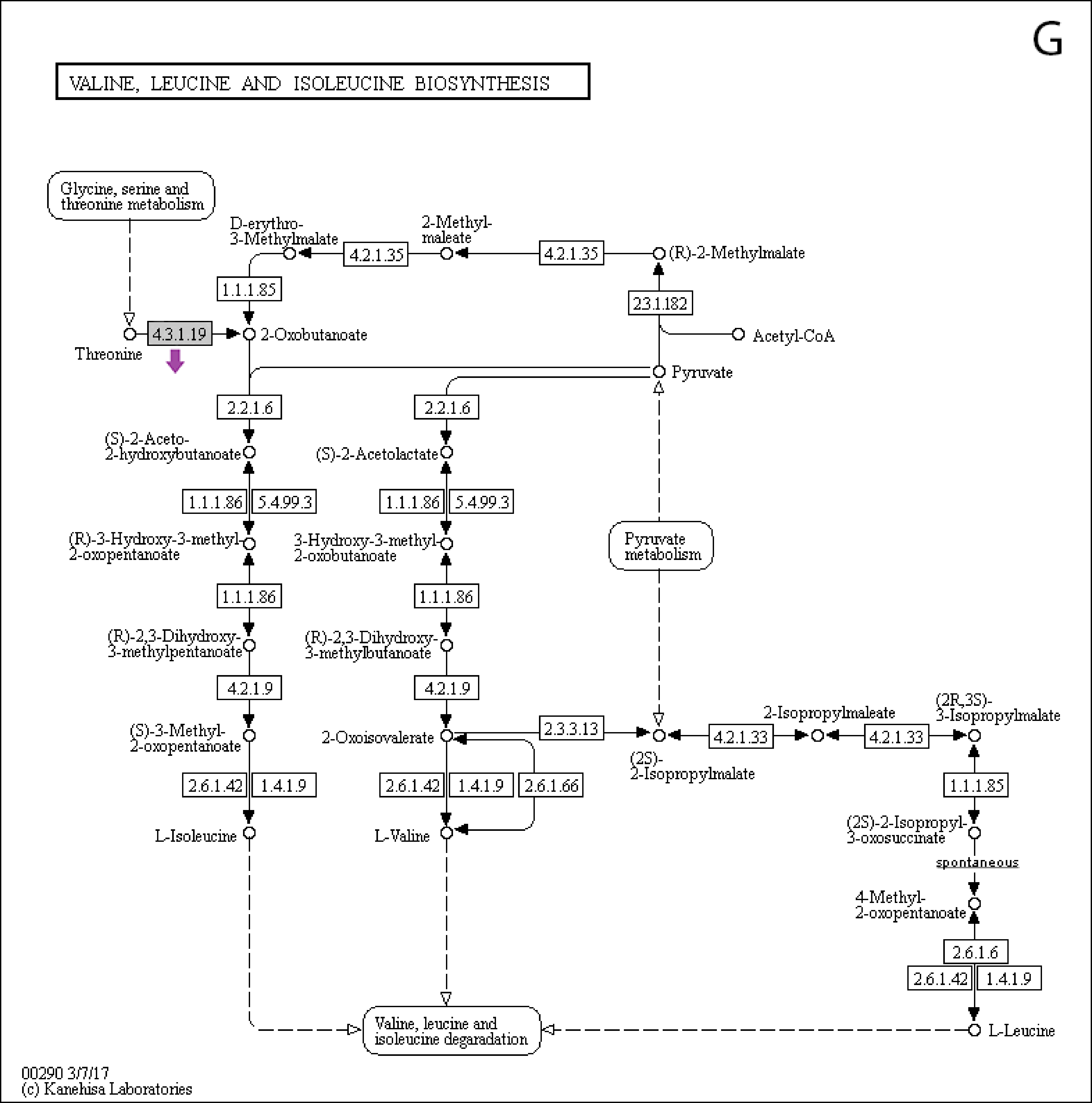
G
VALINE, LEUCINE AND ISOLEUCINE BIOSYNTHESIS
Glycine, serine and threonine metabolism
D-erythro-3-Methylmalate
4.2.1.35
2-Methyl-maleate
4.2.1.35
(R)-2-Methylmalate
1.1.1.85
2.3.1.182
Threonine
4.3.1.19
2-Oxobutanoate
Acetyl-CoA
Pyruvate
2.2.1.6
2.2.1.6
(S)-2-Aceto-2-hydroxybutanoate
(S)-2-Acetolactate
1.1.1.86
5.4.99.3
1.1.1.86
5.4.99.3
(R)-3-Hydroxy-3-methyl-2-oxopentanoate
3-Hydroxy-3-methyl-2-oxobutanoate
Pyruvate metabolism
1.1.1.86
1.1.1.86
(R)-2,3-Dihydroxy-3-methylpentanoate
(R)-2,3-Dihydroxy-3-methylbutanoate
4.2.1.9
4.2.1.9
(S)-3-Methyl-2-oxopentanoate
2-Oxoisovalerate
2.3.3.13
(2S)-2-Isopropylmalate
4.2.1.33
2-Isopropylmaleate
4.2.1.33
(2R,3S)-3-Isopropylmalate
2.6.1.42
1.4.1.9
2.6.1.42
1.4.1.9
2.6.1.66
1.1.1.85
L-Isoleucine
L-Valine
(2S)-2-Isopropyl-3-oxosuccinate
spontaneous
4-Methyl-2-oxopentanoate
2.6.1.6
2.6.1.42
1.4.1.9
Valine, leucine and isoleucine degaradation
L-Leucine
00290 3/7/17
(c) Kanehisa Laboratories

H

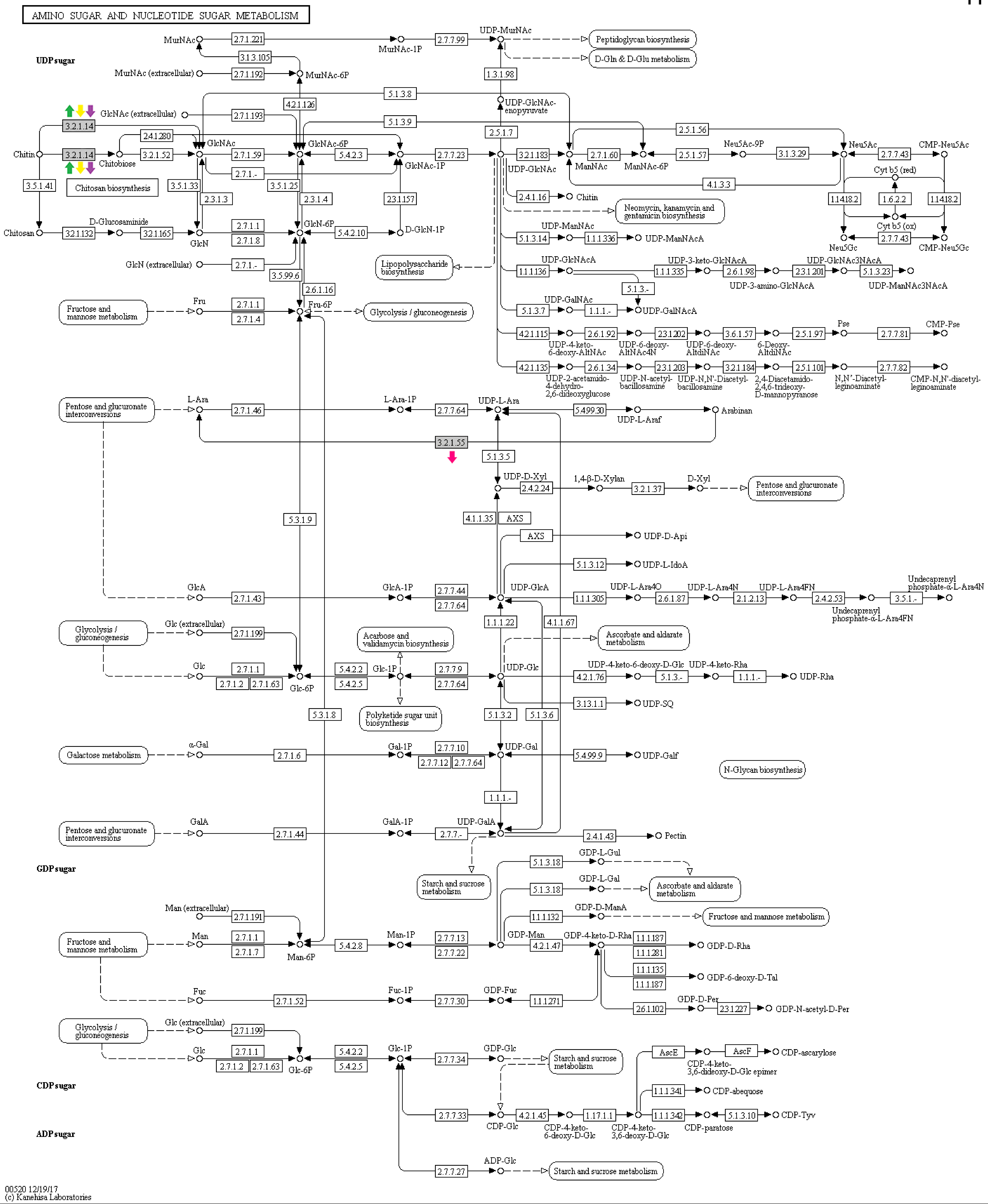
AMINO SUGAR AND NUCLEOTIDE SUGAR METABOLISM
UDP sugar
GDP sugar
CDP sugar
ADP sugar
MurNAc
2.7.1.221
MurNAc-1P
2.7.7.99
UDP-MurNAc
Peptidoglycan biosynthesis
D-Gln & D-Glu metabolism
3.1.3.105
MurNAc (extracellular)
2.7.1.192
MurNAc-6P
1.3.1.98
4.2.1.126
5.1.3.8
UDP-GlcNAc-enopyruvate
GlcNAc (extracellular)
2.7.1.193
3.2.1.14
2.4.1.280
GlcNAc
GlcNAc-6P
5.1.3.9
2.5.1.7
2.5.1.56
Neu5Ac-9P
Neu5Ac
CMP-Neu5Ac
Chitin
Chitobiose
3.2.1.52
2.7.1.59
5.4.2.3
2.7.7.23
GlcNAc-1P
3.2.1.183
UDP-GlcNAc
2.7.1.60
ManNAc
ManNAc-6P
2.5.1.57
3.1.3.29
2.7.7.43
2.7.1.-
4.1.3.3
Cyt b5 (red)
Cyt b5 (ox)
1.14.18.2
1.6.2.2
3.5.1.41
Chitosan biosynthesis
3.5.1.33
2.3.1.3
3.5.1.25
2.3.1.4
2.3.1.157
2.4.1.16
Chitin
Neomycin, kanamycin and gentamicin biosynthesis
Neu5Gc
CMP-Neu5Gc
Chitosan
D-Glucosaminide
3.2.1.132
3.2.1.165
GlcN
2.7.1.1
2.7.1.8
GlcN-6P
5.4.2.10
D-GlcN-1P
UDP-ManNAc
5.1.3.14
1.1.1.336
UDP-ManNAcA
GlcN (extracellular)
Lipopolysaccharide biosynthesis
UDP-GlcNAcA
1.1.1.136
UDP-3-keto-GlcNAcA
1.1.1.335
2.6.1.98
UDP-GlcNAc3NAcA
2.3.1.201
5.1.3.23
UDP-3-amino-GlcNAcA
UDP-ManNAc3NAcA
3.5.99.6
5.1.3.-
2.6.1.16
UDP-GalNAc
5.1.3.7
1.1.1.-
UDP-GalNAcA
Fructose and mannose metabolism
Fru
2.7.1.4
Fru-6P
Glycolysis / gluconeogenesis
4.2.1.115
2.6.1.92
2.3.1.202
3.6.1.57
2.5.1.97
Pse
2.7.7.81
CMP-Pse
UDP-4-keto-6-deoxy-AltNAc
UDP-6-deoxy-AltNAc4N
UDP-6-deoxy-AltdiNAc
6-Deoxy-AltdiNAc
4.2.1.135
2.6.1.34
2.3.1.203
3.2.1.184
2.5.1.101
2.7.7.82
UDP-2-acetamido-4-dehydro-2,6-dideoxyglucose
UDP-N-acetyl-bacillosamine
UDP-N,N'-Diacetyl-bacillosamine
2,4-Diacetamido-2,4,6-trideoxy-D-mannopyranose
N,N'-Diacetyl-legionaminate
CMP-N,N'-diacetyl-legionaminate
Pentose and glucuronate interconversions
L-Ara
2.7.1.46
L-Ara-1P
2.7.7.64
UDP-L-Ara
5.4.99.30
UDP-L-Araf
Arabinan
3.2.1.55
5.1.3.5
UDP-D-Xyl
2.4.2.24
1,4-β-D-Xylan
3.2.1.37
D-Xyl
5.3.1.9
4.1.1.35
AXS
UDP-D-Api
5.1.3.12
UDP-L-IdoA
GlcA
2.7.1.43
GlcA-1P
2.7.7.44
UDP-GlcA
1.1.1.305
UDP-L-Ara4O
2.6.1.87
UDP-L-Ara4N
2.1.2.13
UDP-L-Ara4FN
2.4.2.53
Undecaprenyl phosphate-α-L-Ara4FN
3.5.1.-
Undecaprenyl phosphate-α-L-Ara4N
1.1.1.22
4.1.1.67
Glc (extracellular)
2.7.1.199
Acarbose and validamycin biosynthesis
Ascorbate and aldarate metabolism
Glc
2.7.1.2
2.7.1.63
Glc-6P
5.4.2.2
5.4.2.5
Glc-1P
2.7.7.9
UDP-Glc
UDP-4-keto-6-deoxy-D-Glc
4.2.1.76
UDP-4-keto-Rha
UDP-Rha
3.13.1.1
UDP-SQ
5.3.1.8
Polyketide sugar unit biosynthesis
5.1.3.2
5.1.3.6
Galactose metabolism
α-Gal
2.7.1.6
Gal-1P
2.7.7.10
2.7.7.12
UDP-Gal
5.4.99.9
UDP-Galf
N-Glycan biosynthesis
GalA
2.7.1.44
GalA-1P
2.7.7.-
UDP-GalA
2.4.1.43
Pectin
GDP-L-Gul
5.1.3.18
Starch and sucrose metabolism
GDP-L-Gal
GDP-D-ManA
1.1.1.132
Man (extracellular)
2.7.1.191
Man
2.7.1.1
2.7.1.7
Man-6P
5.4.2.8
Man-1P
2.7.7.13
2.7.7.22
GDP-Man
4.2.1.47
GDP-4-keto-D-Rha
1.1.1.187
1.1.1.281
GDP-D-Rha
1.1.1.135
GDP-6-deoxy-D-Tal
Fuc
2.7.1.52
Fuc-1P
2.7.7.30
GDP-Fuc
1.1.1.271
GDP-D-Per
2.6.1.102
2.3.1.227
GDP-N-acetyl-D-Per
GDP-Glc
2.7.7.34
AscE
AscF
CDP-ascarylose
CDP-4-keto-3,6-dideoxy-D-Glc epimer
1.1.1.341
CDP-abequose
2.7.7.33
CDP-Glc
4.2.1.45
CDP-4-keto-6-deoxy-D-Glc
1.17.1.1
CDP-4-keto-3,6-dideoxy-D-Glc
1.1.1.342
CDP-paratose
5.1.3.10
CDP-Tyv
ADP-Glc
2.7.7.27
00520 12/19/17
(c) Kanehisa Laboratories

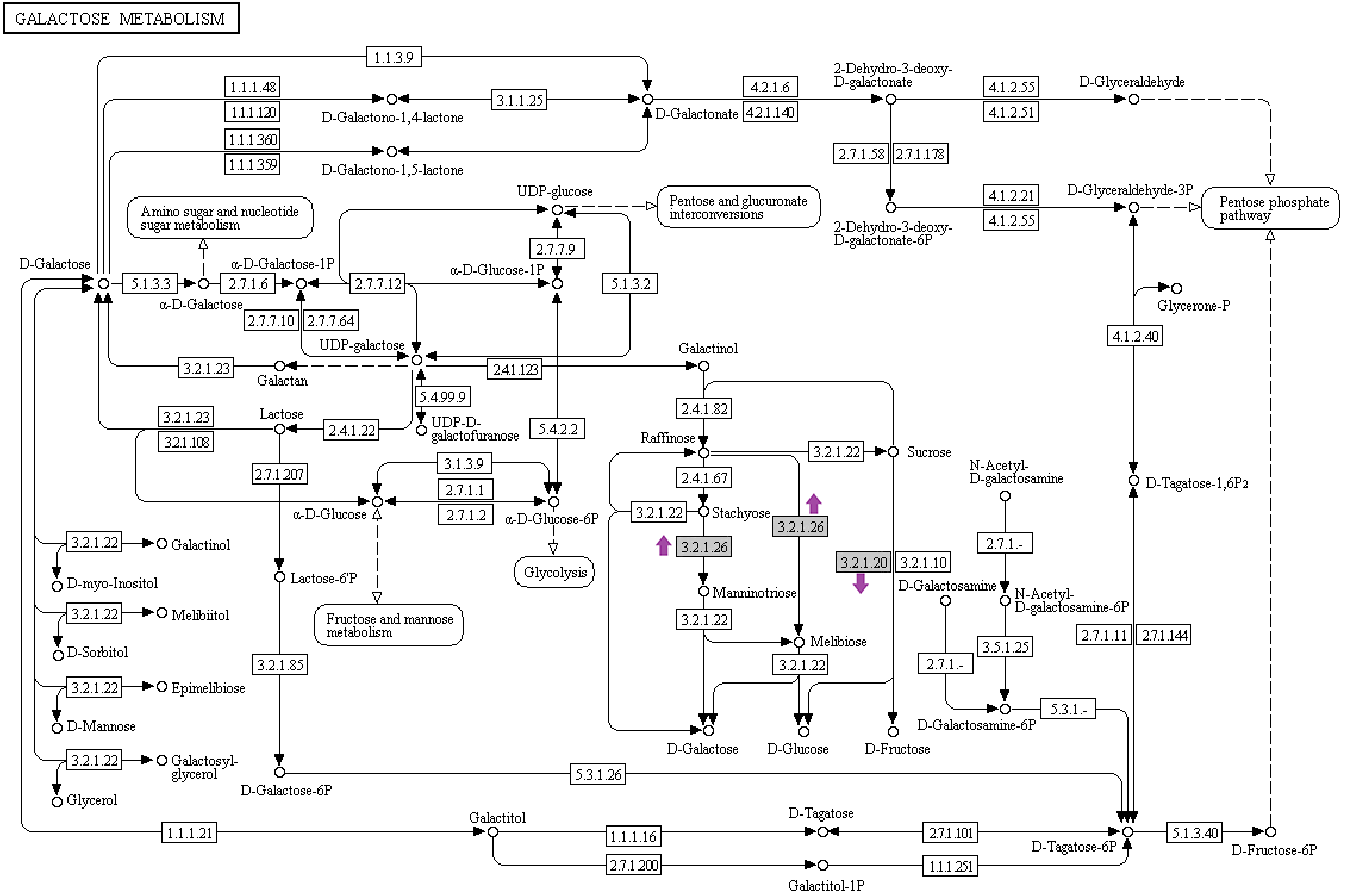

00052 10/17/17
(c) Kanehisa Laboratories

J

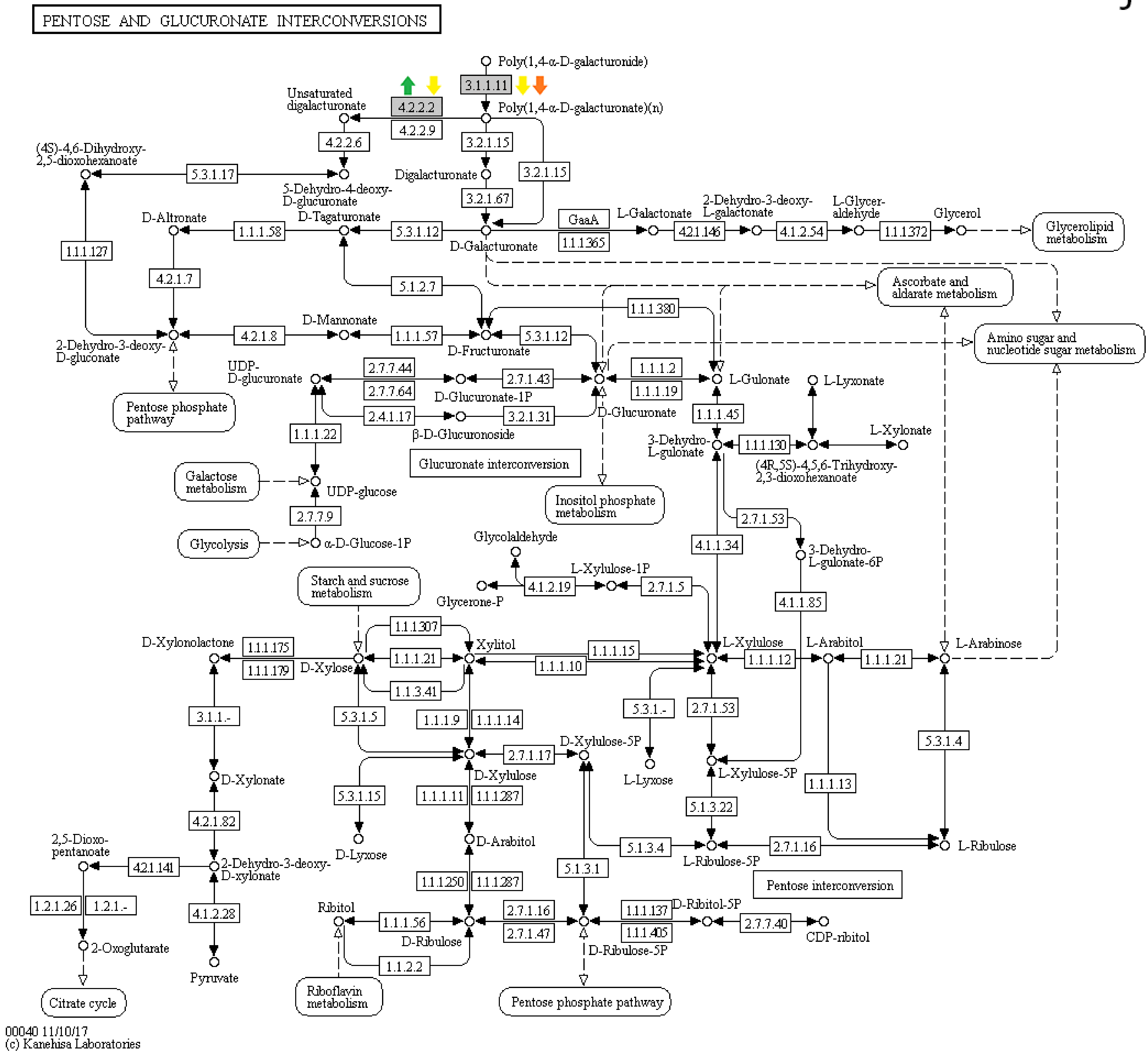

PENTOSE AND GLUCURONATE INTERCONVERSIONS
Poly(1,4-α-D-galacturonide)
3.1.1.11
Poly(1,4-α-D-galacturonate)(n)
Unsaturated digalacturonate
4.2.2.2
4.2.2.9
4.2.2.6
3.2.1.15
3.2.1.15
(4S)-4,6-Dihydroxy-2,5-dioxohexanoate
5.3.1.17
5-Dehydro-4-deoxy-D-glucuronate
Digalacturonate
3.2.1.67
D-Altronate
1.1.1.58
D-Tagaturonate
5.3.1.12
D-Galacturonate
GaaA
1.1.1.365
L-Galactonate
4.2.1.146
2-Dehydro-3-deoxy-L-galactonate
4.1.2.54
L-Glyceraldehyde
1.1.1.372
Glycerol
Glycerolipid metabolism
1.1.1.127
4.2.1.7
5.1.2.7
Ascorbate and aldarate metabolism
1.1.1.380
D-Mannonate
4.2.1.8
1.1.1.57
5.3.1.12
D-Fructuronate
Amino sugar and nucleotide sugar metabolism
2-Dehydro-3-deoxy-D-gluconate
UDP-D-glucuronate
2.7.7.44
2.7.7.64
2.7.1.43
D-Glucuronate-1P
1.1.1.2
1.1.1.19
L-Gulonate
L-Lyxonate
Pentose phosphate pathway
2.4.1.17
3.2.1.31
β-D-Glucuronoside
D-Glucuronate
1.1.1.45
1.1.1.22
3-Dehydro-L-gulonate
1.1.1.130
L-Xylonate
(4R,5S)-4,5,6-Trihydroxy-2,3-dioxohexanoate
Glucuronate interconversion
Galactose metabolism
UDP-glucose
Inositol phosphate metabolism
2.7.7.9
2.7.1.53
Glycolysis
α-D-Glucose-1P
Glycolaldehyde
4.1.1.34
3-Dehydro-L-gulonate-6P
L-Xylulose-1P
4.1.2.19
2.7.1.5
Starch and sucrose metabolism
Glycerone-P
4.1.1.85
1.1.1.307
D-Xylonolactone
1.1.1.175
1.1.1.179
Xylitol
1.1.1.15
L-Xylulose
L-Arabitol
D-Xylose
1.1.1.21
1.1.1.10
1.1.1.12
1.1.1.21
L-Arabinose
1.1.3.41
3.1.1.-
5.3.1.5
1.1.1.9
1.1.1.14
5.3.1.-
2.7.1.53
5.3.1.4
2.7.1.17
D-Xylulose-5P
L-Lyxose
L-Xylulose-5P
D-Xylonate
D-Xylulose
5.3.1.15
1.1.1.11
1.1.1.287
1.1.1.13
4.2.1.82
5.1.3.22
D-Lyxose
D-Arabitol
2,5-Dioxopentanoate
4.2.1.141
2-Dehydro-3-deoxy-D-xylonate
5.1.3.4
2.7.1.16
L-Ribulose
L-Ribulose-5P
5.1.3.1
Pentose interconversion
1.1.1.250
1.1.1.287
1.2.1.26
1.2.1.-
4.1.2.28
Ribitol
1.1.1.56
2.7.1.16
2.7.1.47
1.1.1.137
1.1.1.405
D-Ribitol-5P
2.7.7.40
CDP-ribitol
D-Ribulose
D-Ribulose-5P
2-Oxoglutarate
Pyruvate
1.1.2.2
Citrate cycle
Riboflavin metabolism
Pentose phosphate pathway
00040 11/10/17
(c) Kanehisa Laboratories

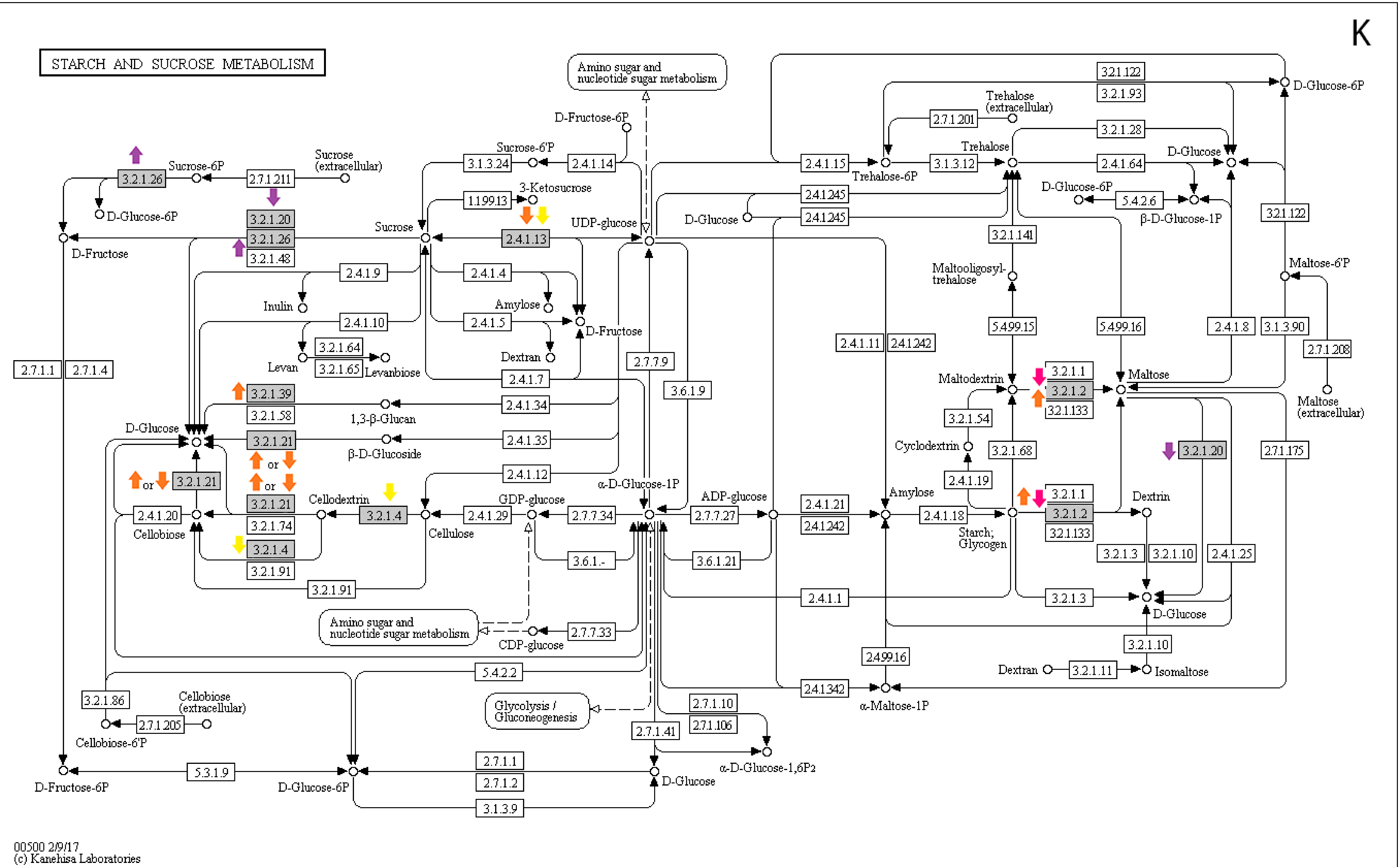
K
STARCH AND SUCROSE METABOLISM
Amino sugar and nucleotide sugar metabolism
Sucrose-6P
3.2.1.26
D-Glucose-6P
2.7.1.211
Sucrose (extracellular)
3.2.1.20
3.2.1.26
3.2.1.48
D-Fructose
Sucrose
2.4.1.13
UDP-glucose
3.1.3.24
Sucrose-6'P
2.4.1.14
D-Fructose-6P
1.1.99.13
3-Ketosucrose
2.4.1.9
Inulin
2.4.1.4
Amylose
2.4.1.10
Levan
3.2.1.64
3.2.1.65
Levanbiose
2.4.1.5
Dextran
D-Fructose
2.4.1.7
2.7.7.9
3.6.1.9
2.7.1.1
2.7.1.4
3.2.1.39
3.2.1.58
1,3-β-Glucan
2.4.1.34
D-Glucose
3.2.1.21
β-D-Glucoside
2.4.1.35
or
3.2.1.21
2.4.1.12
Cellodextrin
3.2.1.4
Cellulose
GDP-glucose
2.4.1.29
2.7.7.34
α-D-Glucose-1P
ADP-glucose
2.7.7.27
2.4.1.20
Cellobiose
3.2.1.74
3.2.1.91
3.6.1.-
3.6.1.21
Amino sugar and nucleotide sugar metabolism
CDP-glucose
2.7.7.33
5.4.2.2
3.2.1.86
Cellobiose (extracellular)
2.7.1.205
Cellobiose-6'P
Glycolysis / Gluconeogenesis
2.7.1.41
2.7.1.10
2.7.1.106
α-D-Glucose-1,6P2
D-Fructose-6P
5.3.1.9
D-Glucose-6P
2.7.1.1
2.7.1.2
3.1.3.9
D-Glucose
3.2.1.122
3.2.1.93
D-Glucose-6P
Trehalose (extracellular)
2.7.1.201
3.2.1.28
Trehalose
D-Glucose
2.4.1.15
Trehalose-6P
3.1.3.12
2.4.1.64
2.4.1.245
D-Glucose
D-Glucose-6P
5.4.2.6
β-D-Glucose-1P
3.2.1.122
3.2.1.141
Maltooligosyl-trehalose
Maltose-6'P
5.4.99.15
5.4.99.16
2.4.1.8
3.1.3.90
2.7.1.208
2.4.1.11
2.4.1.242
Maltodextrin
3.2.1.1
3.2.1.2
3.2.1.133
Maltose
Maltose (extracellular)
3.2.1.54
Cyclodextrin
3.2.1.68
3.2.1.20
2.7.1.175
2.4.1.19
Amylose
2.4.1.21
2.4.1.242
2.4.1.18
Starch; Glycogen
Dextrin
3.2.1.3
3.2.1.10
2.4.1.25
2.4.1.1
D-Glucose
3.2.1.10
2.4.99.16
Dextran
3.2.1.11
Isomaltose
2.4.1.342
α-Maltose-1P
00500 2/9/17
(c) Kanehisa Laboratories

L

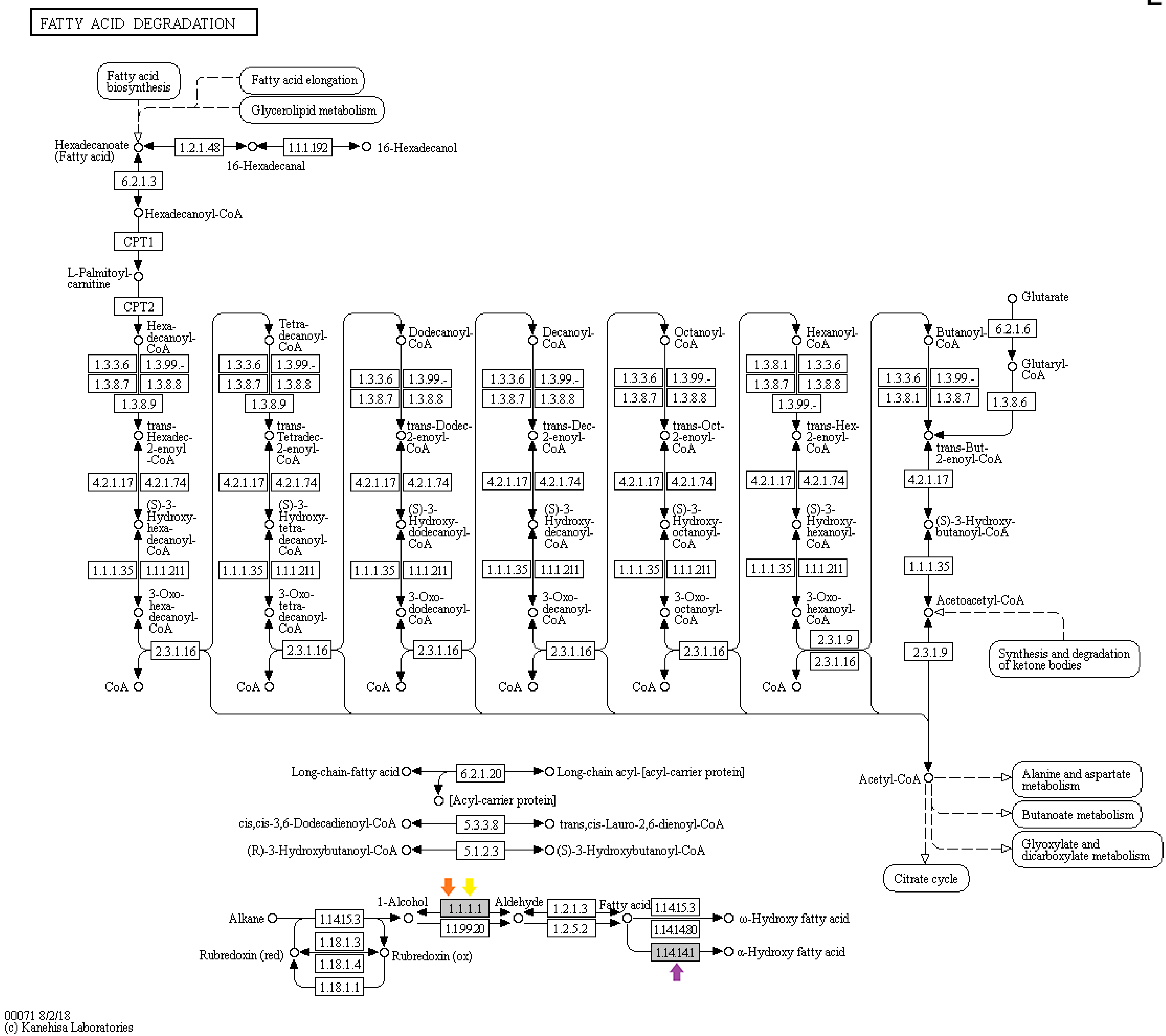
FATTY ACID DEGRADATION
Fatty acid biosynthesis
Fatty acid elongation
Glycerolipid metabolism
Hexadecanoate (Fatty acid)
1.2.1.48
1.1.1.192
16-Hexadecanol
16-Hexadecanal
6.2.1.3
Hexadecanoyl-CoA
CPT1
L-Palmitoyl-carnitine
CPT2
Hexa-decanoyl-CoA
Tetra-decanoyl-CoA
Dodecanoyl-CoA
Decanoyl-CoA
Octanoyl-CoA
Hexanoyl-CoA
Butanoyl-CoA
Glutarate
6.2.1.6
Glutaryl-CoA
1.3.8.6
1.3.3.6
1.3.99.-
1.3.8.7
1.3.8.8
1.3.8.9
1.3.8.1
trans-Hexadec-2-enoyl-CoA
trans-Tetradec-2-enoyl-CoA
trans-Dodec-2-enoyl-CoA
trans-Dec-2-enoyl-CoA
trans-Oct-2-enoyl-CoA
trans-Hex-2-enoyl-CoA
trans-But-2-enoyl-CoA
4.2.1.17
4.2.1.74
(S)-3-Hydroxy-hexa-decanoyl-CoA
(S)-3-Hydroxy-tetra-decanoyl-CoA
(S)-3-Hydroxy-dodecanoyl-CoA
(S)-3-Hydroxy-decanoyl-CoA
(S)-3-Hydroxy-octanoyl-CoA
(S)-3-Hydroxy-hexanoyl-CoA
(S)-3-Hydroxy-butanoyl-CoA
1.1.1.35
1.1.1.211
3-Oxo-hexa-decanoyl-CoA
3-Oxo-tetra-decanoyl-CoA
3-Oxo-dodecanoyl-CoA
3-Oxo-decanoyl-CoA
3-Oxo-octanoyl-CoA
3-Oxo-hexanoyl-CoA
Acetoacetyl-CoA
2.3.1.16
2.3.1.9
CoA
Synthesis and degradation of ketone bodies
Acetyl-CoA
Alanine and aspartate metabolism
Butanoate metabolism
Glyoxylate and dicarboxylate metabolism
Citrate cycle
Long-chain-fatty acid
6.2.1.20
Long-chain acyl-[acyl-carrier protein]
[Acyl-carrier protein]
cis,cis-3,6-Dodecadienoyl-CoA
5.3.3.8
trans,cis-Lauro-2,6-dienoyl-CoA
(R)-3-Hydroxybutanoyl-CoA
5.1.2.3
(S)-3-Hydroxybutanoyl-CoA
Alkane
1.14.15.3
1-Alcohol
1.1.1.1
1.1.99.20
Aldehyde
1.2.1.3
1.2.5.2
Fatty acid
1.14.14.80
1.14.14.1
ω-Hydroxy fatty acid
α-Hydroxy fatty acid
1.18.1.3
1.18.1.4
1.18.1.1
Rubredoxin (red)
Rubredoxin (ox)
00071 8/2/18
(c) Kanehisa Laboratories

M

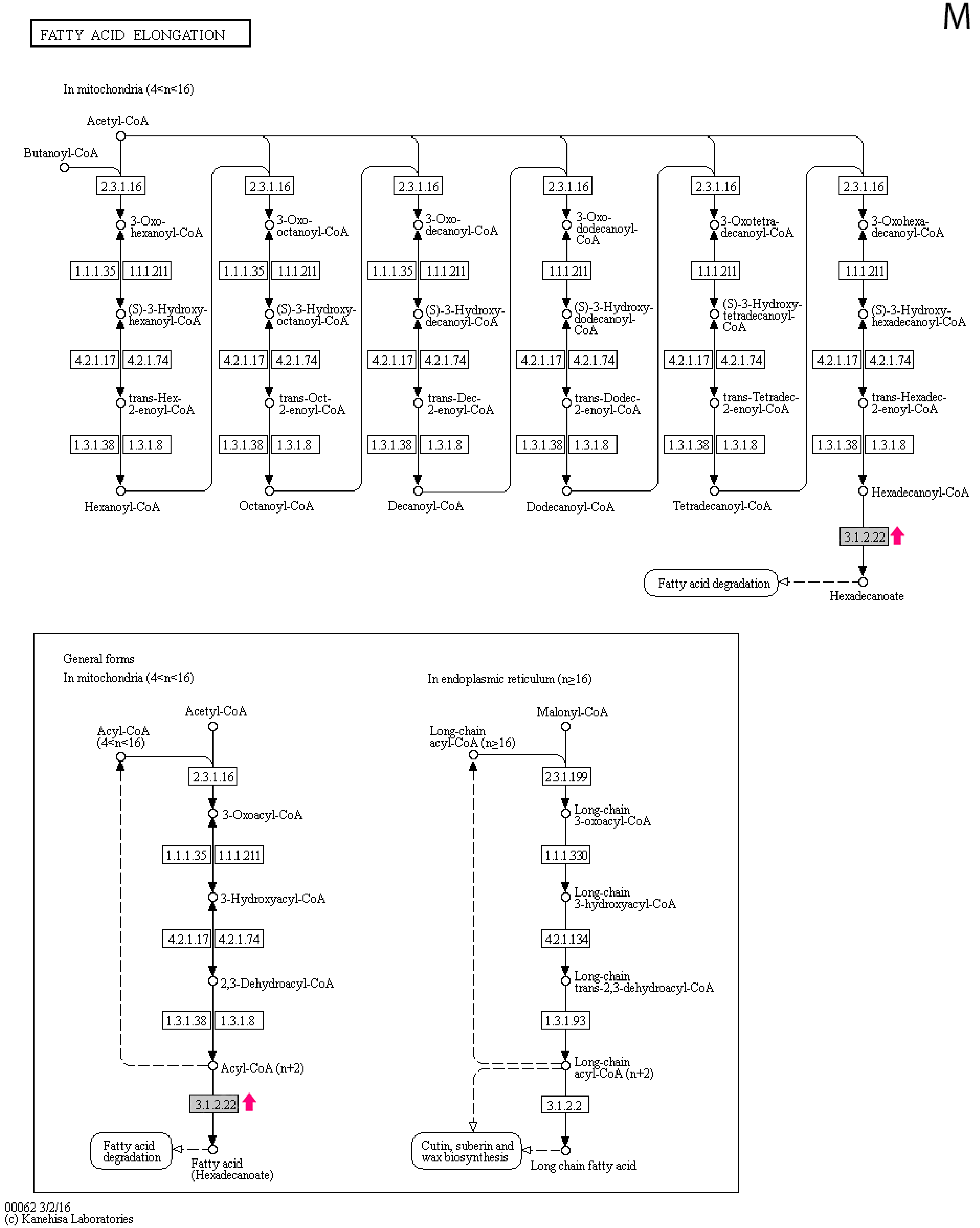
FATTY ACID ELONGATION
In mitochondria (4<n<16)
Acetyl-CoA
Butanoyl-CoA
2.3.1.16
3-Oxo-hexanoyl-CoA
1.1.1.35
1.1.1.211
(S)-3-Hydroxy-hexanoyl-CoA
4.2.1.17
4.2.1.74
trans-Hex-2-enoyl-CoA
1.3.1.38
1.3.1.8
Hexanoyl-CoA
3-Oxo-octanoyl-CoA
(S)-3-Hydroxy-octanoyl-CoA
trans-Oct-2-enoyl-CoA
Octanoyl-CoA
3-Oxo-decanoyl-CoA
(S)-3-Hydroxy-decanoyl-CoA
trans-Dec-2-enoyl-CoA
Decanoyl-CoA
3-Oxo-dodecanoyl-CoA
(S)-3-Hydroxy-dodecanoyl-CoA
trans-Dodec-2-enoyl-CoA
Dodecanoyl-CoA
3-Oxotetra-decanoyl-CoA
(S)-3-Hydroxy-tetradecanoyl-CoA
trans-Tetradec-2-enoyl-CoA
Tetradecanoyl-CoA
3-Oxohexa-decanoyl-CoA
(S)-3-Hydroxy-hexadecanoyl-CoA
trans-Hexadec-2-enoyl-CoA
Hexadecanoyl-CoA
3.1.2.22
Fatty acid degradation
Hexadecanoate
General forms
In mitochondria (4<n<16)
Acetyl-CoA
Acyl-CoA (4<n<16)
2.3.1.16
3-Oxoacyl-CoA
1.1.1.35
1.1.1.211
3-Hydroxyacyl-CoA
4.2.1.17
4.2.1.74
2,3-Dehydroacyl-CoA
1.3.1.38
1.3.1.8
Acyl-CoA (n+2)
3.1.2.22
Fatty acid degradation
Fatty acid (Hexadecanoate)
In endoplasmic reticulum (n≥16)
Malonyl-CoA
Long-chain acyl-CoA (n≥16)
2.3.1.199
Long-chain 3-oxoacyl-CoA
1.1.1.330
Long-chain 3-hydroxyacyl-CoA
4.2.1.134
Long-chain trans-2,3-dehydroacyl-CoA
1.3.1.93
Long-chain acyl-CoA (n+2)
3.1.2.2
Cutin, suberin and wax biosynthesis
Long chain fatty acid
00062 3/2/16
(c) Kanehisa Laboratories

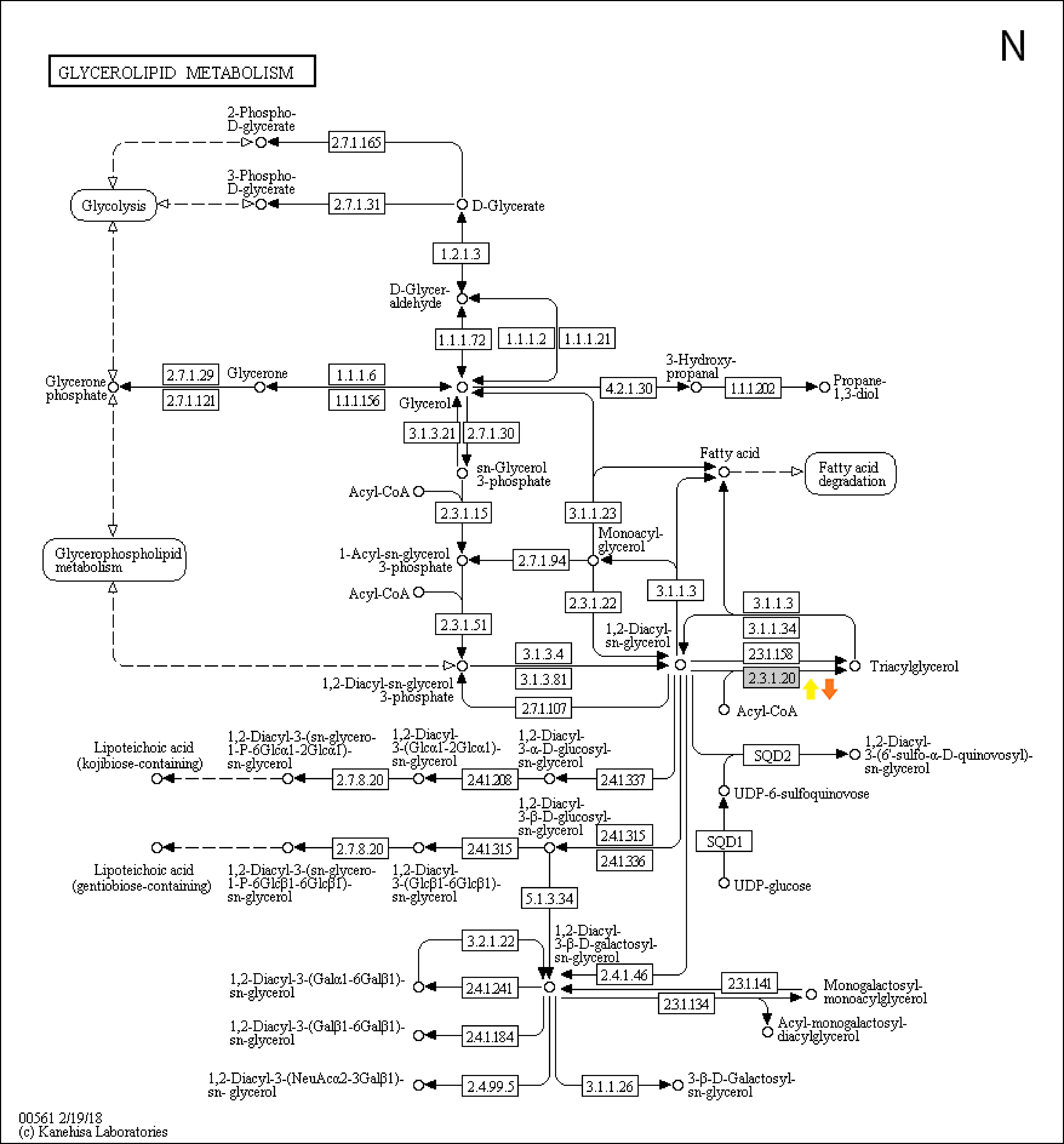
N
GLYCEROLIPID METABOLISM
2-Phospho-D-glycerate
2.7.1.165
3-Phospho-D-glycerate
2.7.1.31
Glycolysis
D-Glycerate
1.2.1.3
D-Glyceraldehyde
1.1.1.72
1.1.1.2
1.1.1.21
Glycerone phosphate
2.7.1.29
2.7.1.121
Glycerone
1.1.1.6
1.1.1.156
Glycerol
4.2.1.30
3-Hydroxy-propanal
1.1.1.202
Propane-1,3-diol
3.1.3.21
2.7.1.30
sn-Glycerol 3-phosphate
Fatty acid
Fatty acid degradation
Acyl-CoA
2.3.1.15
3.1.1.23
Monoacylglycerol
Glycerophospholipid metabolism
1-Acyl-sn-glycerol 3-phosphate
2.7.1.94
Acyl-CoA
3.1.1.3
2.3.1.22
3.1.1.3
2.3.1.51
1,2-Diacyl-sn-glycerol
3.1.1.34
3.1.3.4
2.3.1.158
Triacylglycerol
3.1.3.81
2.3.1.20
1,2-Diacyl-sn-glycerol 3-phosphate
2.7.1.107
Acyl-CoA
Lipoteichoic acid (kojibiose-containing)
1,2-Diacyl-3-(sn-glycero-1-P-6Glcα1-2Glcα1)-sn-glycerol
1,2-Diacyl-3-(Glcα1-2Glcα1)-sn-glycerol
1,2-Diacyl-3-α-D-glucosyl-sn-glycerol
SQD2
1,2-Diacyl-3-(6'-sulfo-α-D-quinovosyl)-sn-glycerol
2.7.8.20
2.4.1.208
2.4.1.337
UDP-6-sulfoquinovose
1,2-Diacyl-3-β-D-glucosyl-sn-glycerol
2.4.1.315
SQD1
2.7.8.20
2.4.1.315
2.4.1.336
UDP-glucose
Lipoteichoic acid (gentiobiose-containing)
1,2-Diacyl-3-(sn-glycero-1-P-6Glcβ1-6Glcβ1)-sn-glycerol
1,2-Diacyl-3-(Glcβ1-6Glcβ1)-sn-glycerol
5.1.3.34
1,2-Diacyl-3-β-D-galactosyl-sn-glycerol
3.2.1.22
2.4.1.46
1,2-Diacyl-3-(Galα1-6Galβ1)-sn-glycerol
2.4.1.241
2.3.1.141
Monogalactosyl-monoacylglycerol
2.3.1.134
Acyl-monogalactosyl-diacylglycerol
1,2-Diacyl-3-(Galβ1-6Galβ1)-sn-glycerol
2.4.1.184
1,2-Diacyl-3-(NeuAcα2-3Galβ1)-sn- glycerol
2.4.99.5
3.1.1.26
3-β-D-Galactosyl-sn-glycerol
00561 2/19/18
(c) Kanehisa Laboratories

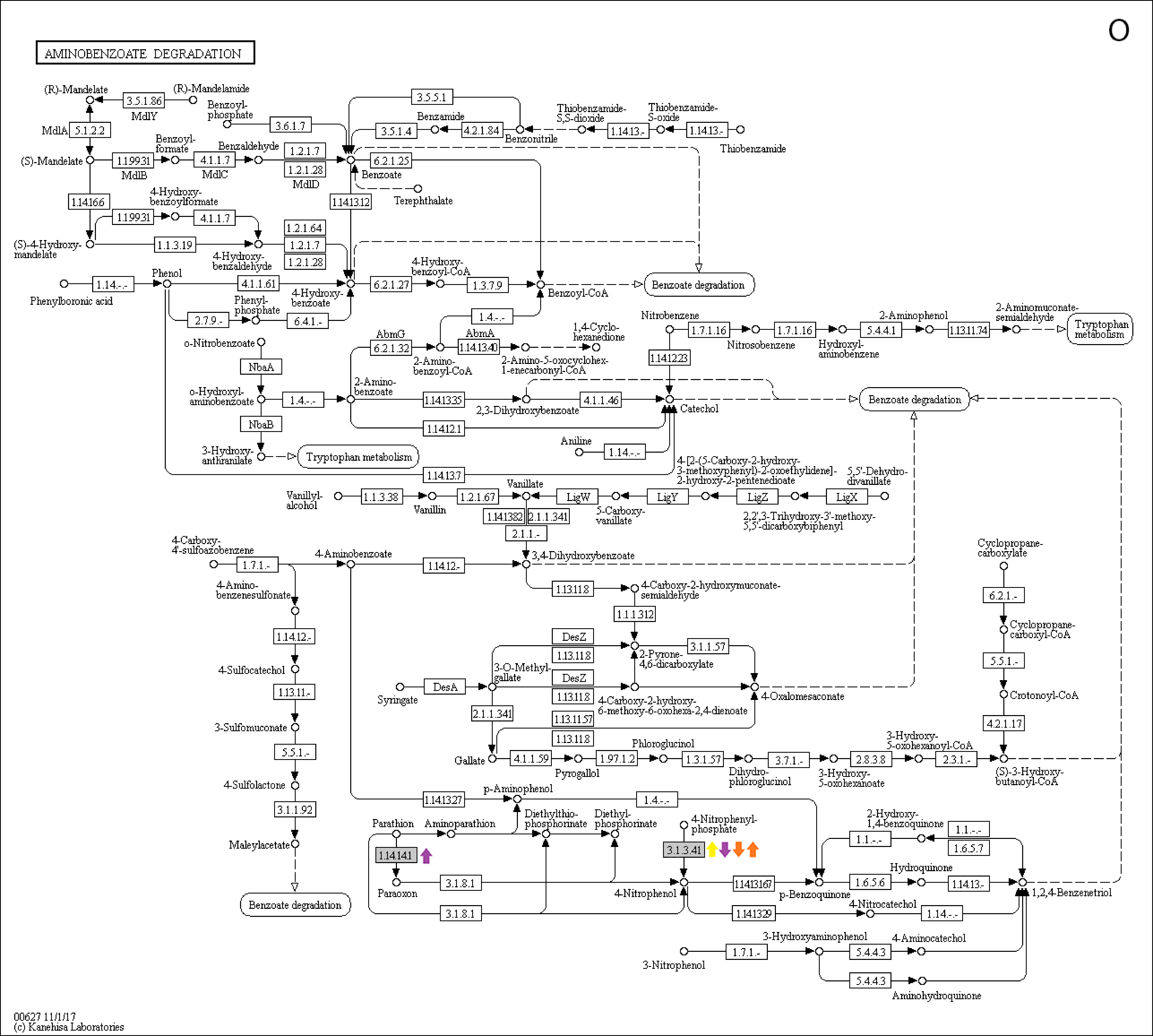
O
AMINOBENZOATE DEGRADATION
(R)-Mandelate
3.5.1.86
MdlY
(R)-Mandelamide
Benzoyl-phosphate
3.6.1.7
3.5.5.1
Benzamide
3.5.1.4
4.2.1.84
Benzonitrile
Thiobenzamide-S,S-dioxide
1.14.13.-
Thiobenzamide-S-oxide
1.14.13.-
Thiobenzamide
MdlA
5.1.2.2
(S)-Mandelate
1.1.99.31
MdlB
Benzoyl-formate
4.1.1.7
MdlC
Benzaldehyde
1.2.1.7
1.2.1.28
MdlD
6.2.1.25
Benzoate
Terephthalate
1.14.13.12
1.14.16.6
4-Hydroxy-benzoylformate
1.1.99.31
4.1.1.7
(S)-4-Hydroxy-mandelate
1.1.3.19
4-Hydroxy-benzaldehyde
1.2.1.64
1.2.1.7
1.2.1.28
Phenylboronic acid
1.14.-.-
Phenol
4.1.1.61
4-Hydroxy-benzoate
6.2.1.27
4-Hydroxy-benzoyl-CoA
1.3.7.9
Benzoyl-CoA
Benzoate degradation
2.7.9.-
Phenyl-phosphate
6.4.1.-
1.4.-.-
AbmG
6.2.1.32
2-Amino-benzoyl-CoA
AbmA
1.14.13.40
1,4-Cyclo-hexanedione
2-Amino-5-oxocyclohex-1-enecarbonyl-CoA
o-Nitrobenzoate
NbaA
o-Hydroxyl-aminobenzoate
1.4.-.-
2-Amino-benzoate
1.14.13.35
2,3-Dihydroxybenzoate
4.1.1.46
Catechol
1.14.12.1
NbaB
3-Hydroxy-anthranilate
Tryptophan metabolism
Aniline
1.14.-.-
1.14.13.7
Nitrobenzene
1.14.12.23
1.7.1.16
Nitrosobenzene
1.7.1.16
Hydroxyl-aminobenzene
2-Aminophenol
5.4.4.1
1.13.11.74
2-Aminomuconate-semialdehyde
Tryptophan metabolism
Benzoate degradation
4-[2-(5-Carboxy-2-hydroxy-3-methoxyphenyl)-2-oxoethylidene]-2-hydroxy-2-pentenedioate
5,5'-Dehydro-divanillate
Vanillyl-alcohol
1.1.3.38
Vanillin
1.2.1.67
Vanillate
LigW
5-Carboxy-vanillate
LigY
LigZ
LigX
2,2',3-Trihydroxy-3'-methoxy-5,5'-dicarboxybiphenyl
1.14.13.82
2.1.1.341
2.1.-.-
4-Carboxy-4'-sulfoazobenzene
1.7.1.-
4-Aminobenzoate
1.14.12.-
3,4-Dihydroxybenzoate
4-Amino-benzenesulfonate
1.13.11.8
4-Carboxy-2-hydroxymuconate-semialdehyde
1.1.1.312
1.14.12.-
4-Sulfocatechol
1.13.11.-
3-Sulfomuconate
5.5.1.-
4-Sulfolactone
3.1.1.92
Maleylacetate
Benzoate degradation
DesZ
1.13.11.8
2-Pyrone-4,6-dicarboxylate
3.1.1.57
3-O-Methyl-gallate
DesZ
1.13.11.8
Syringate
DesA
4-Carboxy-2-hydroxy-6-methoxy-6-oxohexa-2,4-dienoate
4-Oxalomesaconate
2.1.1.341
1.13.11.57
1.13.11.8
Cyclopropane-carboxylate
6.2.1.-
Cyclopropane-carboxyl-CoA
5.5.1.-
Crotonoyl-CoA
4.2.1.17
Gallate
4.1.1.59
Pyrogallol
Phloroglucinol
1.97.1.2
1.3.1.57
Dihydro-phloroglucinol
3.7.1.-
3-Hydroxy-5-oxohexanoate
2.8.3.8
3-Hydroxy-5-oxohexanoyl-CoA
2.3.1.-
(S)-3-Hydroxy-butanoyl-CoA
1.14.13.27
p-Aminophenol
1.4.-.-
Parathion
Aminoparathion
Diethylthio-phosphorinate
Diethyl-phosphorinate
4-Nitrophenyl-phosphate
2-Hydroxy-1,4-benzoquinone
1.1.-.-
1.1.-.-
1.6.5.7
1.14.14.1
3.1.3.41
Paraoxon
3.1.8.1
4-Nitrophenol
1.14.13.167
p-Benzoquinone
1.6.5.6
Hydroquinone
1.14.13.-
1,2,4-Benzenetriol
3.1.8.1
1.14.13.29
4-Nitrocatechol
1.14.-.-
3-Hydroxyaminophenol
4-Aminocatechol
3-Nitrophenol
1.7.1.-
5.4.4.3
5.4.4.3
Aminohydroquinone
00627 11/1/17
(c) Kanehisa Laboratories

P

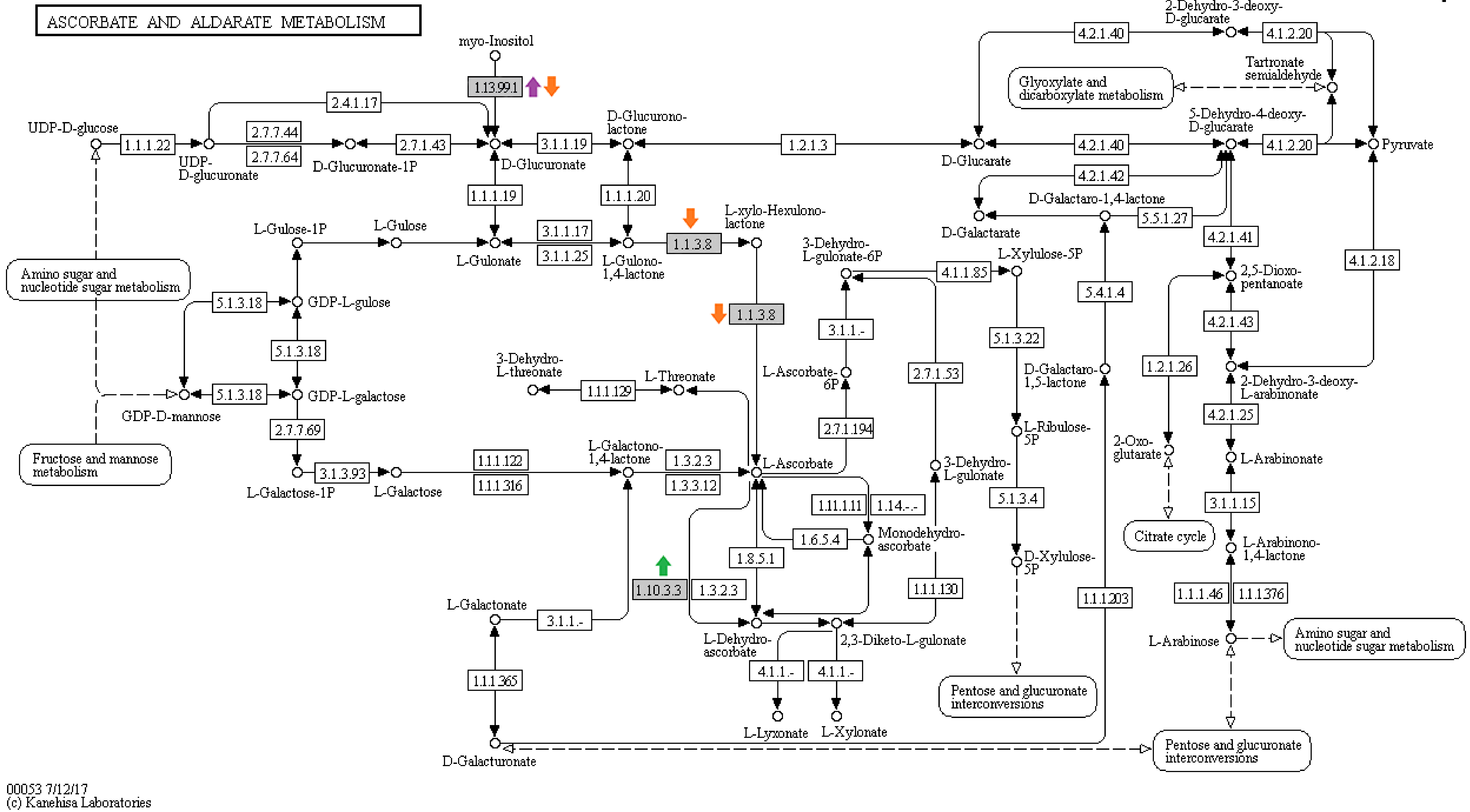
ASCORBATE AND ALDARATE METABOLISM
myo-Inositol
1.13.99.1
2.4.1.17
UDP-D-glucose
1.1.1.22
UDP-
D-glucuronate
2.7.7.44
2.7.7.64
D-Glucuronate-1P
2.7.1.43
D-Glucuronate
3.1.1.19
D-Glucurono-
lactone
1.2.1.3
D-Glucarate
4.2.1.40
2-Dehydro-3-deoxy-
D-glucarate
4.1.2.20
Tartronate
semialdehyde
Glyoxylate and
dicarboxylate metabolism
5-Dehydro-4-deoxy-
D-glucarate
4.2.1.40
4.1.2.20
Pyruvate
4.2.1.42
D-Galactaro-1,4-lactone
D-Galactarate
5.5.1.27
1.1.1.19
1.1.1.20
L-Gulose-1P
L-Gulose
3.1.1.17
3.1.1.25
L-Gulonate
L-Gulono-
1,4-lactone
1.1.3.8
L-xylo-Hexulono-
lactone
3-Dehydro-
L-gulonate-6P
4.1.1.85
L-Xylulose-5P
4.2.1.41
2,5-Dioxo-
pentanoate
4.1.2.18
5.4.1.4
Amino sugar and
nucleotide sugar metabolism
5.1.3.18
GDP-L-gulose
1.1.3.8
3.1.1.-
5.1.3.22
4.2.1.43
5.1.3.18
3-Dehydro-
L-threonate
L-Threonate
1.1.1.129
L-Ascorbate-
6P
2.7.1.53
D-Galactaro-
1,5-lactone
1.2.1.26
2-Dehydro-3-deoxy-
L-arabinonate
5.1.3.18
GDP-L-galactose
GDP-D-mannose
2.7.7.69
2.7.1.194
L-Ribulose-
5P
2-Oxo-
glutarate
4.2.1.25
L-Galactono-
1,4-lactone
1.1.1.122
1.1.1.316
1.3.2.3
1.3.3.12
L-Ascorbate
3-Dehydro-
L-gulonate
L-Arabinonate
Fructose and mannose
metabolism
3.1.3.93
L-Galactose-1P
L-Galactose
1.11.1.11
1.14.-.-
5.1.3.4
3.1.1.15
1.6.5.4
Monodehydro-
ascorbate
Citrate cycle
L-Arabinono-
1,4-lactone
1.8.5.1
D-Xylulose-
5P
1.10.3.3
1.3.2.3
1.1.1.130
1.1.1.203
1.1.1.46
1.1.1.376
L-Galactonate
3.1.1.-
L-Dehydro-
ascorbate
2,3-Diketo-L-gulonate
L-Arabinose
Amino sugar and
nucleotide sugar metabolism
1.1.1.365
4.1.1.-
4.1.1.-
Pentose and glucuronate
interconversions
L-Lyxonate
L-Xylonate
D-Galacturonate
Pentose and glucuronate
interconversions
00053 7/12/17
(c) Kanehisa Laboratories

Q

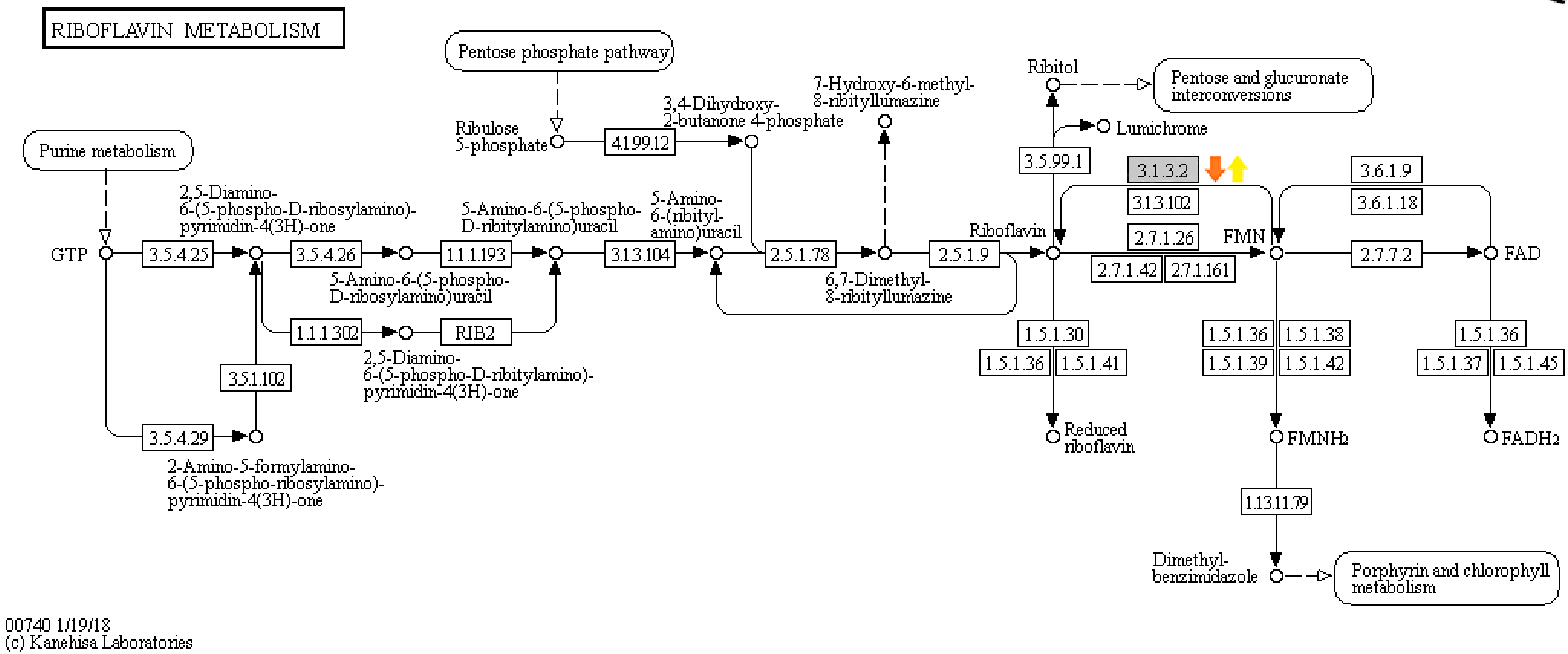
RIBOFLAVIN METABOLISM
Pentose phosphate pathway
Purine metabolism
Ribulose 5-phosphate
4.1.99.12
3,4-Dihydroxy-2-butanone 4-phosphate
7-Hydroxy-6-methyl-8-ribityllumazine
Ribitol
Pentose and glucuronate interconversions
Lumichrome
3.5.99.1
3.1.3.2
3.1.3.102
3.6.1.9
3.6.1.18
GTP
3.5.4.25
2,5-Diamino-6-(5-phospho-D-ribosylamino)-pyrimidin-4(3H)-one
3.5.4.26
5-Amino-6-(5-phospho-D-ribosylamino)uracil
1.1.1.193
5-Amino-6-(5-phospho-D-ribitylamino)uracil
3.1.3.104
5-Amino-6-(ribitylamino)uracil
2.5.1.78
6,7-Dimethyl-8-ribityllumazine
2.5.1.9
Riboflavin
2.7.1.26
2.7.1.42
2.7.1.161
FMN
2.7.7.2
FAD
1.1.1.302
RIB2
2,5-Diamino-6-(5-phospho-D-ribitylamino)-pyrimidin-4(3H)-one
3.5.1.102
3.5.4.29
2-Amino-5-formylamino-6-(5-phospho-ribosylamino)-pyrimidin-4(3H)-one
1.5.1.30
1.5.1.36
1.5.1.41
Reduced riboflavin
1.5.1.36
1.5.1.38
1.5.1.39
1.5.1.42
FMNH2
1.13.11.79
Dimethyl-benzimidazole
Porphyrin and chlorophyll metabolism
1.5.1.36
1.5.1.37
1.5.1.45
FADH2
00740 1/19/18
(c) Kanehisa Laboratories

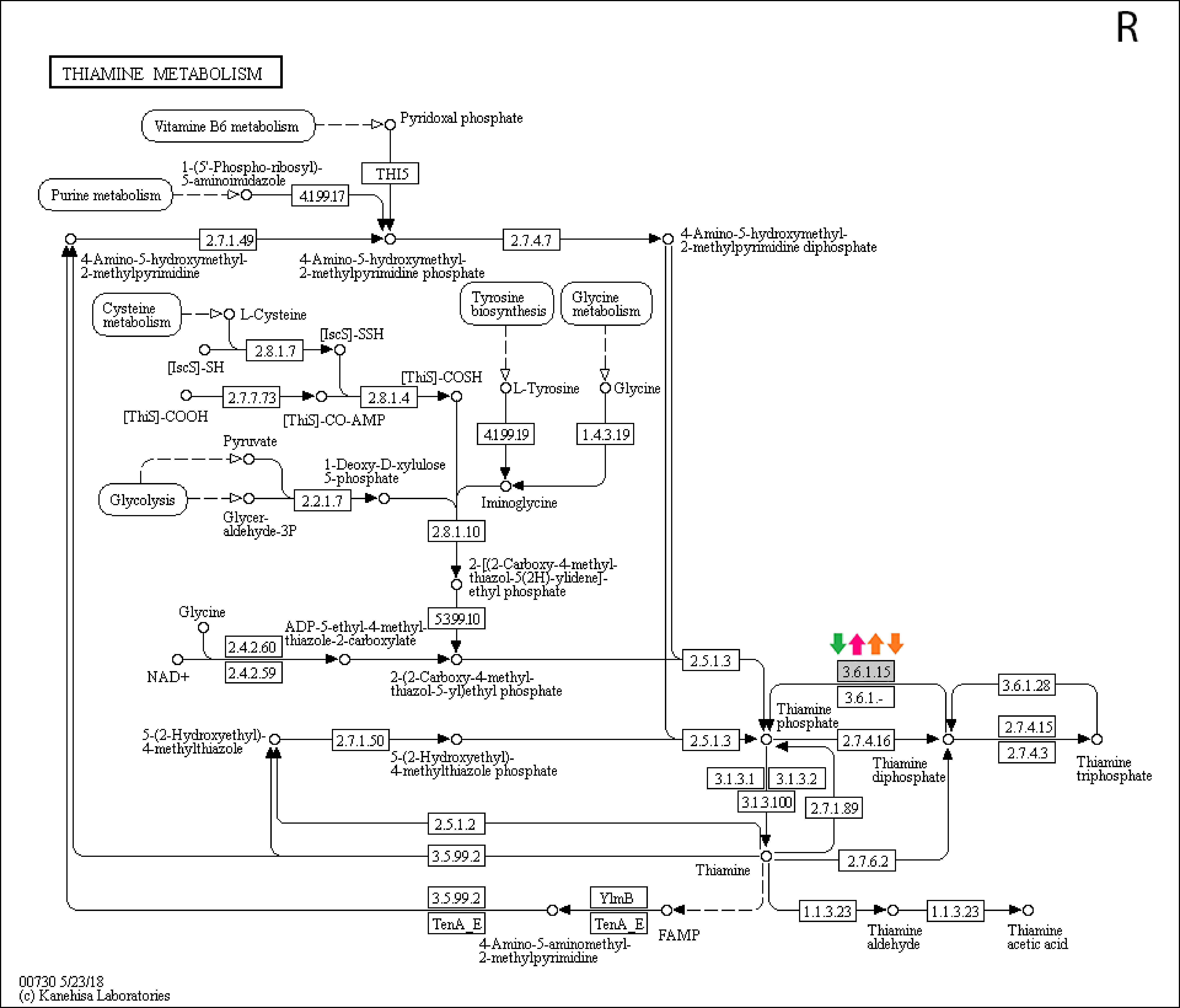
R
THIAMINE METABOLISM
Vitamine B6 metabolism
Pyridoxal phosphate
THI5
Purine metabolism
1-(5'-Phospho-ribosyl)-
5-aminoimidazole
4.1.99.17
2.7.1.49
2.7.4.7
4-Amino-5-hydroxymethyl-
2-methylpyrimidine
4-Amino-5-hydroxymethyl-
2-methylpyrimidine phosphate
4-Amino-5-hydroxymethyl-
2-methylpyrimidine diphosphate
Cysteine metabolism
L-Cysteine
2.8.1.7
[IscS]-SSH
[IscS]-SH
Tyrosine biosynthesis
Glycine metabolism
[ThiS]-COSH
2.7.7.73
2.8.1.4
[ThiS]-COOH
[ThiS]-CO-AMP
L-Tyrosine
Glycine
4.1.99.19
1.4.3.19
Pyruvate
1-Deoxy-D-xylulose
5-phosphate
Glycolysis
2.2.1.7
Iminoglycine
Glycer-
aldehyde-3P
2.8.1.10
2-[(2-Carboxy-4-methyl-
thiazol-5(2H)-ylidene]-
ethyl phosphate
5.3.99.10
Glycine
ADP-5-ethyl-4-methyl-
thiazole-2-carboxylate
2.4.2.60
2.4.2.59
NAD+
2-(2-Carboxy-4-methyl-
thiazol-5-yl)ethyl phosphate
2.5.1.3
3.6.1.15
3.6.1.-
3.6.1.28
Thiamine
phosphate
5-(2-Hydroxyethyl)-
4-methylthiazole
2.7.1.50
5-(2-Hydroxyethyl)-
4-methylthiazole phosphate
2.5.1.3
2.7.4.16
2.7.4.15
2.7.4.3
Thiamine
diphosphate
Thiamine
triphosphate
3.1.3.1
3.1.3.2
3.1.3.100
2.7.1.89
2.5.1.2
3.5.99.2
Thiamine
2.7.6.2
3.5.99.2
TenA_E
YlmB
TenA_E
FAMP
1.1.3.23
1.1.3.23
Thiamine
aldehyde
Thiamine
acetic acid
4-Amino-5-aminomethyl-
2-methylpyrimidine
00730 5/23/18
(c) Kanehisa Laboratories

S

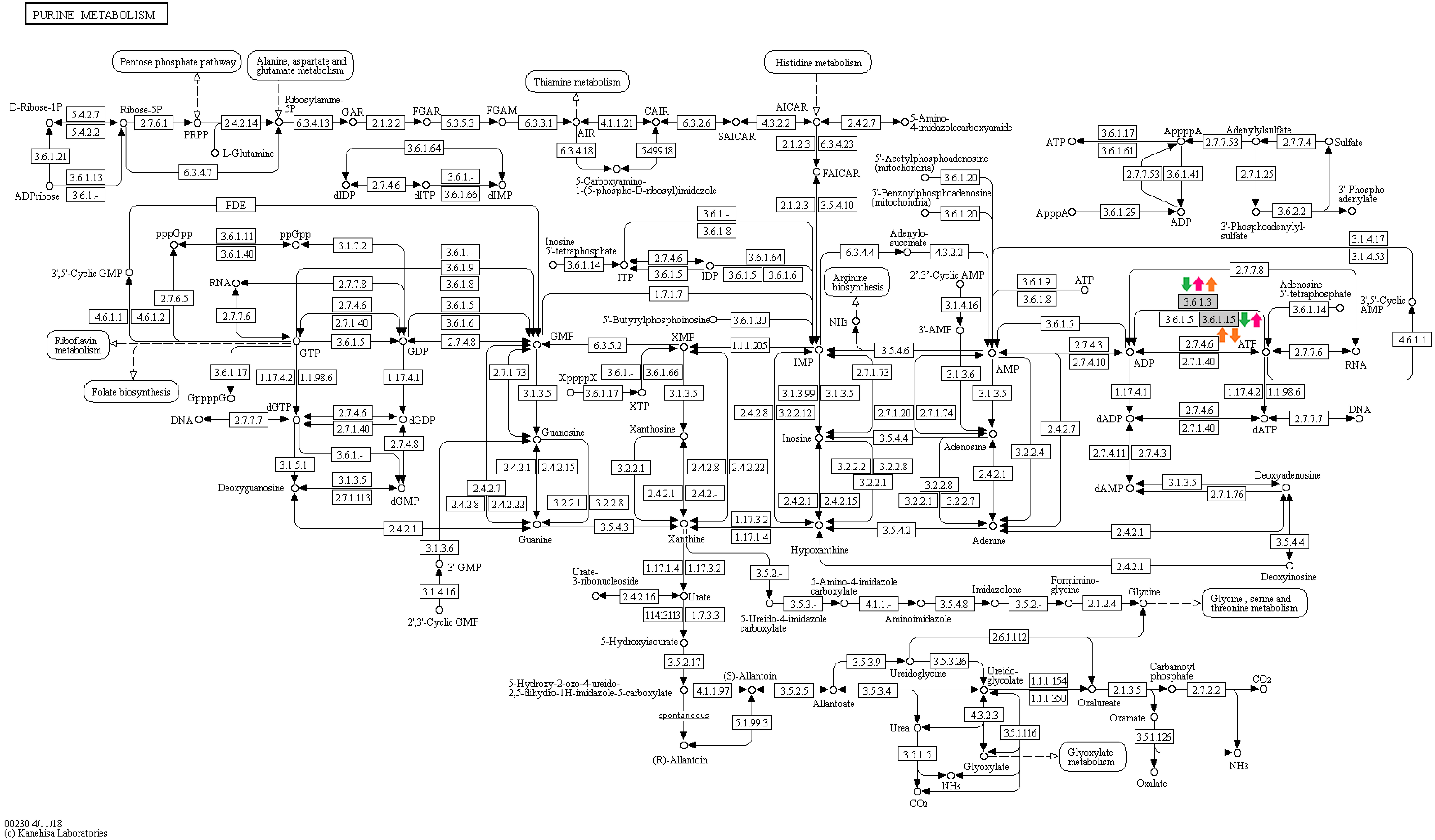
PURINE METABOLISM
Pentose phosphate pathway
Alanine, aspartate and glutamate metabolism
Thiamine metabolism
Histidine metabolism
D-Ribose-1P
Ribose-5P
PRPP
L-Glutamine
Ribosylamine-5P
GAR
FGAR
FGAM
AIR
CAIR
SAICAR
AICAR
5-Amino-4-imidazolecarboxyamide
FAICAR
5-Carboxyamino-1-(5-phospho-D-ribosyl)imidazole
ADPribose
dIDP
dITP
dIMP
PDE
pppGpp
ppGpp
3',5'-Cyclic GMP
RNA
Riboflavin metabolism
Folate biosynthesis
GTP
GDP
GMP
GppppG
dGTP
dGDP
DNA
Deoxyguanosine
dGMP
Guanosine
Guanine
3'-GMP
2',3'-Cyclic GMP
Inosine 5'-tetraphosphate
ITP
IDP
5'-Butyrylphosphoinosine
XMP
XppppX
XTP
Xanthosine
Xanthine
Urate-3-ribonucleoside
Urate
5-Hydroxyisourate
5-Hydroxy-2-oxo-4-ureido-2,5-dihydro-1H-imidazole-5-carboxylate
spontaneous
(S)-Allantoin
(R)-Allantoin
Arginine biosynthesis
NH3
IMP
Inosine
Hypoxanthine
Adenylo-succinate
2',3'-Cyclic AMP
3'-AMP
AMP
Adenosine
Adenine
5'-Acetylphosphoadenosine (mitochondria)
5'-Benzoylphosphoadenosine (mitochondria)
ATP
ApppA
ApppppA
ADP
Adenylylsulfate
Sulfate
3'-Phospho-adenylate
3'-Phosphoadenylyl-sulfate
Adenosine 5'-tetraphosphate
3',5'-Cyclic AMP
dADP
dATP
dAMP
Deoxyadenosine
Deoxyinosine
5-Ureido-4-imidazole carboxylate
5-Amino-4-imidazole carboxylate
Aminoimidazole
Imidazolone
Formimino-glycine
Glycine
Glycine, serine and threonine metabolism
Allantoate
Ureidoglycine
Ureido-glycolate
Urea
Glyoxylate
Glyoxylate metabolism
CO2
Oxalureate
Oxamate
Oxalate
Carbamoyl phosphate
00230 4/11/18
(c) Kanehisa Laboratories

T

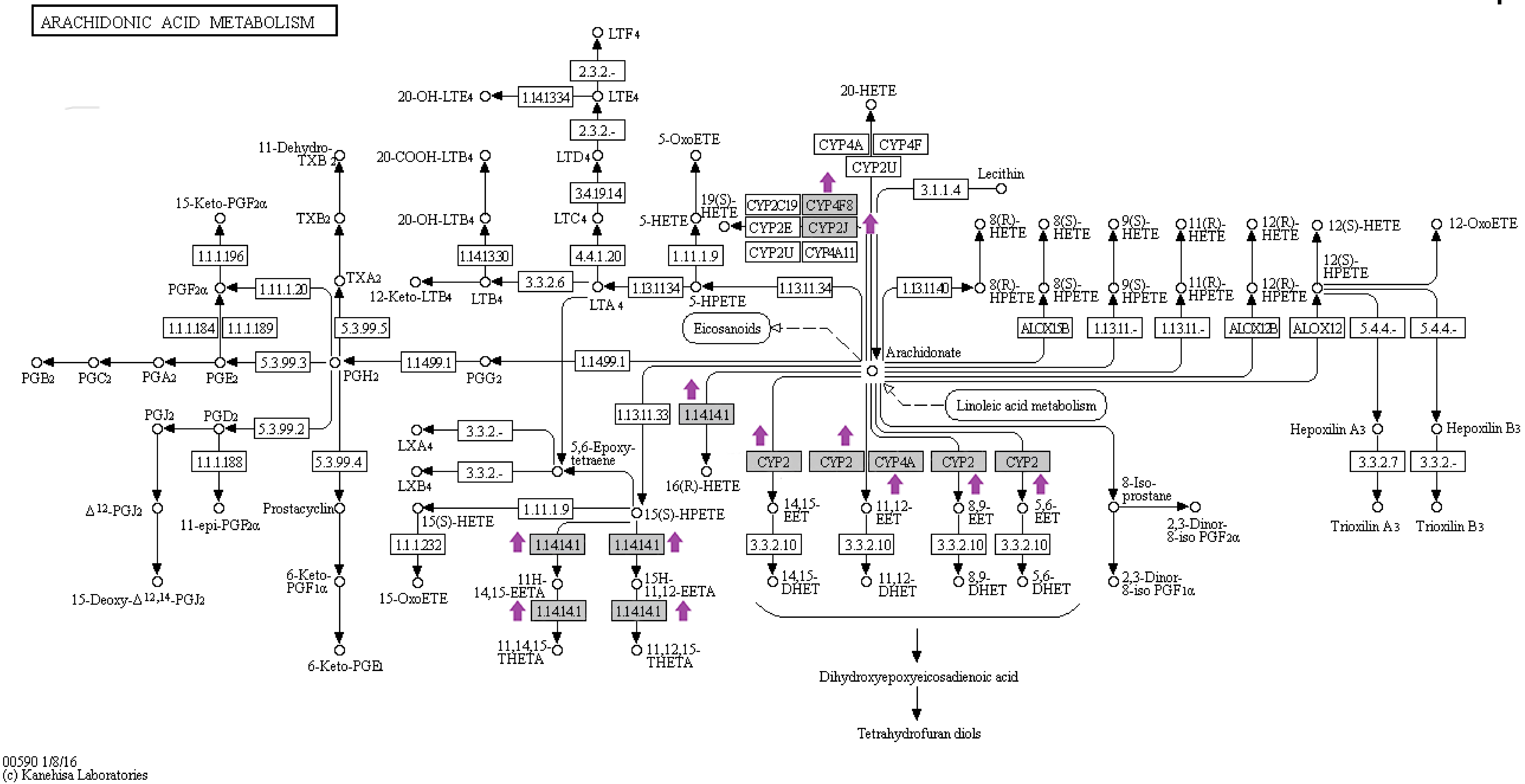

ARACHIDONIC ACID METABOLISM
LTF4
2.3.2.-
20-OH-LTE4
1.14.13.34
LTE4
2.3.2.-
LTD4
3.4.19.14
LTC4
4.4.1.20
LTA4
3.3.2.6
LTB4
12-Keto-LTB4
1.14.13.30
20-OH-LTB4
20-COOH-LTB4
11-Dehydro-TXB2
TXB2
TXA2
5.3.99.5
15-Keto-PGF2α
1.1.1.196
PGF2α
1.1.1.20
1.1.1.184
1.1.1.189
PGB2
PGC2
PGA2
PGE2
5.3.99.3
PGH2
1.14.99.1
PGG2
1.14.99.1
PGJ2
PGD2
5.3.99.2
1.1.1.188
5.3.99.4
Δ12-PGJ2
11-epi-PGF2α
Prostacyclin
15-Deoxy-Δ12,14-PGJ2
6-Keto-PGF1α
6-Keto-PGE1
5-OxoETE
5-HETE
19(S)-HETE
1.1.1.9
1.13.11.34
5-HPETE
1.13.11.34
Eicosanoids
LXA4
3.3.2.-
LXB4
3.3.2.-
5,6-Epoxy-tetraene
1.13.11.33
1.14.14.1
16(R)-HETE
15(S)-HPETE
1.1.1.9
15(S)-HETE
1.1.1.232
15-OxoETE
1.14.14.1
1.14.14.1
11H-14,15-EETA
15H-11,12-EETA
1.14.14.1
1.14.14.1
11,14,15-THETA
11,12,15-THETA
20-HETE
CYP4A
CYP4F
CYP2U
CYP2C19
CYP4F8
CYP2E
CYP2J
CYP2U
CYP4A11
Lecithin
3.1.1.4
Arachidonate
Linoleic acid metabolism
1.13.11.40
8(R)-HETE
8(S)-HETE
9(S)-HETE
11(R)-HETE
12(R)-HETE
12(S)-HETE
12-OxoETE
12(S)-HPETE
8(R)-HPETE
8(S)-HPETE
9(S)-HPETE
11(R)-HPETE
12(R)-HPETE
ALOX15B
1.13.11.-
1.13.11.-
ALOX12B
ALOX12
5.4.4.-
5.4.4.-
Hepoxilin A3
Hepoxilin B3
3.3.2.7
3.3.2.-
Trioxilin A3
Trioxilin B3
CYP2
CYP2
CYP4A
CYP2
CYP2
14,15-EET
11,12-EET
8,9-EET
5,6-EET
3.3.2.10
3.3.2.10
3.3.2.10
3.3.2.10
14,15-DHET
11,12-DHET
8,9-DHET
5,6-DHET
8-Iso-prostane
2,3-Dinor-8-iso PGF2α
2,3-Dinor-8-iso PGF1α
Dihydroxyepoxyeicosadienoic acid
Tetrahydrofuran diols
00590 1/8/16
(c) Kanehisa Laboratories

U

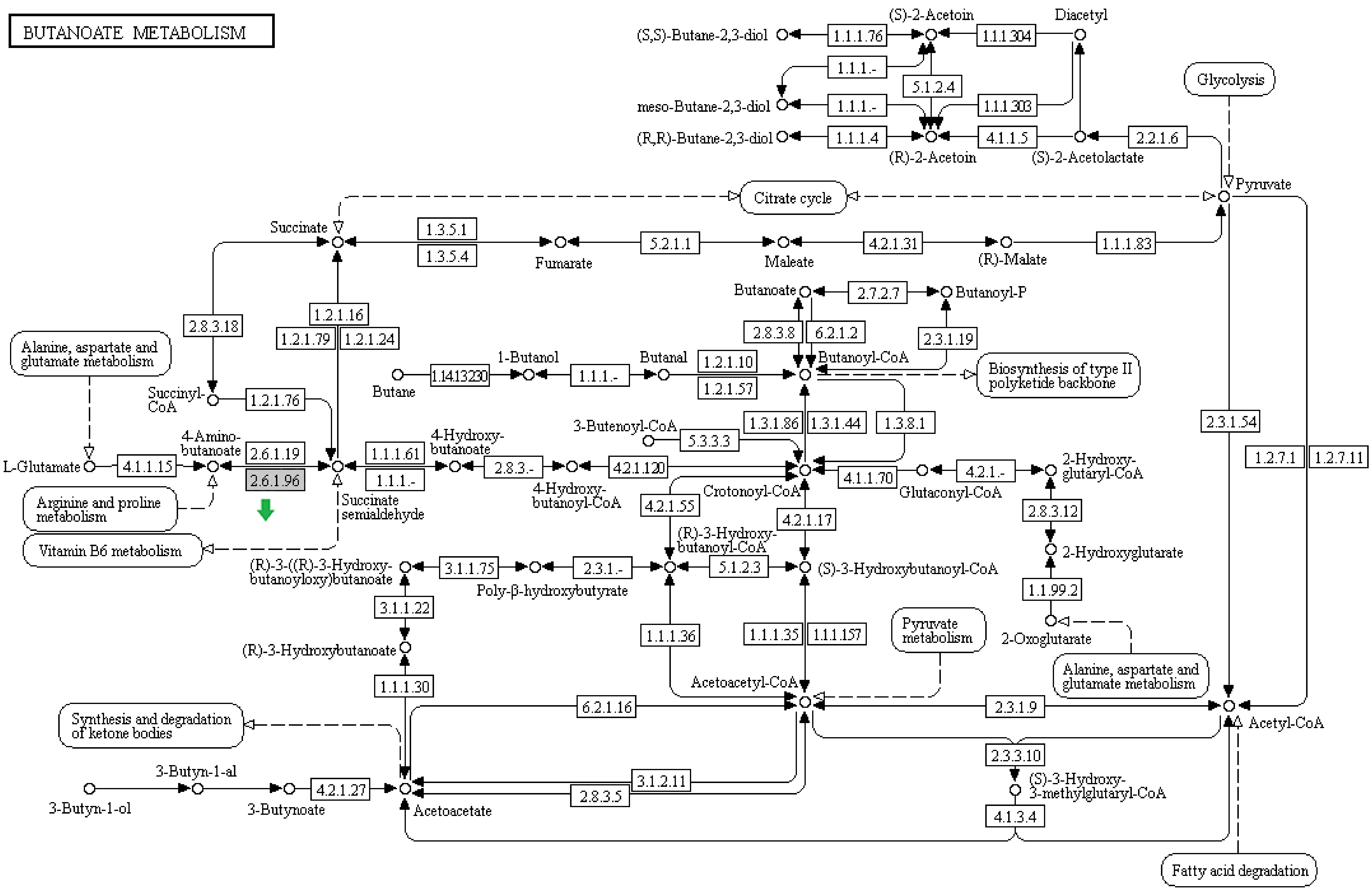

00650 11/1/17

(c) Kanehisa Laboratories

V

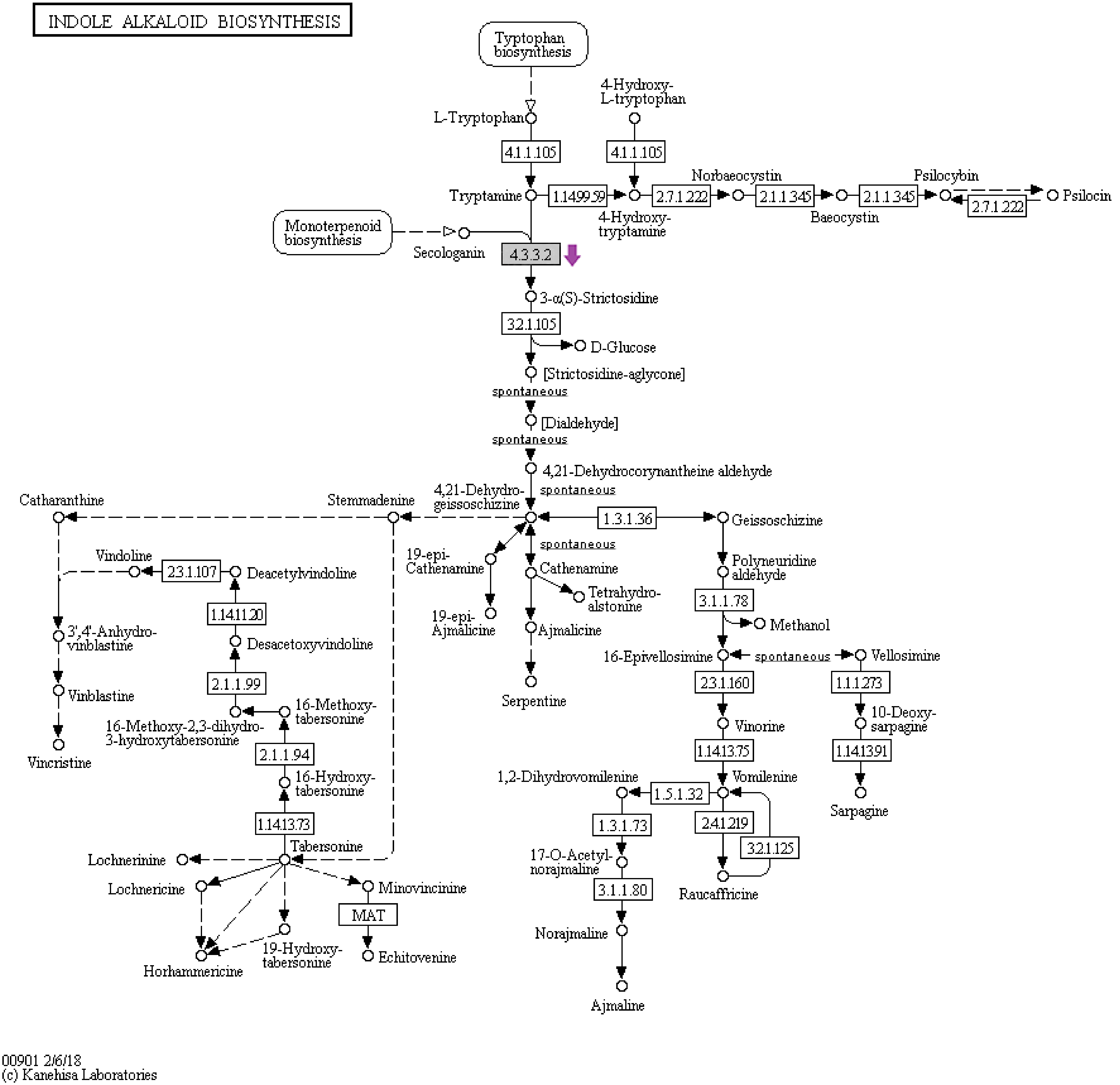
INDOLE ALKALOID BIOSYNTHESIS
Typtophan biosynthesis
L-Tryptophan
4-Hydroxy-L-tryptophan
4.1.1.105
4.1.1.105
Tryptamine
1.14.99.59
4-Hydroxy-tryptamine
2.7.1.222
Norbaeocystin
2.1.1.345
Baeocystin
2.1.1.345
Psilocybin
2.7.1.222
Psilocin
Monoterpenoid biosynthesis
Secologanin
4.3.3.2
3-α(S)-Strictosidine
3.2.1.105
D-Glucose
[Strictosidine-aglycone]
spontaneous
[Dialdehyde]
spontaneous
4,21-Dehydrocorynantheine aldehyde
spontaneous
4,21-Dehydro-geissoschizine
1.3.1.36
Geissoschizine
spontaneous
Catharanthine
Stemmadenine
19-epi-Cathenamine
Cathenamine
Tetrahydro-alstonine
19-epi-Ajmalicine
Ajmalicine
Serpentine
Polyneuridine aldehyde
3.1.1.78
Methanol
16-Epivellosimine
spontaneous
Vellosimine
2.3.1.160
1.1.1.273
Vinorine
10-Deoxy-sarpagine
1.14.13.75
1.14.13.91
Sarpagine
Vomilenine
1,2-Dihydrovomilenine
1.5.1.32
2.4.1.219
3.2.1.125
Raucaffricine
1.3.1.73
17-O-Acetyl-norajmaline
3.1.1.80
Norajmaline
Ajmaline
Vindoline
2.3.1.107
Deacetylvindoline
1.14.11.20
Desacetoxyvindoline
2.1.1.99
3',4'-Anhydro-vinblastine
Vinblastine
Vincristine
16-Methoxy-tabersonine
16-Methoxy-2,3-dihydro-3-hydroxytabersonine
2.1.1.94
16-Hydroxy-tabersonine
1.14.13.73
Tabersonine
Lochnerinine
Lochnericine
Minovincinine
MAT
Echitovenine
19-Hydroxy-tabersonine
Horhammericine
00901 2/6/18
(c) Kanehisa Laboratories

W

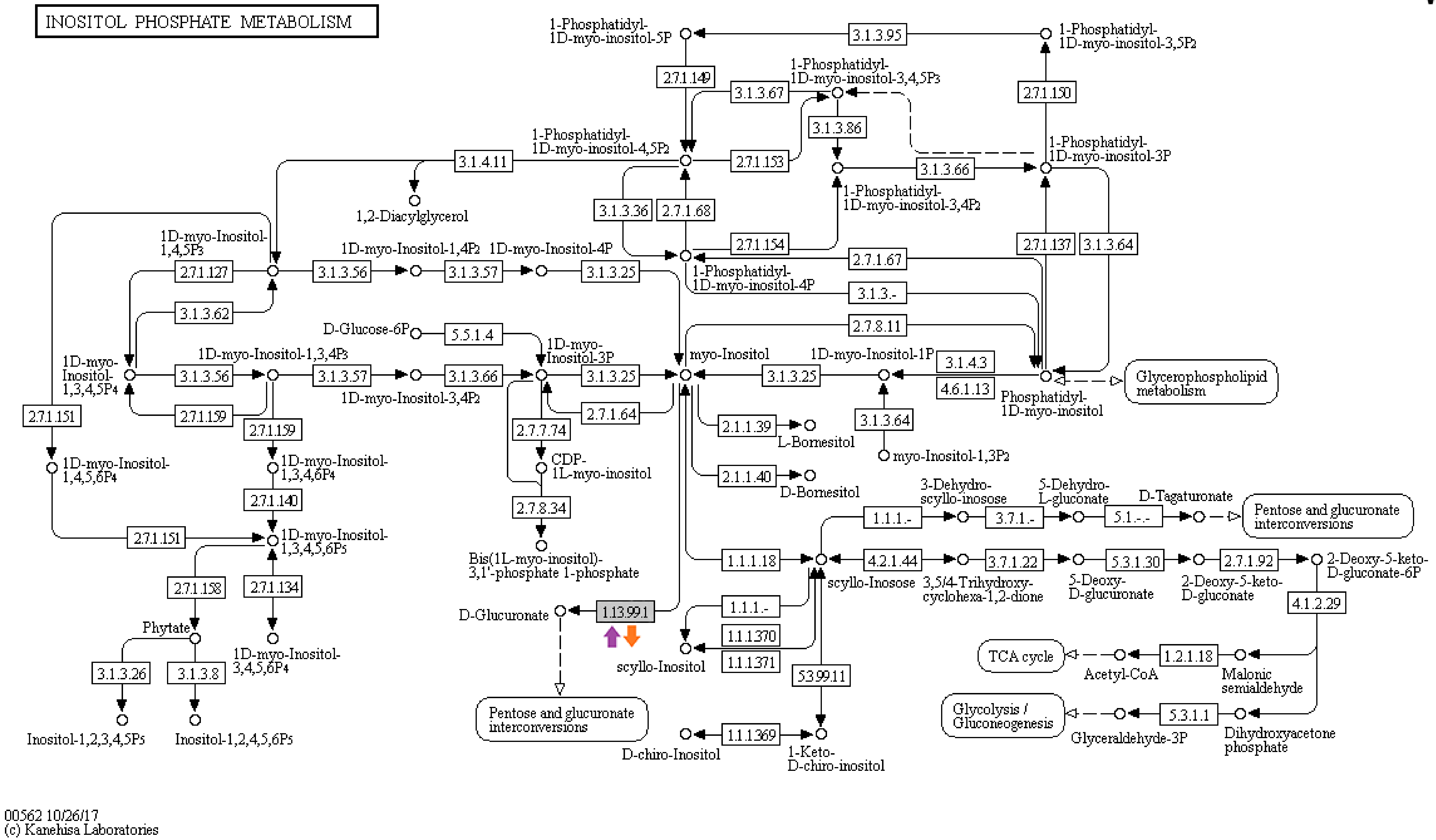
INOSITOL PHOSPHATE METABOLISM
1-Phosphatidyl-1D-myo-inositol-5P
3.1.3.95
1-Phosphatidyl-1D-myo-inositol-3,5P2
2.7.1.149
3.1.3.67
1-Phosphatidyl-1D-myo-inositol-3,4,5P3
2.7.1.150
3.1.3.86
1-Phosphatidyl-1D-myo-inositol-4,5P2
3.1.4.11
2.7.1.153
3.1.3.66
1-Phosphatidyl-1D-myo-inositol-3P
1,2-Diacylglycerol
3.1.3.36
2.7.1.68
1-Phosphatidyl-1D-myo-inositol-3,4P2
1D-myo-Inositol-1,4,5P3
1D-myo-Inositol-1,4P2
1D-myo-Inositol-4P
2.7.1.154
2.7.1.67
2.7.1.137
3.1.3.64
2.7.1.127
3.1.3.56
3.1.3.57
3.1.3.25
1-Phosphatidyl-1D-myo-inositol-4P
3.1.3.-
3.1.3.62
D-Glucose-6P
5.5.1.4
2.7.8.11
1D-myo-Inositol-3P
myo-Inositol
1D-myo-Inositol-1P
3.1.4.3
4.6.1.13
1D-myo-Inositol-1,3,4P3
Glycerophospholipid metabolism
1D-myo-Inositol-1,3,4,5P4
3.1.3.56
3.1.3.57
3.1.3.66
3.1.3.25
3.1.3.25
Phosphatidyl-1D-myo-inositol
1D-myo-Inositol-3,4P2
2.7.1.151
2.7.1.159
2.7.1.159
2.7.7.74
2.7.1.64
2.1.1.39
L-Bornesitol
3.1.3.64
myo-Inositol-1,3P2
1D-myo-Inositol-1,4,5,6P4
1D-myo-Inositol-1,3,4,6P4
CDP-1L-myo-inositol
2.1.1.40
D-Bornesitol
3-Dehydro-scyllo-inosose
5-Dehydro-L-gluconate
D-Tagaturonate
Pentose and glucuronate interconversions
2.7.1.140
2.7.8.34
1.1.1.-
3.7.1.-
5.1.-.-
2.7.1.151
1D-myo-Inositol-1,3,4,5,6P5
Bis(1L-myo-inositol)-3,1'-phosphate 1-phosphate
1.1.1.18
4.2.1.44
3.7.1.22
5.3.1.30
2.7.1.92
2-Deoxy-5-keto-D-gluconate-6P
2.7.1.158
2.7.1.134
scyllo-Inosose
3,5/4-Trihydroxy-cyclohexa-1,2-dione
5-Deoxy-D-glucuronate
2-Deoxy-5-keto-D-gluconate
4.1.2.29
D-Glucuronate
1.13.99.1
1.1.1.-
Phytate
1D-myo-Inositol-3,4,5,6P4
1.1.1.370
scyllo-Inositol
1.1.1.371
TCA cycle
Acetyl-CoA
1.2.1.18
Malonic semialdehyde
3.1.3.26
3.1.3.8
5.3.99.11
Glycolysis / Gluconeogenesis
5.3.1.1
Pentose and glucuronate interconversions
Inositol-1,2,3,4,5P5
Inositol-1,2,4,5,6P5
D-chiro-Inositol
1.1.1.369
1-Keto-D-chiro-inositol
Glyceraldehyde-3P
Dihydroxyacetone phosphate
00562 10/26/17
(c) Kanehisa Laboratories

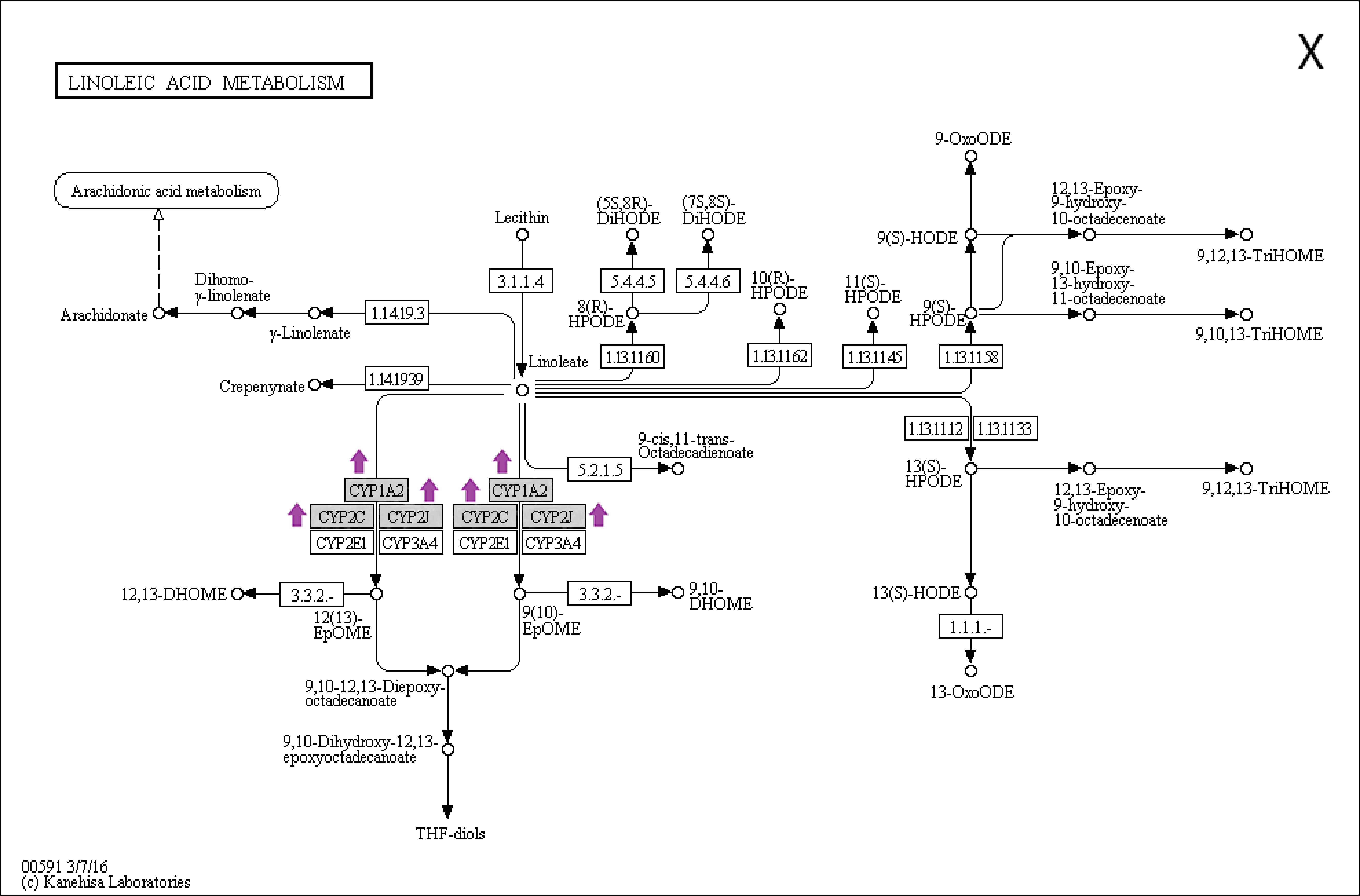
X
LINOLEIC ACID METABOLISM
Arachidonic acid metabolism
Arachidonate
Dihomo-
γ-linolenate
γ-Linolenate
1.14.19.3
Lecithin
3.1.1.4
(5S,8R)-
DiHODE
(7S,8S)-
DiHODE
5.4.4.5
5.4.4.6
8(R)-
HPODE
10(R)-
HPODE
11(S)-
HPODE
9-OxoODE
9(S)-HODE
9(S)-
HPODE
12,13-Epoxy-
9-hydroxy-
10-octadecenoate
9,12,13-TriHOME
9,10-Epoxy-
13-hydroxy-
11-octadecenoate
9,10,13-TriHOME
1.13.11.60
1.13.11.62
1.13.11.45
1.13.11.58
Crepenynate
1.14.19.39
Linoleate
1.13.11.12
1.13.11.33
13(S)-
HPODE
12,13-Epoxy-
9-hydroxy-
10-octadecenoate
9,12,13-TriHOME
9-cis,11-trans-
Octadecadienoate
5.2.1.5
CYP1A2
CYP2C
CYP2J
CYP2E1
CYP3A4
CYP1A2
CYP2C
CYP2J
CYP2E1
CYP3A4
12,13-DHOME
3.3.2.-
12(13)-
EpOME
3.3.2.-
9,10-
DHOME
9(10)-
EpOME
13(S)-HODE
1.1.1.-
13-OxoODE
9,10-12,13-Diepoxy-
octadecanoate
9,10-Dihydroxy-12,13-
epoxyoctadecanoate
THF-diols
00591 3/7/16
(c) Kanehisa Laboratories

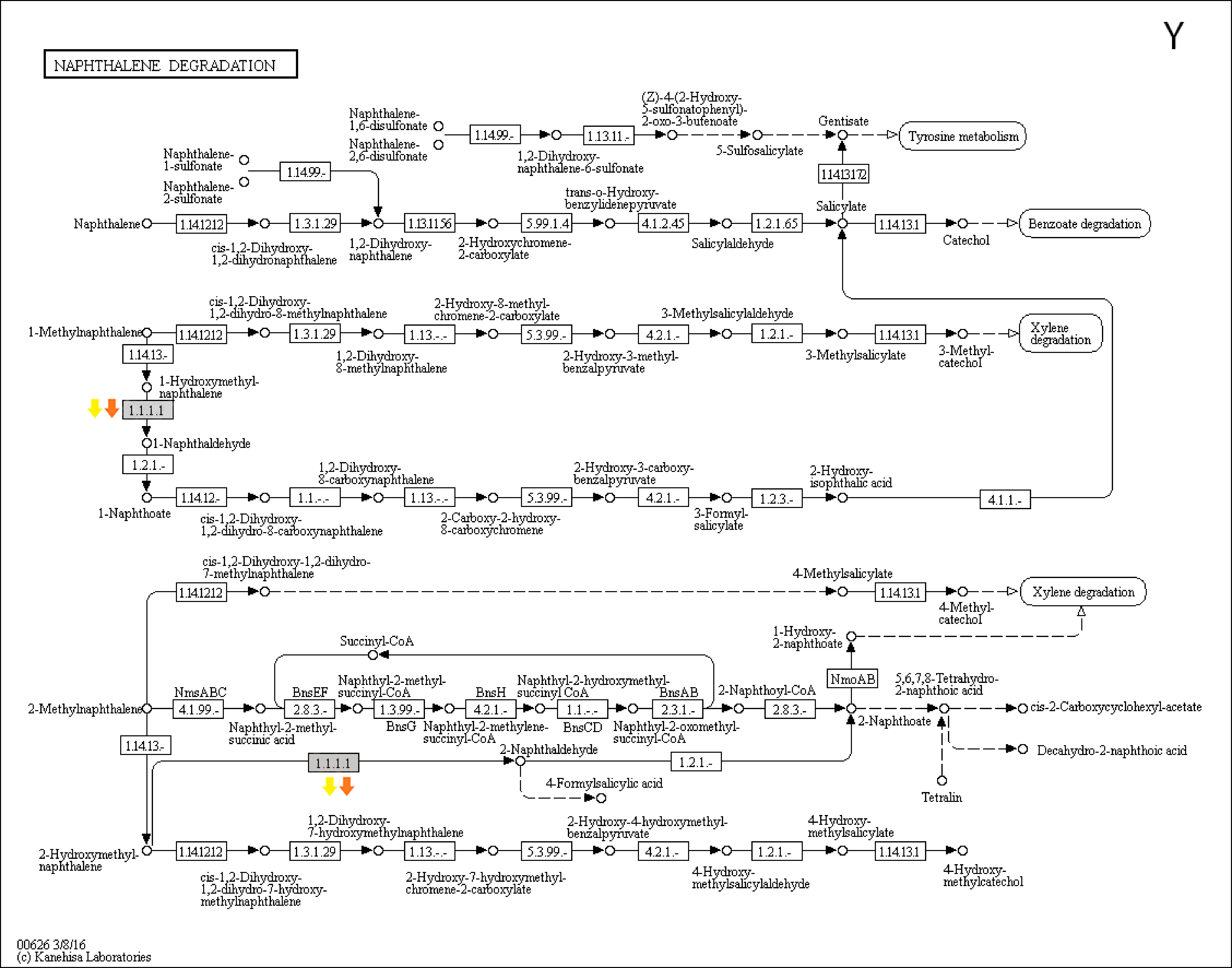
Y
NAPHTHALENE DEGRADATION
Naphthalene-1,6-disulfonate
Naphthalene-2,6-disulfonate
1.14.99.-
1.13.11.-
(Z)-4-(2-Hydroxy-5-sulfonatophenyl)-2-oxo-3-butenoate
Gentisate
Tyrosine metabolism
1,2-Dihydroxy-naphthalene-6-sulfonate
5-Sulfosalicylate
1.14.13.172
Naphthalene-1-sulfonate
Naphthalene-2-sulfonate
1.14.99.-
Naphthalene
1.14.12.12
1.3.1.29
1.13.11.56
5.99.1.4
4.1.2.45
1.2.1.65
1.14.13.1
trans-o-Hydroxy-benzylidenepyruvate
Salicylate
Benzoate degradation
cis-1,2-Dihydroxy-1,2-dihydronaphthalene
1,2-Dihydroxy-naphthalene
2-Hydroxychromene-2-carboxylate
Salicylaldehyde
Catechol
cis-1,2-Dihydroxy-1,2-dihydro-8-methylnaphthalene
2-Hydroxy-8-methyl-chromene-2-carboxylate
3-Methylsalicylaldehyde
1-Methylnaphthalene
1.14.12.12
1.3.1.29
1.13.-.-
5.3.99.-
4.2.1.-
1.2.1.-
1.14.13.1
Xylene degradation
1.14.13.-
1,2-Dihydroxy-8-methylnaphthalene
2-Hydroxy-3-methyl-benzalpyruvate
3-Methylsalicylate
3-Methyl-catechol
1-Hydroxymethyl-naphthalene
1.1.1.1
1-Naphthaldehyde
1.2.1.-
1,2-Dihydroxy-8-carboxynaphthalene
2-Hydroxy-3-carboxy-benzalpyruvate
2-Hydroxy-isophthalic acid
1-Naphthoate
1.14.12.-
1.1.-.-
1.13.-.-
5.3.99.-
4.2.1.-
1.2.3.-
4.1.1.-
cis-1,2-Dihydroxy-1,2-dihydro-8-carboxynaphthalene
2-Carboxy-2-hydroxy-8-carboxychromene
3-Formyl-salicylate
cis-1,2-Dihydroxy-1,2-dihydro-7-methylnaphthalene
4-Methylsalicylate
1.14.12.12
1.14.13.1
4-Methyl-catechol
Xylene degradation
1-Hydroxy-2-naphthoate
Succinyl-CoA
NmoAB
NmsABC
BnsEF
Naphthyl-2-methyl-succinyl-CoA
BnsH
Naphthyl-2-hydroxymethyl-succinyl CoA
BnsAB
2-Naphthoyl-CoA
5,6,7,8-Tetrahydro-2-naphthoic acid
2-Methylnaphthalene
4.1.99.-
2.8.3.-
1.3.99.-
4.2.1.-
1.1.-.-
2.3.1.-
2.8.3.-
cis-2-Carboxycyclohexyl-acetate
Naphthyl-2-methyl-succinic acid
BnsG
Naphthyl-2-methylene-succinyl-CoA
BnsCD
Naphthyl-2-oxomethyl-succinyl-CoA
2-Naphthoate
1.14.13.-
1.1.1.1
2-Naphthaldehyde
1.2.1.-
Decahydro-2-naphthoic acid
4-Formylsalicylic acid
Tetralin
1,2-Dihydroxy-7-hydroxymethylnaphthalene
2-Hydroxy-4-hydroxymethyl-benzalpyruvate
4-Hydroxy-methylsalicylate
2-Hydroxymethyl-naphthalene
1.14.12.12
1.3.1.29
1.13.-.-
5.3.99.-
4.2.1.-
1.2.1.-
1.14.13.1
cis-1,2-Dihydroxy-1,2-dihydro-7-hydroxy-methylnaphthalene
2-Hydroxy-7-hydroxymethyl-chromene-2-carboxylate
4-Hydroxy-methylsalicylaldehyde
4-Hydroxy-methylcatechol
00626 3/8/16
(c) Kanehisa Laboratories

Z

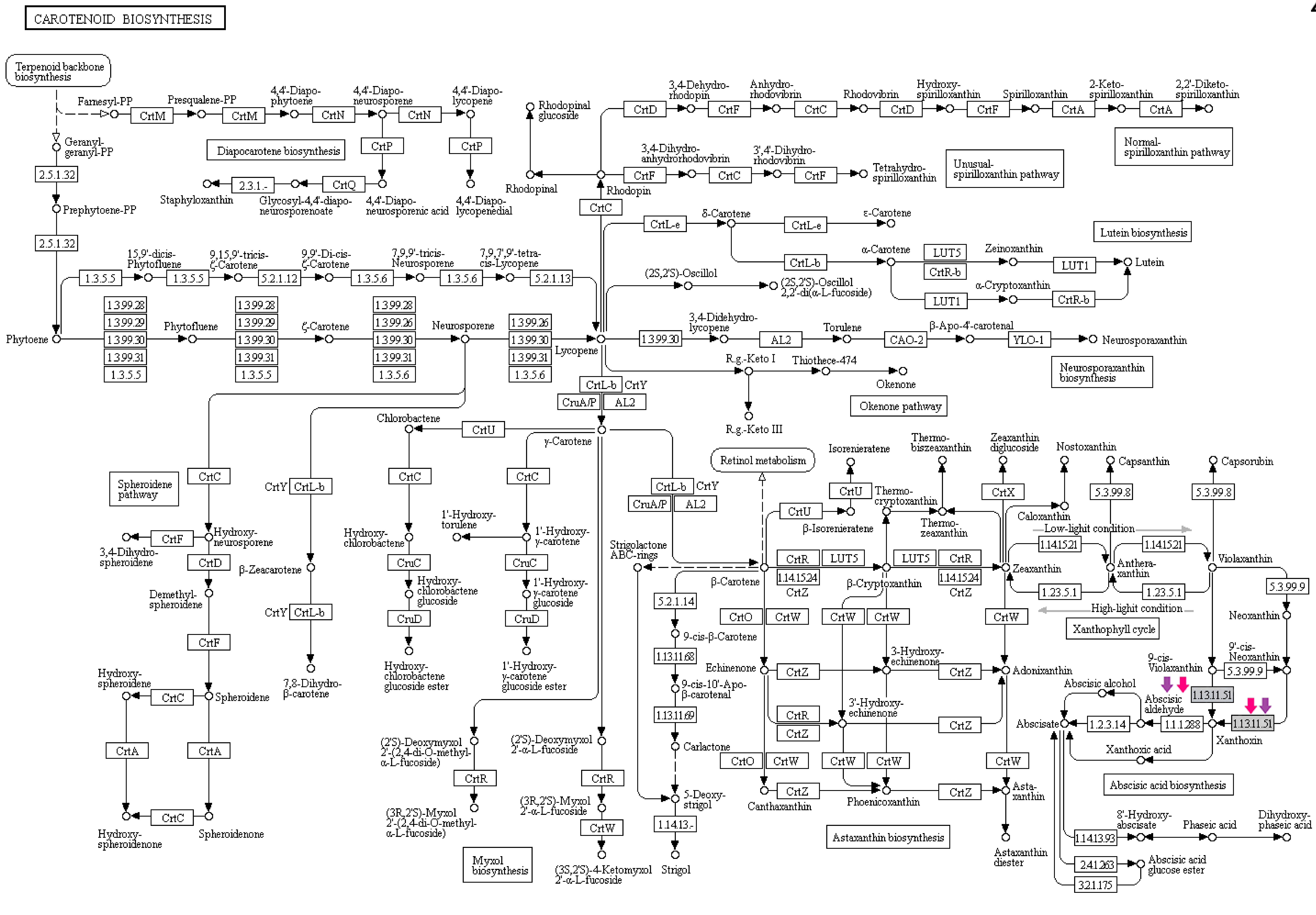

00906 3/27/18
(c) Kanehisa Laboratories

AA

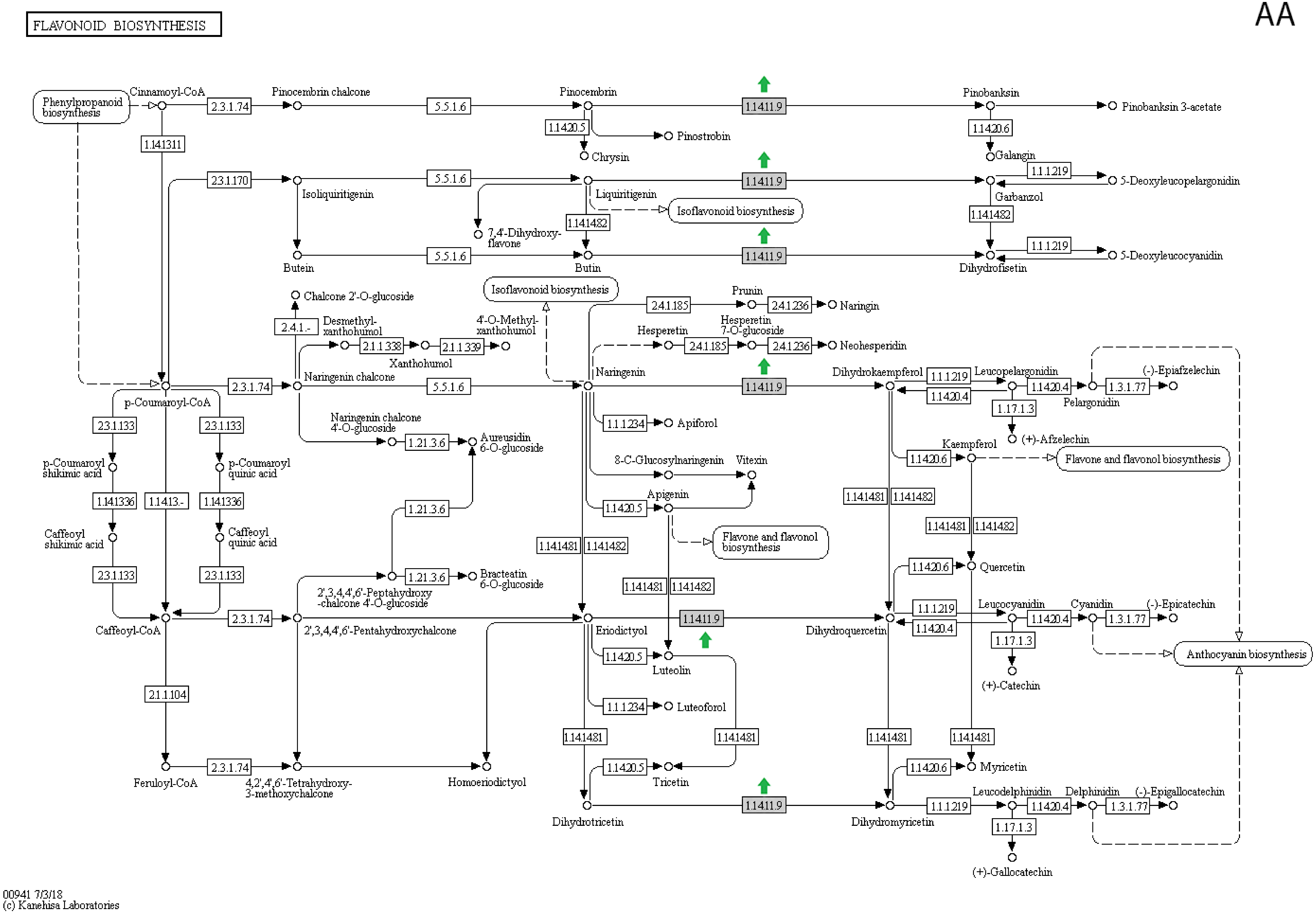
FLAVONOID BIOSYNTHESIS
Phenylpropanoid biosynthesis
Cinnamoyl-CoA
2.3.1.74
Pinocembrin chalcone
5.5.1.6
Pinocembrin
1.14.11.9
Pinobanksin
Pinobanksin 3-acetate
1.14.13.11
1.14.20.5
Pinostrobin
Chrysin
1.14.20.6
Galangin
2.3.1.170
Isoliquiritigenin
Liquiritigenin
Isoflavonoid biosynthesis
1.1.1.219
5-Deoxyleucopelargonidin
Garbanzol
1.14.14.82
7,4'-Dihydroxy-flavone
Butein
Butin
Dihydrofisetin
5-Deoxyleucocyanidin
Chalcone 2'-O-glucoside
2.4.1.-
Desmethyl-xanthohumol
4'-O-Methyl-xanthohumol
2.1.1.338
2.1.1.339
Xanthohumol
2.4.1.185
Prunin
2.4.1.236
Naringin
Hesperetin
Hesperetin 7-O-glucoside
Neohesperidin
Naringenin chalcone
Naringenin
p-Coumaroyl-CoA
Dihydrokaempferol
Leucopelargonidin
1.14.20.4
Pelargonidin
1.3.1.77
(-)-Epiafzelechin
1.17.1.3
(+)-Afzelechin
2.3.1.133
p-Coumaroyl shikimic acid
p-Coumaroyl quinic acid
Naringenin chalcone 4'-O-glucoside
1.21.3.6
Aureusidin 6-O-glucoside
1.1.1.234
Apiforol
Kaempferol
Flavone and flavonol biosynthesis
8-C-Glucosylnaringenin
Vitexin
Apigenin
1.14.13.36
1.14.13.-
Caffeoyl shikimic acid
Caffeoyl quinic acid
1.14.14.81
Quercetin
Bracteatin 6-O-glucoside
2',3,4,4',6'-Peptahydroxy-chalcone 4'-O-glucoside
Caffeoyl-CoA
2',3,4,4',6'-Pentahydroxychalcone
Eriodictyol
Dihydroquercetin
Leucocyanidin
Cyanidin
(-)-Epicatechin
Anthocyanin biosynthesis
Luteolin
(+)-Catechin
2.1.1.104
Luteoforol
Feruloyl-CoA
4,2',4',6'-Tetrahydroxy-3-methoxychalcone
Homoeriodictyol
Tricetin
Myricetin
Dihydrotricetin
Dihydromyricetin
Leucodelphinidin
Delphinidin
(-)-Epigallocatechin
(+)-Gallocatechin
00941 7/3/18
(c) Kanehisa Laboratories

BB

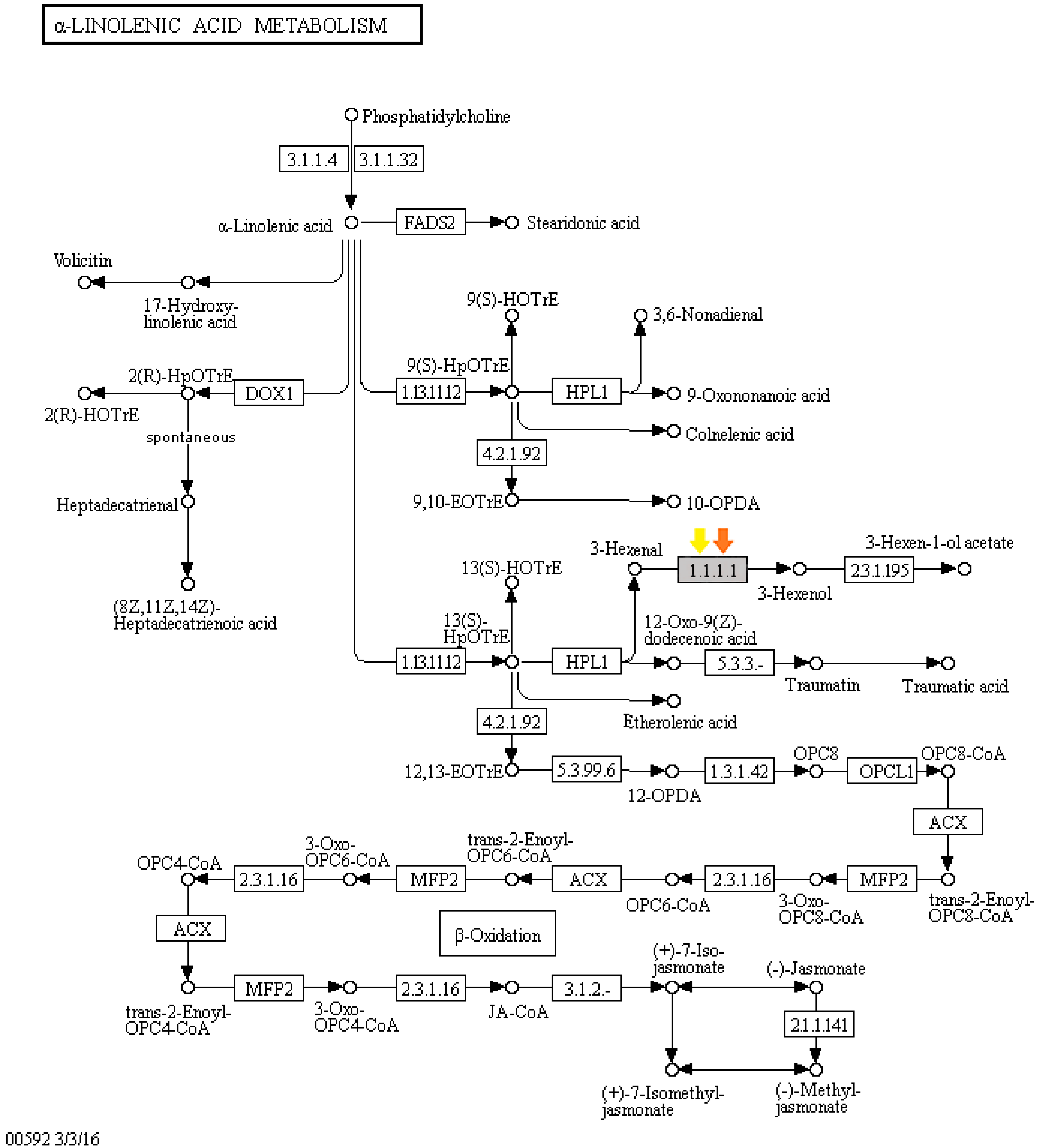

00592 3/3/16
(c) Kanehisa Laboratories

CC

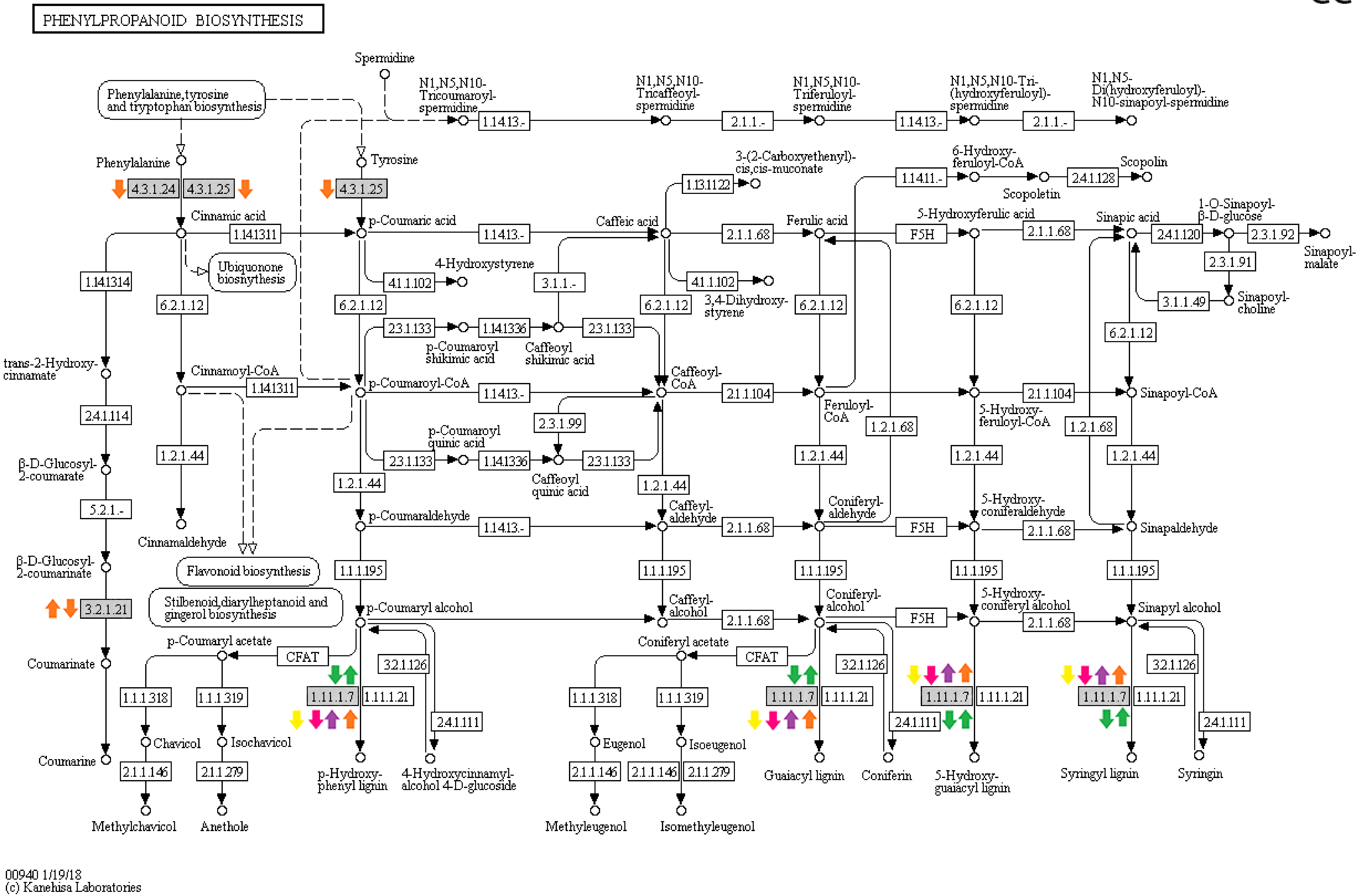
PHENYLPROPANOID BIOSYNTHESIS
Spermidine
Phenylalanine,tyrosine and tryptophan biosynthesis
N1,N5,N10-Tricoumaroyl-spermidine
1.14.13.-
N1,N5,N10-Tricaffeoyl-spermidine
2.1.1.-
N1,N5,N10-Triferuloyl-spermidine
1.14.13.-
N1,N5,N10-Tri-(hydroxyferuloyl)-spermidine
2.1.1.-
N1,N5-Di(hydroxyferuloyl)-N10-sinapoyl-spermidine
Phenylalanine
Tyrosine
4.3.1.24
4.3.1.25
4.3.1.25
3-(2-Carboxyethenyl)-cis,cis-muconate
1.13.11.22
6-Hydroxy-feruloyl-CoA
1.14.11.-
Scopoletin
2.4.1.128
Scopolin
Cinnamic acid
1.14.13.11
p-Coumaric acid
1.14.13.-
Caffeic acid
2.1.1.68
Ferulic acid
5-Hydroxyferulic acid
F5H
2.1.1.68
Sinapic acid
2.4.1.120
1-O-Sinapoyl-β-D-glucose
2.3.1.92
Sinapoyl-malate
2.3.1.91
3.1.1.49
Sinapoyl-choline
1.14.13.14
Ubiquonone biosynthesis
4-Hydroxystyrene
4.1.1.102
3.1.1.-
4.1.1.102
3,4-Dihydroxy-styrene
6.2.1.12
6.2.1.12
6.2.1.12
6.2.1.12
6.2.1.12
6.2.1.12
2.3.1.133
1.14.13.36
2.3.1.133
p-Coumaroyl shikimic acid
Caffeoyl shikimic acid
trans-2-Hydroxy-cinnamate
Cinnamoyl-CoA
1.14.13.11
p-Coumaroyl-CoA
1.14.13.-
Caffeoyl-CoA
2.1.1.104
Feruloyl-CoA
2.1.1.104
5-Hydroxy-feruloyl-CoA
Sinapoyl-CoA
2.4.1.114
2.3.1.99
p-Coumaroyl quinic acid
1.2.1.68
1.2.1.68
β-D-Glucosyl-2-coumarate
1.2.1.44
2.3.1.133
1.14.13.36
2.3.1.133
1.2.1.44
1.2.1.44
1.2.1.44
1.2.1.44
Caffeoyl quinic acid
1.2.1.44
5.2.1.-
Caffeyl-aldehyde
Coniferyl-aldehyde
5-Hydroxy-coniferaldehyde
p-Coumaraldehyde
1.14.13.-
2.1.1.68
F5H
2.1.1.68
Sinapaldehyde
Cinnamaldehyde
β-D-Glucosyl-2-coumarinate
Flavonoid biosynthesis
1.1.1.195
1.1.1.195
1.1.1.195
1.1.1.195
1.1.1.195
3.2.1.21
Stilbenoid,diarylheptanoid and gingerol biosynthesis
p-Coumaryl alcohol
Caffeyl-alcohol
2.1.1.68
Coniferyl-alcohol
F5H
5-Hydroxy-coniferyl alcohol
2.1.1.68
Sinapyl alcohol
p-Coumaryl acetate
CFAT
Coniferyl acetate
CFAT
Coumarinate
3.2.1.126
3.2.1.126
3.2.1.126
1.1.1.318
1.1.1.319
1.11.1.7
1.11.1.21
1.1.1.318
1.1.1.319
1.11.1.7
1.11.1.21
1.11.1.7
1.11.1.21
1.11.1.7
1.11.1.21
2.4.1.111
2.4.1.111
2.4.1.111
Chavicol
Isochavicol
Eugenol
Isoeugenol
Coumarine
2.1.1.146
2.1.1.279
2.1.1.146
2.1.1.146
2.1.1.279
p-Hydroxy-phenyl lignin
4-Hydroxycinnamyl-alcohol 4-D-glucoside
Guaiacyl lignin
Coniferin
5-Hydroxy-guaiacyl lignin
Syringyl lignin
Syringin
Methylchavicol
Anethole
Methyleugenol
Isomethyleugenol
00940 1/19/18
(c) Kanehisa Laboratories
